# Life Cycle Assessment of Metals: A Scientific Synthesis

**DOI:** 10.1371/journal.pone.0101298

**Published:** 2014-07-07

**Authors:** Philip Nuss, Matthew J. Eckelman

**Affiliations:** 1 Center for Industrial Ecology, School of Forestry and Environmental Studies, Yale University, New Haven, Connecticut, United States of America; 2 Department of Civil and Environmental Engineering, Northeastern University, Boston, Massachusetts, United States of America; Belgian Nuclear Research Centre SCK•CEN, Belgium

## Abstract

We have assembled extensive information on the cradle-to-gate environmental burdens of 63 metals in their major use forms, and illustrated the interconnectedness of metal production systems. Related cumulative energy use, global warming potential, human health implications and ecosystem damage are estimated by metal life cycle stage (i.e., mining, purification, and refining). For some elements, these are the first life cycle estimates of environmental impacts reported in the literature. We show that, if compared on a per kilogram basis, the platinum group metals and gold display the highest environmental burdens, while many of the major industrial metals (e.g., iron, manganese, titanium) are found at the lower end of the environmental impacts scale. If compared on the basis of their global annual production in 2008, iron and aluminum display the largest impacts, and thallium and tellurium the lowest. With the exception of a few metals, environmental impacts of the majority of elements are dominated by the purification and refining stages in which metals are transformed from a concentrate into their metallic form. Out of the 63 metals investigated, 42 metals are obtained as co-products in multi output processes. We test the sensitivity of varying allocation rationales, in which the environmental burden are allocated to the various metal and mineral products, on the overall results. Monte-Carlo simulation is applied to further investigate the stability of our results. This analysis is the most comprehensive life cycle comparison of metals to date and allows for the first time a complete bottom-up estimate of life cycle impacts of the metals and mining sector globally. We estimate global direct and indirect greenhouse gas emissions in 2008 at 3.4 Gt CO_2_-eq per year and primary energy use at 49 EJ per year (9.5% of global use), and report the shares for all metals to both impact categories.

## Introduction

Metals are ubiquitous in today's society; there are few materials or products where metals are absent or have not played a role in their production. While a century ago, the diversity of metals employed was limited to perhaps a dozen in common uses such as infrastructure and durable goods, today's technologies utilizes virtually the entire periodic table [1], [2]. For example, the number of elements employed in integrated circuits used in most electronics products has increased from only twelve elements in 1980 to more than sixty elements today [1], while electronic products themselves are used in an increasing number of applications [2]. Similarly, the elemental complexity of superalloys, which are a class of materials to allow the operation of turbines and jet engines at high temperatures and under corrosive environments, has increased over time as new alloying elements (e.g., rhenium, tantalum, hafnium) are added.

Future global demand for metals is expected to increase further as a result of urbanization and new infrastructure construction in developing countries, widespread use of electronics, and transitions in energy technologies [3]. The use of renewable energy technologies, such as photovoltaic and wind power, is expected to result in an increased demand for both bulk metals (e.g., iron, copper) and specialty metals (e.g., rare earths) when compared to today's largely fossil-based systems [4], [5]. While increased future demand for primary metals could be reduced through dematerialization, substitution with other metallic or non-metallic resources [6], and increased metals end-of-life recycling rates, a recent study by Graedel et al. [7] indicates that current end-of-life recycling rates for only eighteen metals (out of a total of sixty) are above 50%. These include silver, aluminum, gold, chromium, copper, iron, manganese, niobium, nickel, lead, palladium, platinum, rhenium, rhodium, tin, titanium, and zinc. For many of the specialty metals, such as scandium and yttrium, as well as the rare earth elements, end-of-life recycling rates were found to be less than 1%.

The production of primary (virgin) metal typically includes ore mining and concentrating, smelting or separation, and refining to obtain the element in its metallic form [8], [9], with a variety of processing routes available. In each stage, impurities and by-products are separated and the concentration of the metal in the final product increases. Metal refining to sufficient purities frequently requires energy-intensive and precisely-controlled melting stages, often based on the use of fossil-fuel inputs directly as a reductant or indirectly for heat and electricity. In 2007, iron and steel production accounted for 30% and aluminum for 2% of global industrial carbon dioxide (CO_2_) emissions (out of a total of 7.6 Gt CO_2_) [10]. Pyrometallurgy involves treatment of metal concentrates at high temperatures, in order to strip the metal from its associated mineral constituents, using fossil fuels in heating furnaces or electricity to power an electric arc furnace [8], [9]. Hydrometallurgy consists of treating metal ores or concentrates in liquid solution to separate metals from their associated minerals [8], [9]. While high temperatures are not usually required, treatment may take place at high pressures which requires energy to maintain and the provision of liquid agents. A number of studies indicate that the energy intensity of the mining and beneficiation process is likely to increase over time as mines shift from high- to lower-grade metal ores and start mining more complex deposits (downstream metal extraction and refining, however, is likely to be unaffected) [11]–[13]. This trend may be partially offset by increased process efficiencies. It is expected that future supplies of metals will increasingly consist of non-traditional geological metal resources such as seabed nodules and crustal rock [14].

The metals production system is highly interconnected, consisting of various processes in which the production or recovery of multiple metals occurs simultaneously, as in lead-zinc ores for example [15]. While the environmental implications of the major industrial metals (e.g., iron and copper) have been extensively studied [3], the environmental burdens of many of the minor metals (e.g., niobium, rhenium, hafnium) are essentially unknown, even though they are increasingly employed by industry. Materials scientists and product developers have a growing number of tools available that allow them to consider the environmental implications of their choices in materials, but in general these tools consider a small number of environmental endpoints, and many data gaps remain. However, given the expected increase in global future demand for metals and their importance in today's technologies, it is important that high-fidelity data for life cycle based environmental burdens of metals production are available and that the implications of co-production are clearly understood. Quantifying the environmental burdens per life cycle stage and the interconnectedness of the metals production systems is required in modeling global changes in technology, material substitution and metals criticality in terms of their supply chain vulnerability and supply risk [16]–[22]. A comprehensive understanding allows us to better manage the impacts and benefits of metals and inform sustainable resource use.

Life cycle assessment (LCA) [23], [24] can be used as a tool to quantify the system-wide (cradle-to-gate or cradle-to-grave) environmental burdens of products, services, and technologies. A significant body of research on the life-cycle wide energy use and wider environmental impacts of metals provision is available from various life cycle inventory (LCI) databases [25]–[38], scientific reports, and publications [3]. However, many LCI data are reported in an aggregated form (either pre-allocated or at system process level), and this makes it difficult to make robust comparisons or to take co-production issues into account. LCI data are also not always representative of global metals production routes and the chemical forms of an element going into use (a metal used in its metallic or mineral form), and may be outdated if representing older technologies. Furthermore, to our knowledge the conventional LCI databases do not report metals data for a number of elements including Be, Sc, Ge, Sr, Zr, Nb, Ru, Ba, Hf, Re, Os, Ir, Bi, and Th, which are increasingly applied in today's technologies.

Against this background, the goal of this study is to present an overview of the cradle-to-gate environmental burdens of 63 metals (plus helium) in their major use forms, including (by increasing atomic number) helium (He), lithium (Li), beryllium (Be), boron (B), magnesium (Mg), aluminum (Al), calcium (Ca), scandium (Sc), titanium (Ti), vanadium (V), chromium (Cr), manganese (Mn), iron (Fe), cobalt (Co), nickel (Ni), copper (Cu), zinc (Zn), gallium (Ga), germanium (Ge), arsenic (As), selenium (Se), strontium (Sr), yttrium (Y), zirconium (Zr), niobium (Nb), molybdenum (Mo), ruthenium (Ru), rhodium (Rh), palladium (Pd), silver (Ag), cadmium (Cd), indium (In), tin (Sn), antimony (Sb), tellurium (Te), barium (Ba), lanthanum (La), cerium (Ce), praseodymium (Pr), neodymium (Nd), samarium (Sm), europium (Eu), gadolinium (Gd), terbium (Tb), dysprosium (Dy), holmium (Ho), erbium (Er), thulium (Tm), ytterbium (Yb), lutetium (Lu), hafnium (Hf), tantalum (Ta), tungsten (W), rhenium (Re), osmium (Os), iridium (Ir), platinum (Pt), gold (Au), mercury (Hg), thallium (Tl), lead (Pb), bismuth (Bi), thorium (Th), and uranium (U), for reference year 2008.

## Materials and Methods

In this study, LCA is used as a tool to quantify and compare the cradle-to-gate environmental burdens of each element on the basis of a functional unit of 1 kg of each element at the factory gate. In addition, a bottom-up estimate of global impacts using year 2008 production data is provided. An LCA model following the ISO 14040 and 14044 standards [23], [24] is developed for each element using SimaPro8 software. LCI data used in this study are based on a combination of existing data sets [25], [27], [30], [31], [34]–[37] and an extensive literature research [39]–[65], [22], [66]–[102] as described in detail in Supporting Information S1. The modeled metals production system represents the global mining and metals sector in year 2008, using global production figures for each element, 2006 to 2010 price data for allocation (multi-output processes), and the most recent LCI data available from the literature. More details on the data set are provided in the sections below and Supporting Information S1. The comparative LCA model was constructed using the following steps: First, the 2008 production mix for each element is determined. For example, Ag is produced from Cu ores (17%), Pb ores (28%), Au-Ag ores (34%) and secondary sources (21%) [103], while all Cr is mined on its own (from chromite ore) but is used in different forms, i.e., as ferrochromium (74%), Cr metal (1%), sodium dichromate (17%) and chromite (8%) [25]. Results are then based on a production-weighted average over all production routes with a functional unit of 1 kg of each element at the factory gate. Data collection steps include: (a) Determining whether LCI data are available in existing databases; (b) If no LCI data exists, data are obtained either by direct data collection, or by integrating it with existing LCI data sets (e.g., Ge is a byproduct of Zn smelting and integrated into an existing smelting data set [25] based on the Ge content in the Zn concentrate and 2006–2010 price averages [104]) (see Supporting Information S1); (c) If LCI data exists, the data sets were used if representative of ≥80% of global production and the metal is produced on its own (bachelor). If the metal is co-produced, price data for allocation of environmental burdens were updated to 2006–2010 price averages given in United States Geological Survey (USGS) Mineral Commodity Summaries [104].

The resulting LCIs are linked to Ecoinvent 2.2 [27] data sets in the SimaPro8 LCA software. Impact assessment was performed for the categories of global warming potential (GWP) (IPCC 2007 GWP 100a v1.02 [105]), cumulative energy demand (CED) (Cumulative Energy Demand v1.08 [105]), terrestrial acidification, freshwater eutrophication, potential impacts to human health and ecosystem damage (World ReCiPe H/H Midpoint/Endpoint v1.08 [106]), and human toxicity (USETox v1.02 with recommended and interim characterization factors [107]). Monte Carlo simulation is used to assess the uncertainty associated with the results of the environmental assessment taking into account uncertainties of the LCI data (see Supporting Information S1).

### Metals Dataset

**Figure 1:**
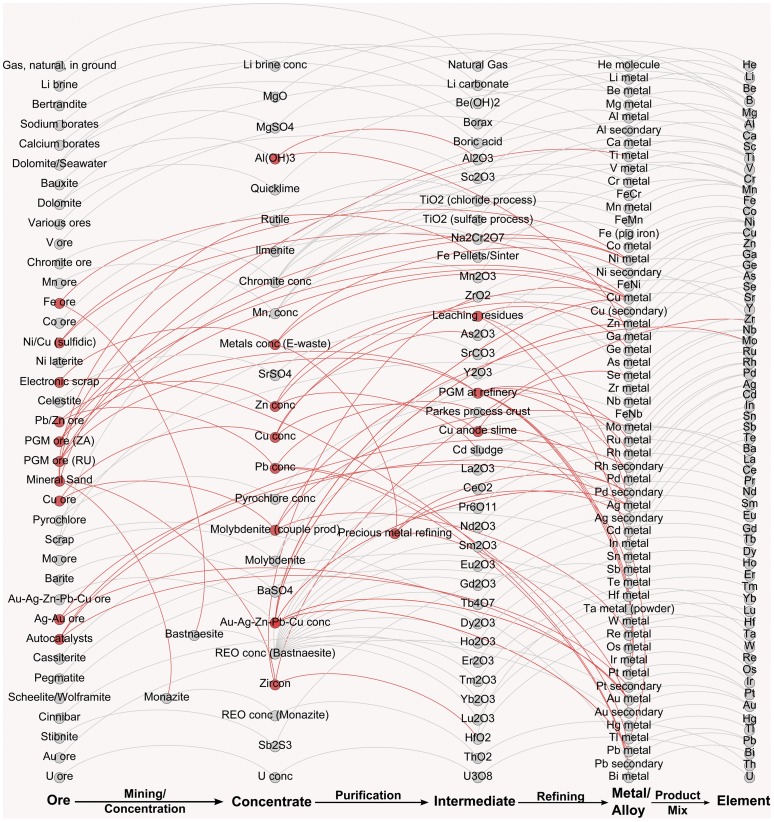
Diagram of the cradle-to-gate production of the minerals and metals (starting with ores from the left) analyzed in this study, in order of atomic number. Nodes representing mineral and metal products and intermediates, and the edges (arrows) indicating the physical transformation of metals via industrial processes from one chemical form (material) into another. Nodes and edges in red color represent joint production and illustrate the interconnectedness of the metals production system. Although not a metal, helium is includes in the assessment as it is sometimes regarded as a critical element required for the cooling of nuclear power plants. The figure was created using Gephi v0.8.2 [113].

The metal life cycles examined are pictured in Figure 1 for the 64 elements, covering virtually all of the metals used in modern products, from infrastructure applications to emerging technologies.

Starting from the left in Figure 1, our assessment includes the physical transformation of metal ores into mineral concentrates (mining and concentration), further transformation into mineral products and intermediates (purification), and subsequent conversion into the final metal or alloy (refining). Graph nodes represent the chemical form in which an element is present during each life cycle stage (i.e., ore, concentrate, intermediate, metal), while edges connecting two nodes indicate the physical transformation taking place as metals move through subsequent production steps. Since each of the 64 elements may be used in various chemical forms (e.g., as metal, oxide, sulfide), the overall environmental burdens for each elements consists of the weighted average of each chemical form supplied globally in 2008 (e.g., globally about 79% of lithium is used as Li_2_CO_3_ and 21% as Li metal [40]). This is indicated with the edges titled “product mix” and the nodes termed “element” in Figure 1.

In our data set, several metal production routes are interlinked with each other and form an intricate network of industrial processes (highlighted in red color in Figure 1). For metals obtained in multi-output processes (joint production), it is necessary to divide the environmental impacts from the process and all upstream processes among all metal co-product(s). Assigning each metal co-production an appropriate proportion of environmental impacts can be done in multiple, standardized ways, for example by applying mass allocation or economic allocation [24] as specified in the ISO standards for LCA. Economic allocation is used in the metals industry and is carried out by multiplying the price of each product output in dollars per kilogram by its quantity. In this paper, the 2006 to 2010 moving average prices from USGS Mineral Commodity Summaries [104] are used to allocate all environmental burdens across the metals production system shown in Figure 1. A 5-year price average is used to control for price volatility that may occur in a single year. For intermediate products not traded on a market, it is often difficult to obtain robust price information. Following Classen et al (2009) [25], 10% of the average market price is used for intermediate products (representing a 10% profit margin) with further details provided in Supporting Information S1. The choice of allocation is further investigated in a sensitivity analysis (see the discussion section below). In a few cases, disaggregation of LCI data was not possible for each life cycle stage and total impacts for the whole production chain are therefore reported. The complete dataset appears in Table S38 in Supporting Information S1 with additional information for each element provided in sections 2 to 67 of Supporting Information S1.

## Results

From our analysis, we show results separately for the three main stages of (1) mining/concentration, (2) purification, and (3) refining. Environmental burdens are examined on the basis of one kilogram of each element at the factory gate as well as on the basis of their annual production in year 2008.

**Figure 2:**
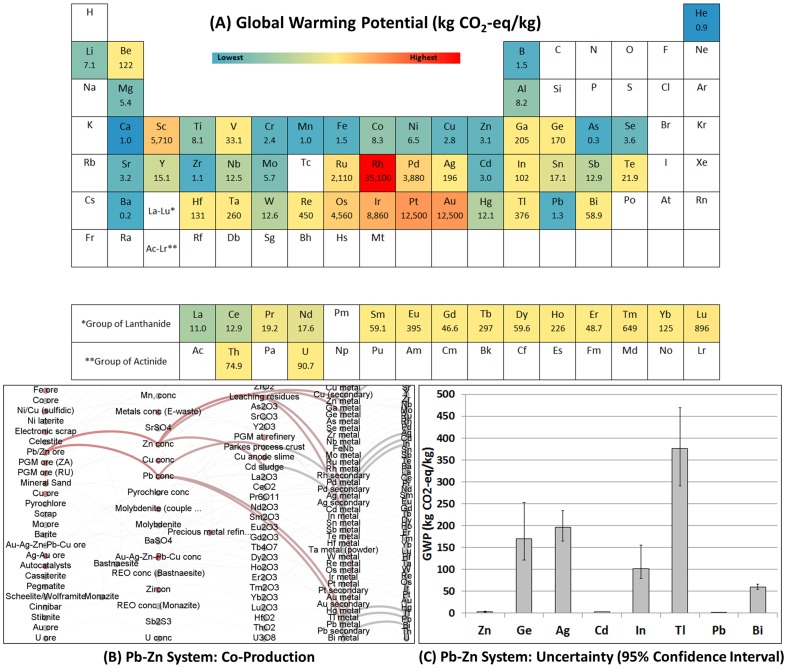
Periodic table of global warming potentials (GWPs). (A) The cradle-to-gate GWP per kilogram of each element (kg CO_2_-eq per kg) colored according to the color ramp above. GWPs shown are weighted by the 2008 supply mix for each element (see Table S38 in Supporting Information S1). (B) Illustration of the Pb-Zn system as an example of a joint production scheme (red color) from which Ge, Ag, Cd, In, Tl, and Bi are recovered as co- or by-products. (C) Uncertainty estimates (95% confidence interval) for the elements of the Pb-Zn system were derived using Monte-Carlo analysis.

**Figure 3:**
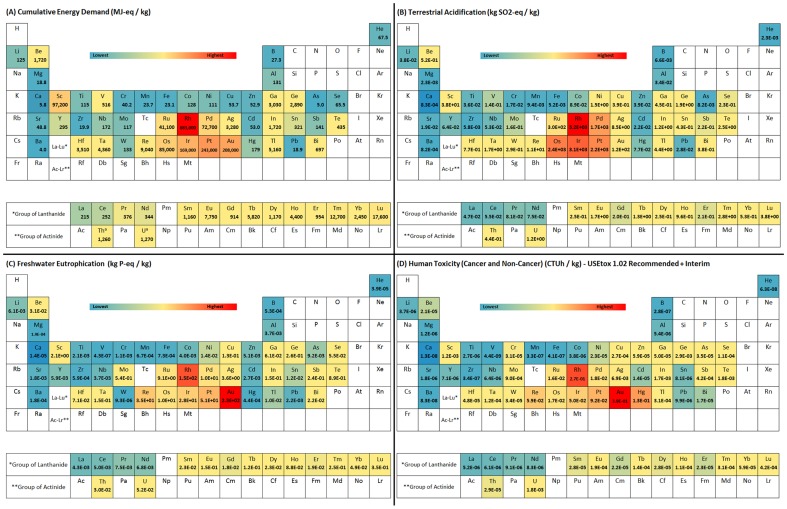
Periodic table of environmental impacts (colored according to the color ramp above). (A) Cradle-to-gate cumulative energy demand (CED) (MJ-eq/kg) per kilogram of each element. ^a^Cumulative energy demand for Th and U does not include non-renewable nuclear energy demand of U and Th in ground. (B) Cradle-to-gate terrestrial acidification (kg SO_2_-eq/kg). (C) Cradle-to-gate freshwater eutrophication (kg P-eq/kg). (D) Cradle-to-gate human toxicity (cancer and non-cancer) (CTUh/kg). Impacts to acidification and eutrophication were derived using the ReCiPe Midpoint method 1.08 H/H for the globe [106]. Human toxicity was calculated using the USETox v1.02. method with recommended and interim characterization factors [107].

The GWP per kilogram of each element (2008 supply mix) is given in Figure 2, together with an illustration of the interconnected lead-zinc production system and uncertainties associated with the results as an example of how the data set may be used to illustrate joint metals production. Figure 3 summarizes the impacts to CED, acidification, eutrophication, and human toxicity associated with each element.

Several features of the periodic tables are readily apparent. First, precious metals including platinum group elements (Ru, Rh, Pd, Os, Ir, Pt) and Au, display the highest environmental burdens on a per kilogram comparison, while many of the major industrial metals (e.g., Fe, Mn, Ti) are found at the lower end of the environmental impacts scale. For joint production systems (i.e., where one or more precious metals are co-produced with base metals such as copper or nickel) this is partly a result of the economic allocation chosen in this assessment, in which more of the environmental burdens is attributed to the expensive precious metals. Precious metals are those that are generally used in small quantities in products and industrial applications (e.g., catalysts, spark plugs, jewelry). Second, as expected from the use of mostly fossil energy carriers during metals production, there is a strong correlation between the cumulative energy use (Figure 3a) and GWP (Figure 2). Third, the impacts to acidification, eutrophication, and human toxicity (Figure 3b to d) reveal that for some elements, such as arsenic, copper, molybdenum, mercury and others, environmental burdens may not only be governed by the energy intensity and fuel mix of the metals production process, but may also be due to other factors including the disposal of sulfidic tailings or emissions of toxic or acidifying pollutants to air, soil, and water. Impacts other than GWP and CED could not be further investigated in this study due to the limited availability of LCI data for many of the metals investigated and models that describe, for instance, how mine tailings behave in the environment after disposal [108], and future research is required to do so. While USEtox [107] is recommended as the latest model for modeling human toxicity in LCA [3], metals are included only with interim characterization factors with high uncertainties (several orders of magnitude). The results of Figure 3d should therefore be treated as a first indication of potential impacts to human toxicity. Fourth, the above results imply that a comparison of metals on the basis of their annual global production may yield useful information regarding their absolute impacts, and an investigation into allocation of the environmentally relevant flows of the whole metals production network may elucidate the methodological challenges of reporting a single environmental burden value for each element.

**Figure 4:**
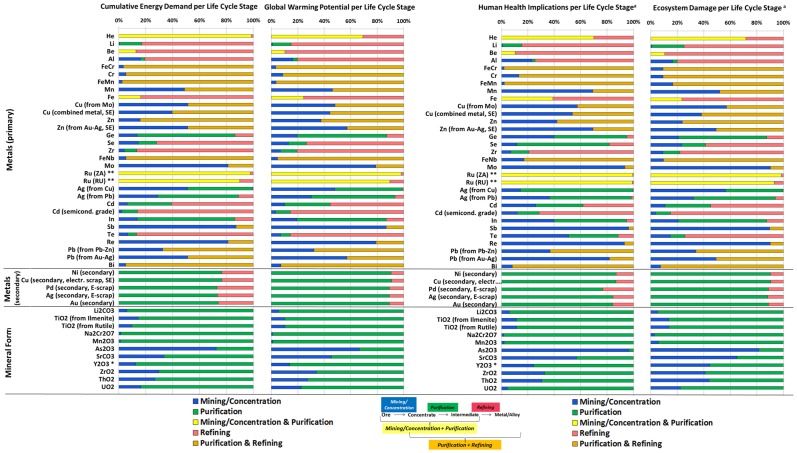
Relative environmental implications by life cycle stage. Only materials for which data for more than one life cycle stage was available are shown. Due to the aggregated nature of many of the data sets, in some instances the figure includes cumulative contributions from two life cycle stages (see legend). ^a^The human health implications (DALY/kg) and ecosystem damage (species.yr/kg) were calculated using the World ReCiPe Endpoint (H) impact assessment method v1.08 [106]. They represent potential damages prior to normalization and weighting. *The relative breakdown of the environmental burden of Y_2_O_3_ production is similar to other rare earths (i.e., La, Ce, Pr, Nd, Sm, Eu, Gd, Tb, Dy, Ho, Er, Tm, Yb, and Lu), which are therefore not shown in this figure. ** The breakdown of the environmental burden of Ru production is similar to other platinum group metals (i.e., Rh, Pd, Os, Ir, and Pt) from primary ore, which are therefore not shown in this figure. FeCr =  Ferrochromium. FeMn =  Ferromanganese. FeNb =  Ferroniobium. Elements in brackets indicate the host metal from which the metal is obtained as a co-product. SE =  Sweden.

While the periodic tables in Figure 2 and Figure 3 reveal information about the overall impact on a per kilogram basis, they do not break impacts down by life cycle stage, which is useful in analyzing opportunities to reduce system wide environmental pressures of the metals production process. A breakdown by life cycle stage for GWP, CED, human health, and ecosystem damage is illustrated in Figure 4.

As illustrated in Figure 4, for elements in their metallic form (either metals or alloys) the environmental burden are largely due to the purification (i.e., smelting) and refining stages required to obtain the final metal product (see Li, Be, Al, Ferrochromium (FeCr), Cr, Ferromanganese (FeMn), Fe, Cu (combined metal production, SE), Zn, Ge, Se, Zr, Ferroniobium (FeNb), Ru, Ag (from Pb), Cd, In, Te, Pb (from Pb-Zn), and Bi).

For Ge, In, and Ag, the purification stage contributes more to overall impacts than subsequent refining. This is because for metals the smelting process, producing intermediates such as anode slime or leaching residue, is included in the purification stage. For example, the purification stage of Ge and In includes the environmental burdens of Zn smelting, yielding a leaching residue from which both elements are recovered as co-products (together with Bi, and Tl) (see section 20 of Supporting Information S1). Similarly, Ag (from Pb) is recovered from Parkes process crust yielded during the smelting of primary Pb [25] with the environmental burdens of producing the intermediate accounted for in the purification stage.

For Ru (representative in terms of the contribution analysis for the platinum group metals (PGMs)), the aggregated nature of the data set did not allow the separation of the mining and concentration stage from other life cycle stages, which was therefore reported together with purification. The combined step includes the mining and concentration, leaching, hydrometallurgical treatment, and subsequent electrolytic precipitation of the PGM-containing fraction, yielding a dross that is then treated in a refinery [25].

A few metals display approximately similar impacts coming from both mining and concentration, and subsequent purification and refining (see Mn, Cu (from Mo), and Zn (from Au-Ag), and Pb (from Au-Ag). However, the figure also shows that for a number of metals (i.e., Mo, Ag (from Cu), Sb, Re, and Pb (from Au-Ag) the mining and concentration step may be a larger contributor to environmental pressures than subsequent purification and refining.

Secondary metals production (i.e., Ni, Cu, Pd, Ag, and Au from electronic scrap) is found to be dominated by the initial processing (smelting) of electronic scrap. It should be noted, however, that the estimates for secondary metals production shown in Figure 4 are based on inventory data from a single plant in Sweden only, as reported in Classen et al (2009) [25], and may not be representative of the global situation.

For elements in their mineral form (LiCO_3_, TiO_2_, Na_2_Cr_2_O_7_, Mn_2_O_3_, As_2_O_3_, SrCO_3_, Y_2_O_3_ [representative in terms of the contribution analysis for the rare earth oxides], ZrO_2_ ThO_2_, and UO_2)_, the environmental burdens are dominated by the purification stage.

A breakdown of human health implications and ecosystem damage on the right hand side of Figure 4 reveals that for some elements, the mining and concentration stage may increase in significance as toxicity related emissions during the mining process and, e.g., the disposal of overburden and tailing wastes are taken into account. As mentioned above, this analysis does not aim to provide a detailed assessment of toxicity-related implications of metals production and we recommend that further research may be carried out to refine the existing data set in this regard.

**Figure 5:**
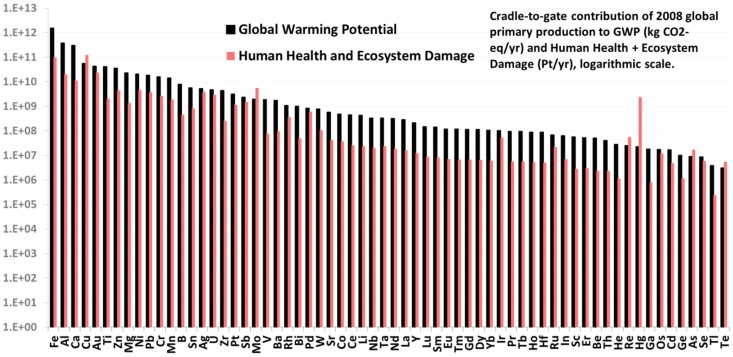
Global annual environmental implications of metals production in 2008. Per-kilogram impacts were multiplied with their respective production figures for year 2008 from USGS Mineral Yearbooks [53]. The cradle-to-gate human health and ecosystem damage (Pt/yr) were derived using the ReCiPe Endpoint method 1.08 H/H for the globe [106].

The per-kilogram impacts discussed above can be multiplied with their respective annual global production quantity in 2008 from USGS Mineral Yearbooks [53] to derive at an estimate of global greenhouse gas emissions and energy use of metals production. The results of this exercise are shown in Figure 5.

**Figure 6:**
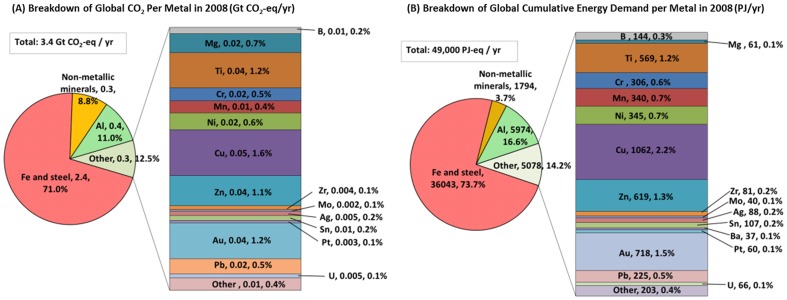
Breakdown of Global CO2 Emissions and Cumulative Energy Demand Per Metal in 2008.

Figure 5 illustrates the fact that, if taking into account the global annual production, the major industrial metals of Fe, Al, and Cu display the highest environmental impacts, while many of precious metals (with the exception of Au) are in the lower third of the impacts magnitude axis. A nice feature of this analysis is that global annual GWP and CED for each of the 63 metals investigated can be estimated with this bottom-up approach. This is done in Figure 6, and Table 1 compares our results with publicly available but highly aggregated, top-down data from the International Energy Agency (IEA).

**Table 1:** Comparison of the Breakdown of Global CO_2_ Emissions and Cumulative Energy Demand Per Metal to Other Studies.

Study	International Energy Agency (IEA)^a^	This study^b^	International Energy Agency (IEA)^a^	This study^b^
Unit	Gt CO_2_/yr	Gt CO_2_/yr	PJ/yr	PJ/yr
Fe and steel	2.5^c^	2.4^d^	23,446^j^	36,043^d^
Al	0.2^e^	0.4	3,894^k^	5,974
Other (non-ferrous metals)	0.2^e^	0.3	3,643^j^	5,078
Non-metallic minerals	0.1^f^^,^ g	0.3^i^	1,872^e^^,^ j	1,794^i^
Mining and quarrying	0.1^h^	-	2,219^j^	-
**Total**	**3.1**	**3.4**	**35,073**	**48,889**

a If not stated otherwise, the carbon dioxide (CO_2_) estimates include CO_2_ emissions from direct and indirect sources (i.e., upstream electricity production) [109]. Energy use is based on figures for final energy consumption [109], which refer to the energy supplied to the consumer, but do not include the transformation from primary energy carriers and feedstock energy.

b Derived by multiplying the per kg global warming potential (GWP) and cumulative energy demand (CED) for each element with their global annual production in year 2008. See Table S38 in Supporting Information S1 for more details.

c IEA (2008) [109] as reported in Allwood et al. (2009) [114]. Derived from Figure 16.6, page 483, in IEA (2008) [109].

d Based on the average of the ecoinvent 2.2. unit processes “Steel converter, unalloyed, at plant/RER U” and “Steel converter, low alloyed, at plant/RER U”, multiplied with USGS global raw steel production figures for 2008 [53].

e IEA (2007) [110] and IEA (2008) [109] as reported in Allwood et al. (2009) [114]. Assuming that aluminum accounts for 60% of CO_2_ emissions in the non-ferrous metals sector.

f Emissions from cement production are not counted, which equal 83% of total energy use and 94% of CO2 emissions in the production of non-metallic minerals (Chapter 16, page 490 of IEA (2008) [109]).

g Based on Figure 16.9, page 490, in IEA (2008) [109].

h Based on Table 16.4, page 481, in IEA (2008) [109]. Only direct industrial energy and process CO_2_ emissions included.

i Only limestone production.

j Based on Table 16.2, page 477, in IEA (2008) [109].

k Based on page 194 in IEA (2010) [10].

Table 1 shows that our bottom-up estimates are with 3.4 Gt CO_2_ per year approximately in line with the 3.1 Gt CO_2_ per year reported in IEA reports [10], [109]. This is reasonable because, with the exception of the mining and quarrying category, the IEA estimates include CO_2_ emissions from direct and indirect sources, in particular energy generation. In contrast, our estimates for CED of 49 PJ per year are 40% higher than the 35 PJ per year reported by the IEA, because the latter estimate only includes final energy use and does not capture primary energy use of the metals sector. An exception is the non-metallic minerals category, for which CED in this study is slightly lower than the IEA estimate of final energy use. The reason is that our CED estimate only includes limestone production, while the IEA number includes additional product categories such as clay brick and tile, building ceramics, and others [110].

Results from our study for the non-ferrous metals sector, consisting of a total of 61 metals (i.e., 63 metals minus Fe and steel and Al), are further broken down by metal type in Figure 6 and show that, with regard to global annual greenhouse gas emissions, Cu, Ti, Au, and Zn production account for the largest emissions within this sector. This information is occasionally provided for some of the major non-ferrous metals, e.g., Al, Cu, Cr, Mn, Ni [110], but is generally difficult to obtain for many of the minor metals such as Co, Ga, Re, and others.

**Figure 7:**
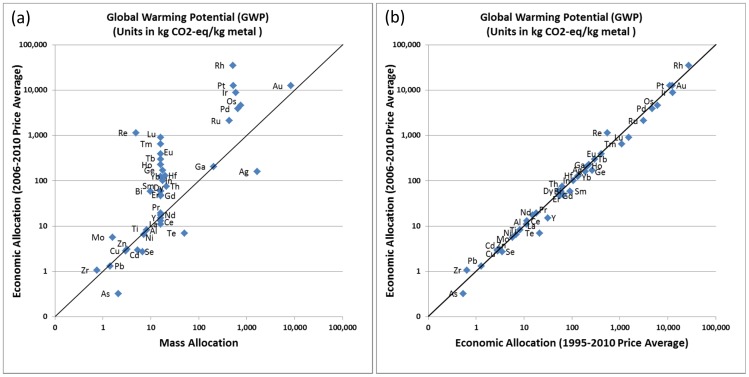
Choice of allocation rationale and implications on metals environmental burdens.

Of the 63 metals included in our data set, 42 are obtained as by- or co-products in multi-output processes (Figure 1), and environmental burdens are allocated using 2006 to 2010 moving average prices from USGS statistics [104]. Figure 7 compares impacts to GWP for each of the 42 metals using 2006 to 2010 price data for allocation with (a) mass allocation (Figure 7a), and (b) price-based allocation using 1995 to 2010 price averages (Figure 7b). The diagonal line indicates where results from mass and economic allocation are equal.

The wider spread on the y- than on the x-axis in Figure 7a shows that economic allocation results in more of the environmental burden being allocated, e.g., to the platinum group metals (Rh, Pt, Ir, Os, Pd, Ru), some rare earth elements (Lu, Tm, Eu, Tb, Ho, Sm, Er, Gd), Re, Ge, Hf, and In when compared to mass allocation. These elements are present in small quantities in the ores or intermediate products from which they are recovered and have high market prices (see, e.g., Table S25 in Supporting Information S1 for the rare earth elements and Table S30 in Supporting Information S1 for the platinum group metals). As a result, more of the environmental burdens are attributed to these metals using economic allocation than if mass allocation is applied. This can result in up to two orders of magnitude difference in the impact reported (y-axis in Figure 7a). The reason that mass allocation results in similar GWPs for the rare earth elements and the platinum group metals on a per kilogram basis (Figure 7a) is because in both cases the environmental impacts are derived from single inventory data sets, using mass allocation based on the elemental distribution in the metal concentrate. On the other hand, mass allocation results in higher GWP associated with Ag, Te, Se, Cd, and As. For example, in cases where Ag is obtained together with Au (about 27% ot total Ag production) more of the environmental burden is attributed to Ag if mass allocation is used because of its larger production volume, while economic allocation places more of the upstream burden on Au. Both Se and Te are obtained from anode slime during copper/silver production with more of the impact allocated to Ag (and less to Se and Te) taking into account price (see Table S12 to S14 in Supporting Information S1). As and Cd are obtained as low-valued by-products from copper and zinc production respectively with less of the environmental burden allocated to the elements taking into account price (see Table S9 in Supporting Information S1 for As, and Table S8b in Supporting Information S1 for Cd). Finally, Figure 7b indicates that results of our model are stable to price changes over time which was tested by comparing our data set using a 5-year price average (2006 to 2010) with average prices over a 15-year period (1995 to 2010).

## Discussion

Metals are important to maintain the materials base of modern technology and are expected to be of increasing importance in the transition toward sustainable technologies. Because of their widespread use in modern life, it is important to understand their life cycle wide environmental implications to make informed design choices and avoid shifting of environmental burdens. Quantifying the environmental burdens of metals production also gives a more systemic basis for evaluating benefits of material efficiency. This review of existing metals inventory data and collection of new data for several elements (discussed in Supporting Information S1) provides a detailed comparison of the cradle-to-gate environmental burdens of all of the metals used in modern technologies in their major use forms. This dataset can be used to help to inform product designers and decision makers in making environmentally preferred choices when considering alternative elements for use in new products, or when looking into metals substitution options within existing applications.

Perhaps the most obvious but highly significant result from this study is that the modern metals production system is a highly interconnected system with many of the metals being derived from multiple ores and as co-products with other metals along the stages of mining, purification, and refining (Figure 1). Because of this, it is difficult to treat each metal as an independent product and report environmental burdens independently of each other. By unifying the existing metals data, co-production issues can be better illuminated and sensitivity analysis, e.g., regarding different ways of allocating environmental burdens can be made, or assumptions regarding ore grades, recovery efficiencies, and different power mixes in the background system carried out in the future.

Our work showed that for the majority of elements in their metallic form, the cradle-to-gate environmental burdens are largely a result of the purification and refining stages (Figure 4). This trend may be partially altered in the future, given the fact that mines may gradually shift to lower grade ores which would increase the energy intensity of the mining and beneficiation process [11]–[13]. On the global level in 2008, the primary energy use of the metals and mining sector is estimated at 49 PJ per year (Table 1), which equals about 9.5% of global primary energy demand [111]. While on a per kilogram basis, the major industrial metals (e.g., Fe, Al) are found at the lower end of the environmental impacts scale (Figure 2 and Figure 3), they contribute significantly to global annual energy use and CO_2_ emissions simply because of their large annual production (Figure 6 and Table 1). The iron and steel sector alone is responsible for about 74% of primary energy demand within the mining and metals sector. Within the category of non-ferrous metals, consisting of 61 elements, our data set allows a breakdown by each metal; information useful when concerned with optimizing non-ferrous metals systems and life cycles from a point of view of environmental impacts. Considering such issues of scale and co-production information with each metal is important to acquire a better picture of the overall effectiveness and soundness of improvement and substitution options to overall environmental burdens [3].

A sustainable metals management system has to consider many aspects of a metals life cycle and include the environmental, social, and economic spheres of sustainability. One of the limitations of this study is the cradle-to-gate focus, thereby not considering use and end-of-life stages, which are particularly important for metals, being durable and readily recyclable. Several impact categories of immediate interest for metals systems (e.g., toxicity, abiotic resource depletion, and land use) [112], could not (or only partially) be examined in this study, and should be considered in the future as more site-specific inventory data become available and LCA methods for modeling impacts to human health become more sophisticated. Our data set is based on the best available information from the public literature and, given the breadth of the assessment, naturally includes certain data gaps and limitations. However, we hope that by providing detailed information with each element (Supporting Information S1) this dataset can be built upon and further improved in the future. This type of information will be of increasing importance, given the fact that the environmental burdens of metals are likely to become more visible in the future due to the steadily increasing demand [3].

## Supporting Information

Supporting Information S1 **This document contains further details on the LCA methodology and the data sources and assumptions used for the metals LCIs, along with several tables of the foreground LCIs.**
(DOCX)
