# Supplementary material for: Life Cycle Assessment of Metals: A Scientific Synthesis
Source: PLoS One. 2014 Jul 7;9(7):e101298. doi: 10.1371/journal.pone.0101298 (PMC4085040; doi:10.1371/journal.pone.0101298)
Supplement: Supporting Information S1 — This document contains further details on the LCA methodology and the data sources and assumptions used for the metals LCIs, along with several tables of the foreground LCIs. (DOCX) [file pone.0101298.s001.docx]

Life Cycle Assessment of Metals: A Scientific Synthesis

-SUPPORTING INFORMATION-

*Philip Nuss^a,*^ and Matthew J. Eckelman^b^*

^a^Center for Industrial Ecology, School of Forestry and Environmental Studies, Yale University, New Haven, Connecticut, United States of America

^b^Department of Civil and Environmental Engineering, Northeastern University, Boston, Massachusetts, United States of America

*Corresponding author phone: 203 432 0343; fax 203 432 5556; email: philip@nuss.me

**Abstract**

This document contains further details on the LCA methodology and the data sources and assumptions used for the metals life cycle inventories (LCIs), along with several tables of the foreground LCIs.

**Key words**

Life Cycle Inventory (LCI) Analysis, Metals Production System, Energy Use, CO_2_ Emissions, Coupled Production, Allocation, Sustainability

Contents

[**Abstract** 1](#_Toc390174081)

[**1.** **Notes** 7](#_Toc390174082)

[**2.** **Helium (He, Z=2)** 7](#_Toc390174083)

[**3.** **Lithium (Li, Z=3)** 8](#_Toc390174084)

[**4.** **Beryllium (Be, Z=4)** 8](#_Toc390174085)

[**5.** **Boron (B, Z=5)** 12](#_Toc390174086)

[**6.** **Magnesium (Mg, Z=12)** 12](#_Toc390174087)

[**7.** **Aluminum (Al, Z=13)** 13](#_Toc390174088)

[**8.** **Calcium (Ca, Z=20)** 13](#_Toc390174089)

[**9.** **Scandium (Sc, Z=21)** 15](#_Toc390174090)

[**10.** **Titanium (Ti, Z=22)** 16](#_Toc390174091)

[**11.** **Vanadium (V, Z=23)** 16](#_Toc390174092)

[**12.** **Chromium (Cr, Z=24)** 16](#_Toc390174093)

[**13.** **Manganese (Mn, Z=25)** 17](#_Toc390174094)

[**14.** **Iron (Fe, Z=26)** 17](#_Toc390174095)

[**15.** **Cobalt (Co, Z=27)** 17](#_Toc390174096)

[**16.** **Nickel (Ni, Z=28)** 17](#_Toc390174097)

[**17.** **Copper (Cu, Z=29)** 17](#_Toc390174098)

[**18.** **Zinc (Zn, Z=30)** 18](#_Toc390174099)

[**19.** **Gallium (Ga, Z=31)** 19](#_Toc390174100)

[**20.** **Germanium (Ge, Z=32)** 19](#_Toc390174101)

[**21.** **Arsenic (As, Z=33)** 22](#_Toc390174102)

[**22.** **Selenium (Se, Z=34)** 24](#_Toc390174103)

[**23.** **Strontium (Sr, Z=38)** 28](#_Toc390174104)

[**24.** **Yttrium (Y, Z=39)** 31](#_Toc390174105)

[**25.** **Zirconium (Zr, Z=40)** 31](#_Toc390174106)

[**26.** **Niobium (Nb, Z=41)** 34](#_Toc390174107)

[**27.** **Molybdenum (Mo, Z=42)** 37](#_Toc390174108)

[**28.** **Ruthenium (Ru, Z=44)** 37](#_Toc390174109)

[**29.** **Rhodium (Rh, Z=45)** 37](#_Toc390174110)

[**30.** **Palladium (Pd, Z=46)** 37](#_Toc390174111)

[**31.** **Silver (Ag, Z=47)** 37](#_Toc390174112)

[**32.** **Cadmium (Cd, Z=48)** 38](#_Toc390174113)

[**33.** **Indium (In, Z=49)** 38](#_Toc390174114)

[**34.** **Tin (Sn, Z=50)** 38](#_Toc390174115)

[**35.** **Antimony (Sb, Z=51)** 38](#_Toc390174116)

[**36.** **Tellurium (Te, Z=52)** 38](#_Toc390174117)

[**37.** **Barium (Ba, Z=56)** 38](#_Toc390174118)

[**38.** **Lanthanum (La, Z=57)** 41](#_Toc390174119)

[**39.** **Cerium (Ce, Z=58)** 42](#_Toc390174120)

[**40.** **Praseodymium (Pr, Z=59)** 42](#_Toc390174121)

[**41.** **Neodymium (Nd, Z=60)** 42](#_Toc390174122)

[**42.** **Samarium (Sm, Z=62)** 43](#_Toc390174123)

[**44.** **Europium (Eu, Z=63)** 43](#_Toc390174124)

[**45.** **Gadolinium (Gd, Z=64)** 43](#_Toc390174125)

[**46.** **Terbium (Tb, Z=65)** 43](#_Toc390174126)

[**47.** **Dysprosium (Dy, Z=66)** 43](#_Toc390174127)

[**48.** **Holmium (Ho, Z=67)** 43](#_Toc390174128)

[**49.** **Erbium (Er, Z=68)** 43](#_Toc390174129)

[**50.** **Thulium (Tm, Z=69)** 43](#_Toc390174130)

[**52.** **Ytterbium (Yb, Z=70)** 43](#_Toc390174131)

[**53.** **Lutetium (Lu, Z=71)** 43](#_Toc390174132)

[**54.** **Hafnium (Hf, Z=72)** 43](#_Toc390174133)

[**55.** **Tantalum (Ta, Z=73)** 44](#_Toc390174134)

[**56.** **Tungsten (W, Z=74)** 44](#_Toc390174135)

[**57.** **Rhenium (Re, Z=75)** 46](#_Toc390174136)

[**58.** **Osmium (Os, Z=76)** 46](#_Toc390174137)

[**59.** **Iridium (Ir, Z=77)** 46](#_Toc390174138)

[**60.** **Platinum (Pt, Z=78)** 46](#_Toc390174139)

[**61.** **Gold (Au, Z=79)** 49](#_Toc390174140)

[**62.** **Mercury (Hg, Z=80)** 49](#_Toc390174141)

[**63.** **Thallium (Tl, Z=81)** 50](#_Toc390174142)

[**64.** **Lead (Pb, Z=82)** 51](#_Toc390174143)

[**65.** **Bismuth (Bi, Z=83)** 51](#_Toc390174144)

[**66.** **Thorium (Th, Z=90)** 52](#_Toc390174145)

[**67.** **Uranium (U, Z=92)** 57](#_Toc390174146)

[**68.** **Summary** 57](#_Toc390174147)

[**69.** **References** 71](#_Toc390174148)

Table of Figures

[Figure S1 Steps of metals life cycle inventory (LCI) data collection. 6](#_Toc390171955)

[Figure S2 Process flow diagram showing the beryllium production process 8](#_Toc390171956)

[Figure S3 Illustration of processes and flows from the mining of zinc-lead deposits 19](#_Toc390171957)

[Figure S4 Copper multioutput system modeled in this study. 24](#_Toc390171958)

[Figure S5 Overview of zirconium sponge production 31](#_Toc390171959)

Table of Tables

[Table S1 Global lithium use categories and chemical forms into use. 8](#_Toc390171964)

[Table S2 Life Cycle Inventory for beryllium hydroxide production 10](#_Toc390171965)

[Table S3 Beryllium metal production in semi-finished products according to [15]. 11](#_Toc390171966)

[Table S4 Global borate end-uses in year 2008. 12](#_Toc390171967)

[Table S5 Calcium metal production in year 2005. 14](#_Toc390171968)

[Table S6 Allocation of environmental burdens between copper- and molybdenum concentrate. 18](#_Toc390171969)

[Table S7 Copper output by source metal in year 2005 18](#_Toc390171970)

[Table S8 Relevant parameters to determine the allocation split for (a) lead and zinc mining and concentration, (b) zinc smelting and production, and (c) lead and silver-containing crust 21](#_Toc390171971)

[Table S9 Allocation between blister copper and arsenic trioxide. 22](#_Toc390171972)

[Table S10 Life cycle inventory showing process names chosen in the Ecoinvent database for 1 metric ton of arsenic production as byproduct from copper 23](#_Toc390171973)

[Table S11 Production factors for the byproducts silver, tellurium, and selenium, in the anode slime from copper production 26](#_Toc390171974)

[Table S12 Revenue allocation between cathode copper and anode slime. 26](#_Toc390171975)

[Table S13 Updated allocation percentages of anode slime recovery towards silver and CuTe cementate. 27](#_Toc390171976)

[Table S14 Inclusion of the upstream environmental burdens of anode slime production in the selenium life cycle. 27](#_Toc390171977)

[Table S15 End-uses of strontium in the United States in 2004 28](#_Toc390171978)

[Table S16 Life Cycle Inventory for celestite mining and beneficiation. 29](#_Toc390171979)

[Table S17 Life Cycle Inventory for the production of strontium carbonate 31](#_Toc390171980)

[Table S18 Allocation factors applied to metal production from zirconium oxide. 33](#_Toc390171981)

[Table S19 Life Cycle Inventory for the production of metallic zirconium and hafnium. 33](#_Toc390171982)

[Table S20 Life cycle inventory showing process names chosen in the Ecoinvent database for 1 metric ton of niobium oxide production 34](#_Toc390171983)

[Table S21 Raw material needs for aluminothermic provision of 1 kg ferroniobium. 35](#_Toc390171984)

[Table S22 Energy use for Niobium production in the early 1990s 36](#_Toc390171985)

[Table S23 Niobium production statistics 36](#_Toc390171986)

[Table S24 Life Cycle Inventory for the production of calcium carbonate. Based on stoichiometric relationships, assuming a yield of 80% 40](#_Toc390171987)

[Table S25 Calculation of the environmental impacts of rare earth oxide (REO) production 42](#_Toc390171988)

[Table S26 Life cycle inventory showing process names chosen in the Ecoinvent database for 1 metric ton of tungsten production 44](#_Toc390171989)

[Table S27 Allocation factors used for rhenium production as a by-product from molybdenum roasting. 46](#_Toc390171990)

[Table S28 Production from year 1970 to 1992 by platinum grade metal (PGM) 48](#_Toc390171991)

[Table S29 Ore distribution of platinum grade metals (PGMs) in South Africa and Russia based on various literature sources. 48](#_Toc390171992)

[Table S30 Reallocation of environmental burdens of the platinum grade metals (PGMs) 49](#_Toc390171993)

[Table S31 Life cycle inventory showing process names chosen in the Ecoinvent database for 1 metric ton of thallium production 50](#_Toc390171994)

[Table S32 Bismuth by-production based on Canadian sustainability report 52](#_Toc390171995)

[Table S33 Composition of monazite concentrates 53](#_Toc390171996)

[Table S34 Rare earth distribution in monazite from the three major mining locations 53](#_Toc390171997)

[Table S35 Product outputs from the processing of heavy mineral sands. 54](#_Toc390171998)

[Table S36 Life cycle inventory for LAMP plant producing rare earth oxides (REOs) and thorium oxide from monazite in Malaysia 56](#_Toc390171999)

[Table S37 Distribution of rare earths and thorium in the final mixture obtained from monazite and their respective allocation percentages using 2006-2010 price data. 57](#_Toc390172000)

[Table S38 Summary table of metal environmental implications 58](#_Toc390172001)

1. **Notes**

Life cycle inventory (LCI) data for the elements and their product mixes come from various sources, including LCI databases, scientific journal articles, technical reports, and personal communications. The sources and assumptions made are given in subsequent sections with the goal to provide transparent data so that the compiled and updated inventories can be further improved and updated. A summary of all metals and their major use forms is provided in Table S38 at the end of this manuscript. If no sufficient uncertainty data are reported with the data source, the probability distributions for each LCI parameter are based on a semi-quantitative approach [1,2]. Data collection steps are illustrated in Figure S1.

Figure S1 Steps of metals life cycle inventory (LCI) data collection.


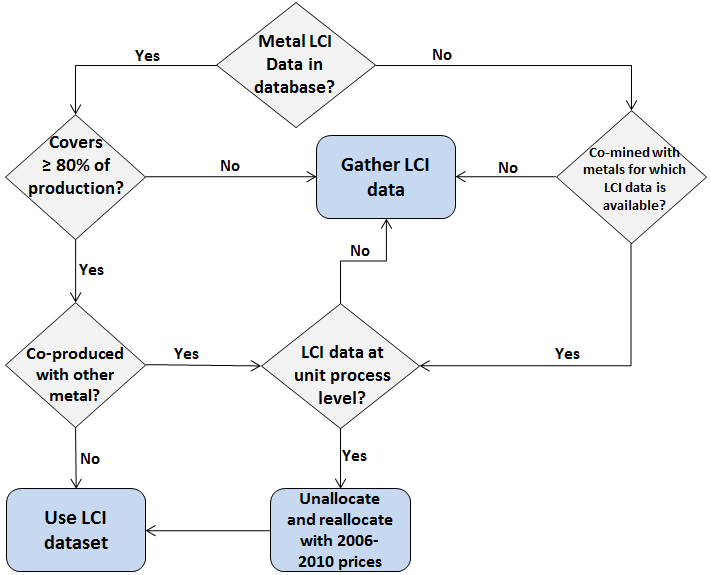


1. **Helium (He, Z=2)**

Helium is produced mostly by extraction from natural gas, in which it is present at a content of 0.005 to 8% [3]. The environmental burdens per kg helium are approximated using the Ecoinvent entry “Helium, at plant/GLO U” from the Ecoinvent database [4]. Allocation between helium, natural gas liquids, and sales gas from helium extraction are allocated based on the energy content per MJ natural gas as done in Sutter (2007) [5]. Helium is included in the assessment as it is regarded as a critical element required for the cooling of nuclear power plants (Helium does not become radioactive during this process).

1. **Lithium (Li, Z=3)**

World production of lithium in 2009 was from brine (66%) and hard rock minerals (34%) [6]. The lithium environmental burdens are derived using existing ecoinvent [4] data of lithium metal and lithium carbonate (see Table S38) – both of which are based on lithium from brine. Lithium carbonate serves as the starting material for a variety of lithium-containing products such as lithium hydroxide, lithium manganese oxide, and lithium chloride. Table S1 illustrates global lithium end uses according to Gruber et al (2011) [7].

Table S1 Global lithium use categories and chemical forms into use.

| **Lithium uses**  [7] | **Chemical form** | **Percentage in 2008**  [7] |
| --- | --- | --- |
| Batteries | Various (e.g., lithium manganese oxide or lithium iron phosphate as cathode; graphite, lithium titanate, hard carbon as anode)  (from lithium carbonate as starting material) | 25% |
| Frits & Glass | Lithium carbonate | 18% |
| Lubricants | Lithium hydroxide  (from lithium carbonate as starting material) | 12% |
| Air conditioners | Lithium bromide, lithium chromate, lithium chloride  (from lithium carbonate as starting material) | 6% |
| Aluminum | Lithium metal | 4% |
| Other | various (assuming 50% metal, 50% lithium carbonate) | 35% |

1. **Beryllium (Be, Z=4)**

In 2008 the United States accounted for approximately 89% of the freshly mined world beryllium production, followed by China 10%, Madagascar, Mozambique, and Portugal (all together 1%) [8]. In the United States, virtually all beryllium is produced solely from bertrandite ore (Be_4_Si_2_O_7_(OH)_2_), in Kazakkhstan from an inventory of a similar ore that was historically mined by the Soviet Union, while beryl is the primary source of the element elsewhere (Be_3_Al_2_(SiO_3_)_6_) [9]. The processing of bertrandite ores differs from the processing of beryl ores, as the former cannot be economically refined via conventional methods and a new process, named the SX-Carbonate process, was therefore developed [10]. In the United States, bertrandite ores are processed into beryllium hydroxide, beryllium alloys, and beryllium oxide in ceramics by Materion Corporation (formerly Brush Wellman). The following life cycle inventory discusses the production of bertrandite ore into beryllium hydroxide (the starting material for subsequent beryllium oxide and beryllium alloys) and subsequent beryllium metal, as it represents the dominant route of production in year 2008. A process flow diagram is shown in Figure S2.

Figure S2 Process flow diagram showing the beryllium production process. Only processes within the system boundary are accounted for (i.e., beryllium hydroxide production and the production of semi-finished products consisting of beryllium metal (e.g., metal shapes, tubes). The environmental burdens of beryllium oxide (used in ceramics) and beryllium alloys (copper beryllium alloys) production are excluded because both are produced from beryllium hydroxide (i.e., Be(OH_2_) represents the principal chemical form from which all subsequent materials are obtained).


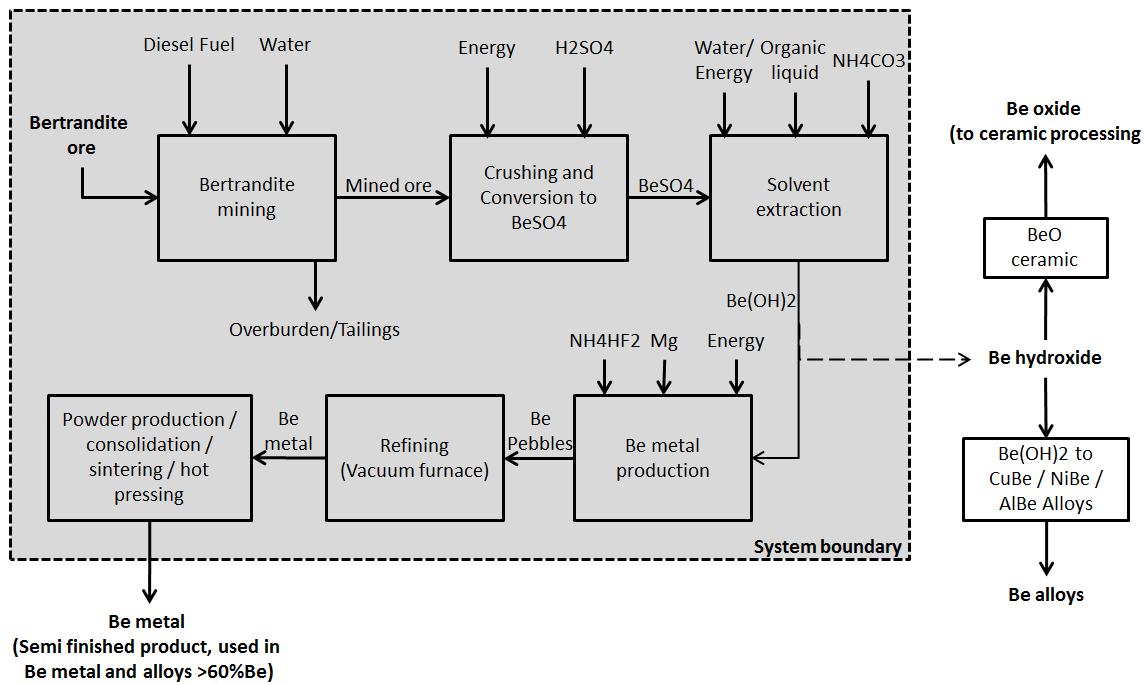


The majority of bertrandite ore in the United States contains 0.3-1.5% beryllium and is mined in Utah [11]. After an ore body is identified, overburden is removed during winter and spring and the ore mined using a self-loading scraper [9]. After mining, the overburden and tailings are replaced in the mine from where they were previously removed [11]. The recovery rate of beryllium from the ore is 87% [12]. Bertrandite ore is stockpiled on-site and then transported by truck to the beryllium processing facility near Delta (80 km transport distance) [9]. Bertrandite is dissolved using sulfuric acid and the insoluble residue (SiO_2_, and aluminum precipitating as alum) is removed [10]. The sulfate solution undergoes stepwise extraction using an organic solvent (i.e., ammonium di-2-ethylhexyl phosphate/di-2-ethylhexyl phosphoric acid/kerosene (DAP)) with beryllium as well as other metal ions (aluminum and iron) entering the organic phase [10]. Beryllium is subsequently removed from the organic phase using an ammonium carbonate solution and the organic solvent returned to the process (hence, not accounted for in LCI). Heat treatment separates the beryllium for other metal ions and a beryllium hydroxide is obtained as final product. Table S2 provides the compiled life cycle inventory based on data from [9,13,14].

Table S2 Life Cycle Inventory for beryllium hydroxide production

| **Input** | **Unit** | **Amount** | **Distribution** | **StDv95%** | **Notes** |
| --- | --- | --- | --- | --- | --- |
| Bertrandite Ore | kg | 435 | Lognormal | 1.24 | (3,2,1,3,1,5) [14] |
| Ammonia (NH3) | kg | 14.9 | Lognormal | 1.24 | (3,2,1,3,1,5) [14] |
| Sulfuric acid (H2SO4) | kg | 7.4 | Lognormal | 1.24 | (3,2,1,3,1,5) [14] |
| Water | m3 | 3.7 | Lognormal | 1.24 | (3,2,1,3,1,5) [14] |
| Energy (Diesel Fuel) | MJ | 105 | Lognormal | 1.24 | (3,2,1,3,1,5) [14] |
| Natural Gas | MJ | 76 | Lognormal | 1.24 | (3,2,1,3,1,5) Heat input to process [14] |
| Electricity | MJ | 76 | Lognormal | 1.24 | (3,2,1,3,1,5) U.S. power grid [14] |
| Truck | tkm | 0.08 | Lognormal | 2.06 | (3,2,1,3,1,5) 80 km transport distance from mine site to beryllium processing facility [9] |
| Conveyor belt, at plant | m | 3.31E-05 | Lognormal | 3.35 | (2,4,4,5,4,5) same values as in copper exploitation (7.6E-5m/t ore) |
| Non-ferrous metal mine, surface | unit | 2.18E-08 | Lognormal | 3.35 | (2,4,4,5,4,5) Generic Ecoinvent process, 20,000Mt assumed during 50 year lifetime [13] |
| Transformation from unknown | m2 | 3.74E-03 | Lognormal | 2.35 | (2,4,4,5,4,5) Based on non-ferrous metals processing estimate from ) |
| Transformation, to mineral extraction site | m2 | 3.74E-03 | Lognormal | 2.35 | (2,4,4,5,4,5) Based on non-ferrous metals processing estimate from [13] |
| Transformation, to unknown | m2 | 3.74E-03 | Lognormal | 2.35 | (2,4,4,5,4,5) Based on non-ferrous metals processing estimate from [13] |
| Occupation, mineral extraction site | m2a | 1.65E-01 | Lognormal | 1.65 | (2,4,4,5,4,5) Based on non-ferrous metals processing estimate from [13] |
| **Output** |  |  |  |  |  |
| Be(OH)2 | kg | 4.77 | - | - | 1 kg Be contained in Be(OH)2 [14] |
| Tailings | kg | 228 | - | - | returned to mine and therefore not accounted for [14] |
| Overburden | kg | 206 | - | - | returned to mine and therefore not accounted for [14] |

The transformation of beryllium hydroxide into beryllium metal is carried out by generating beryllium fluoride which is subsequently reduced using magnesium [10,11]. For this, the beryllium hydroxide is first dissolved in ammonium bifluoride solution and the resulting precipitate heated to produce molten beryllium fluoride. The beryllium fluoride is then mixed in a crucible with molten magnesium and a mixture of beryllium pebbles and magnesium fluoride obtained [11]. External energy input in the form of heat (natural gas) is required (endothermic reaction). The pebbles are further refined by melting in a vacuum furnace. Beryllium metal is then converted into solid shapes, using powder metallurgy, sintering, and hot pressing [11,14]. Due to the aggregated nature of the inventory data set, it was not possible to disaggregate the production of beryllium metal from subsequent production of semi-finished products. Therefore, the energy requirements used in this assessment include the additional semi-finished products production step.

Table S3 Beryllium metal production in semi-finished products according to [14].

| **Input** | **Unit** | **Amount** | **Distribution** | **StDv95%** | **Notes** |
| --- | --- | --- | --- | --- | --- |
| BeOH | kg | 4.77 | Lognormal | 1.24 | (3,2,1,3,1,5) |
| Natural Gas | MJ | 1845 | Lognormal | 1.24 | (3,2,1,3,1,5) |
| Electricity | MJ | 1845 | Lognormal | 1.24 | (3,2,1,3,1,5) U.S. power grid |
| Ammonia (NH3) | kg | 4.4 | Lognormal | 1.24 | (3,2,1,3,1,5) |
| Hydrofluoric acid (HF) | kg | 4.9 | Lognormal | 1.24 | (3,2,1,3,1,5) |
| Sodium hydroxide (NaOH) | kg | 14.2 | Lognormal | 1.24 | (3,2,1,3,1,5) |
| Water | m3 | 3.7 | Lognormal | 1.24 | (3,2,1,3,1,5) |
| Magnesium | kg | 3 | Lognormal | 1.24 | (3,2,1,3,1,5) |
| Chemical plant, organics | unit | 8.00E-10 | Lognormal | 3.06 | (3,2,1,3,1,5) Facilities approximated with "chemical plant , organic", which has an output of 50,000 tons per year (25 year lifetime). |
| **Output** |  |  |  |  |  |
| Be metal | kg | 1 | - | - | - |
| Be, to air^a^ | kg | 4.54E-04 | Lognormal | 5.07 | (3,2,1,3,1,5) |
| Nitrogen oxides, to air^a^ | kg | 0.404 | Lognormal | 1.58 | (3,2,1,3,1,5) |
| Particulates, <10um, to air^a^ | kg | 0.071 | Lognormal | 3.06 | (3,2,1,3,1,5) |
| Solid waste, to landfill | kg | 1.4 | Lognormal | 1.24 | (3,2,1,3,1,5) unspecified, The Ecoinvent unit process "Process-specific burdens, residual material landfill/CH U is used. |

^a^ Conservative estimate as it represents the cumulative emissions estimate per kg beryllium produced at all operations (i.e., includes alloying and production of semi-finished shapes sold to end-users).

Of beryllium consumed globally in year 2011, roughly 12% is in its metallic form as beryllium metal and in alloys (>60% Be), while the remaining 88% are based upon the beryllium hydroxide form that is subsequently being converted into copper beryllium alloys (86% out of the 87%) and beryllium oxide used in ceramics (1% out of the 88%) [11]. Hence, the environmental impact score is based upon a weighted average of 12% beryllium metal and 88% beryllium hydroxide.

1. **Boron (B, Z=5)**

Globally, Turkey and the United States are the main producers of boron minerals [15]. While in Turkey, boric acid is primarily obtained from calcium borate (i.e., colemanite mineral), in the United States it comes from sodium borate minerals [16]. The majority of borates are used in the manufacture of glass and ceramics, with smaller amounts going into detergents, fertilizer and other uses (Table S4).

For this assessment, the existing Ecoinvent entries shown in the table below are used, assuming that borax is generally used in glass, ceramics, and fertilizer uses, while boric acid finds use in detergents and other uses. These model production in the United States and Turkey – the major producers of boron products.

Table S4 Global borate end-uses in year 2008.

| **End-use** | **Percentage into use (2008)** | **Chemical Form** | **Ecoinvent dataset used** |
| --- | --- | --- | --- |
| Glass | 61 | Borax | Borax, anhydrous, powder, at plant (GLO) |
| Ceramics | 14 | Borax | Borax, anhydrous, powder, at plant (GLO) |
| Detergent | 7 | Boric acid | Boric acid, anhydrous, powder, at plant (RER) |
| Fertilizer | 6 | Borax | Borax, anhydrous, powder, at plant (GLO) |
| Other | 12 | Boric acid |  |
| Total | 100 |  |  |
| Source | USGS (2008) [15] | Smith (2000) [16] | [17] |

Borax = Sodium borate. Boric acid = H_3_BO_3_.

1. **Magnesium (Mg, Z=12)**

The magnesium environmental burdens are derived using [4] data of magnesium metal, magnesium oxide, and magnesium sulfate (see Table S38). In 2008, global magnesium uses were comprised of the following: 83% refractories (as MgO), 6% water treatment and stack-gas scrubbing (as MgO or MgSO4 - here assumed to be 3% MgO and 3% MgSO4), 5% agriculture (as MgO or MgSO4 - here assumed to be 2.5% MgO and 2.5% MgSO4), and 6 % other (metal uses) [18]. Globally, magnesium is obtained from dolomite rock (47%), magnesite rock (18%), carnallite (13%), and brines from saline lakes and seawater (22%) [19]. In this study, magnesium production is modeled using the existing ecoinvent data sets for magnesium from seawater [13].

1. **Aluminum (Al, Z=13)**

The aluminum environmental burdens are derived using [4] data of primary aluminum, secondary aluminum (from old scrap), and secondary aluminum (from new scrap) (see Table S38).

1. **Calcium (Ca, Z=20)**

As part of the Yale criticality assessments, metallic calcium and calcium oxide were investigated as substitutes for other elements. This section briefly describes how a simple life cycle inventory for these was derived.

Calcium oxide (quicklime) is produced by the thermal decomposition of limestone following the chemical reaction below.

CaCO_3_ 🡪 CaO + CO_2_

Limestone consists primarily of calcium carbonate but generally also contains magnesium carbonate (2-5%) as secondary component [20]. Limestone is used in a wide variety of industrial applications, including construction, cement making, quicklime, agriculture, metal refining, and flue gas desulphurization [20]. For this assessment an existing life cycle inventory of quicklime production from the Ecoinvent database is used [21].

Environmental burdens were estimated based on reports of embodied energy of primary production. In its materials database, CES 2012 Selector software [22] reports an embodied energy content for metallic calcium of 201 MJ/kg (a range of 191 – 211 MJ/kg is given in the database). Metallic calcium is obtained via electrowinning and the aluminothermic process [23]. During electrolysis, calcium collects at the cathode and a typical metal purity of 98% can be achieved. During the aluminothermic process, calcium oxide (quicklime) is reduced to metallic calcium at temperatures above 2000ºC [24].

6 CaO + 2Al = 3CaO∙Al_2_O_3_ + 3Ca

33CaO + 14 Al = 12CaO∙7Al_2_O_3_ +21Ca

4CaO + 2Al = CaO∙Al_2_O_3_ + 3Ca

Metallic calcium production by country and process type in year 2005 is shown in Table S5.

Table S5 Calcium metal production in year 2005.

| **Country** | **Process type** | **Probable energy type** | **Production year 2005 (metric tons/year)** | **Source** |
| --- | --- | --- | --- | --- |
| China | Electrolysis | Electricity | 11,000 (46%) | [23] |
| Russia | Electrolysis | Electricity | 7,000 (29%) | [23] |
| USA | Aluminothermic | Coal/Natural Gas, Electricity during aluminum provision | 3,000 (13%) | [23] |
| Canada | Aluminothermic | Coal/Natural Gas, Electricity during aluminum provision | 1,500 (6%) | Not reported. Remaining production split equally between Canada and France. |
| France | Unknown (Electrolysis assumed) | Electricity | 1,500 (6%) | Not reported. Remaining production split equally between Canada and France. |
| Total | - | - | 24,000 (100%) | [23] |

Final energy use during the calcium production process is assumed to come solely from electricity. Assuming an average conversion efficiency of 35% for electricity the embodied energy of 201 MJ/kg [22] equals 19.5 kWh of final electricity demand.

Cradle-to-gate environmental burdens are then estimated by using a weighted average electricity input for the two major producing countries (China, Russia, USA, Canada, and France – see Table S5) using 2008 fuel shares from [25] and linking those to respective Ecoinvent electricity production processes in SimaPro. A triangular uncertainty distribution with the min and max values provided by Granta Design (2012) [22] is assumed.

In 2008, global production of quicklime equaled 307,000,000 metric tons (99.99%) [26]. This compares to roughly 32,000 metric tons of calcium metal produced annually (0.01%) [27].

1. **Scandium (Sc, Z=21)**

In 2008, global production of scandium oxide as by-product metal took place in China, Kazakhstan, Russia, and Ukraine [28]. Scandium is present in trace amounts in most rare earth minerals along with other rare earth elements [29]. However, the concentrations are generally very low. Scandium minerals with workable amounts of scandium include thortveitite, euxenite, and gadolinite. Scandium is also present in small quantities in ores of aluminum, cobalt, iron, molybdenum, nickel, phosphate, tantalum, tin, titanium, tungsten, uranium, zinc, and zirconium [30,31]. Scandium is recovered via hydrometallurgical processes (which include leaching, solvent extraction, and precipitation) which, due to the low contents in ores and slags, is a very energy intensive process [31]. The same source also states that besides primary production, about half of global scandium may originate from the stockpiles of Russia generated during the cold war [31]. Consumption of scandium in 2012 was estimated at less than 10 metric tons [32].

While a review paper of various production processes towards scandium is available [31], the exact contribution of each route to total production is unclear. Similarly, to our knowledge no publicly reported information on the environmentally relevant flows associated with each scandium production route is available. However, the Granta Design Eco Database reports the embodied energy of scandium (>99%) production to range between 80,064 and 88,458 MJ/kg [22]. In their database, embodied energy values are partly derived by looking at the correlation between embodied energy and materials prices averaged over eight years [33].

Due to a lack of better data the following assumptions are made to derive at an estimate of scandium environmental implications:

- The cumulative energy demand of producing 1 kg of scandium at the factory gate equals 84,261 MJ/kg [22]. The uncertainty is described by a triangular distribution with a minimum and maximum value of 80,064 and 88,458 MJ/kg, respectively.
- All energy inputs to the hydrometallurgical recovery of scandium as a by-product from other metals is in the form of electricity (extraction and refining) with an average of conversion efficiency of 35%.
- Production takes place at 50% in Russia [31] and the remaining 50% are equally split between China, Kazakhstan, and Ukraine [28] using the 2008 fuel shares from [25] and linking those to respective Ecoinvent electricity production processes in SimaPro8.
- The energy input is the main driver of environmental implications.

1. **Titanium (Ti, Z=22)**

The titanium environmental burdens are derived using data of titanium dioxide (sulfate process) and titanium dioxide (chloride process) [4], as well as titanium metal [34] (see Table S38). In 2011, about 47% of all titanium dioxide was produced from ilmenite with the remainder coming from rutile [35]. Titanium dioxide is obtained from mineral sand and the environmental burden allocated between ilmenite (54% TiO_2_), rutile (95% TiO_2_), zircon (50% ZiO_2_), and monazite (containing rare earths as well as thorium oxide), based on 2006-2010 price data as shown in Table S35.

1. **Vanadium (V, Z=23)**

The majority of global vanadium is derived as a by-product in processing iron, titanium, and uranium ores, as well as from residuals (ash, fume, coke) from petroleum production [36]. About 81% of vanadium produced annually is derived as a byproduct from vanadium slags and as primary vanadium from mining, while the remaining 19% come from secondary resources (i.e. residues, spent catalyst, uranium residue) [37].

Inventory data for metallic vanadium production is available from [34], based on estimates of energy use during mining, concentrating, melting and transport according to global production in 2000. According to this data source, vanadium metal production leads to a CED of 516 MJ-eq/kg, which is close to the embodied energy of 454 MJ-eq/kg given in [22]. Lognormal distributions are assigned to each inventory parameter using the pedigree matrix [1,2]. Inventory data on other primary vanadium production routes and secondary production was not available.

1. **Chromium (Cr, Z=24)**

The chromium environmental burdens are derived using data of ferrochromium, chromium metal, sodium dichromate, and chromite [4] (see Table S38).

1. **Manganese (Mn, Z=25)**

The manganese environmental burdens are derived using data of ferromanganese, manganese metal, manganese concentrate, and manganese oxide [4] (see Table S38).

1. **Iron (Fe, Z=26)**

The iron environmental burdens are derived using data of pig iron [4] (see Table S38).

1. **Cobalt (Co, Z=27)**

The cobalt environmental burdens are derived using data of cobalt, at plant GLO/U [4] (see Table S38).

1. **Nickel (Ni, Z=28)**

The nickel environmental burdens are derived using data of ferronickel, nickel metal (from sulfidic ores), nickel (from platinum group metal production, South Africa), nickel (from platinum group metal production, Russia), and secondary nickel (from electronic and electric scrap) [4] (see Table S38).

1. **Copper (Cu, Z=29)**

The copper environmental burdens are derived using data of primary copper (mined with molybdenum), primary copper (mined with platinum group metals, South Africa), primary copper (mined with platinum group metals, Russia), primary copper (co-product with gold, silver, zinc, and lead), primary copper (co-product with nickel), and secondary copper [4] (see Table S38). The process represents an aggregation of the pyrometallurgical and solvent extraction-electro winning (SX-EW) process. Allocation between the copper concentrate and molybdenum concentrate is based on year 2006-2010 price averages as shown in the following table.

Table S6 Allocation of environmental burdens between copper- and molybdenum concentrate.

|  | 2006-2010 Price ($/kg) | Percentage in concentrate | Metal content | Price per kg concentrate ($/kg) | Amount (kg) | Allocation (%) |
| --- | --- | --- | --- | --- | --- | --- |
| Cu concentrate, couple production MO/GLO U | 6.45^a^ | 100% | 30% | 1.92 | 1 | 94% |
| Mo concentrate, couple production CU/GLO | 49.03 | 93% | 60% | 27.21 | 0.00411 | 6% |

^a^ The price of the copper concentrate is derived by subtracting treatment and refining charges (TC/RCs) of $70 a metric ton and 7 cents a lb^[[1]](#footnote-1)^.

Around 85% of copper is produced from primary ores in 2008 and 15% from secondary sources. The breakdown of primary copper by source metal is shown in Table S7. These percentages are used to weigh each primary production route. Secondary production is modeled using the reallocated ecoinvent unit processes for “Copper secondary, at refinery RER/U” and “copper, secondary, from electronic and electric scrap recycling, at refinery/SE U” assuming equal production shares for both.

Table S7 Copper output by source metal in year 2005 [38].

| **Main metal** | **2004** | **2005 (used in this study)** |
| --- | --- | --- |
| Cu | 89.3% | 89.8% |
| Ni | 4.7% | 4.7% |
| Au | 2.0% | 2.4% |
| Zn/Pb | 1.6% | 1.5% |
| Pt/Pd (mostly Russia) | 0.2% | 0.2% |
| Ag | 0.2% | 0.1% |
| Blank entry | 1.9% | 1.2% |

1. **Zinc (Zn, Z=30)**

The zinc environmental burdens are derived using data of primary zinc (from lead-zinc ores with cadmium, indium and germanium as co-products), and primary zinc (from gold-silver mining with zinc as co-product) [4] (see Table S38). Please see the Germanium entry for details on the leas-zinc production system.

1. **Gallium (Ga, Z=31)**

The life cycle inventory for gallium is based on the Ecoinvent entry “Gallium, semiconductor grade, at plant/GLO U” [13] [4] (see Table S38). The most important production process is the extraction of gallium from circulating liquors of the Bayer process for aluminum oxide manufacture [39].

1. **Germanium (Ge, Z=32)**

The environmental burdens of germanium, indium, and cadmium, are based on the ecoinvent entries for lead/zinc production [13]. These are presented in further detail below and in a forthcoming metals criticality paper by Harper et al (2013) [40].

Germanium is produced by processing zinc leaching residues, which can also contain gallium and indium (Scoyer et al. 2002), and Figure S3 yields insight into this process.

Figure S3 Illustration of processes and flows from the mining of zinc-lead deposits (adapted from [13]). GLO: Global. RER: Europe.


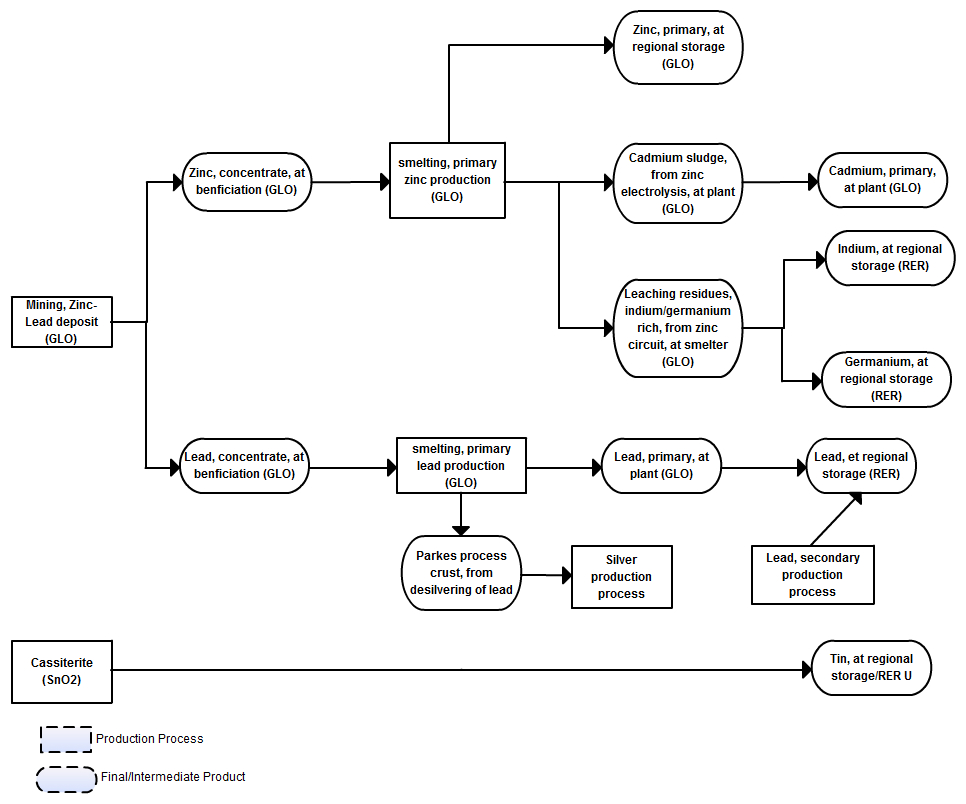


Allocation is based on year 2006-2010 average prices from (USGS 2013) (Table S8 (a)), taking into account the total value of metals present in the zinc and lead concentrate. Cadmium, indium, and germanium are jointly extracted with the zinc during its primary production. Table S8 (b) shows the allocation factors for the four products, zinc, cadmium, indium and germanium using revenue. Following the Ecoinvent approach by [13], 10% of the metal prices of cadmium, indium and germanium is taken to estimate the value of each residue recovered from zinc smelting (representing a 10% profit margin). This is done as the intermediate substances obtained are not usually sold into the market but require further refining. Allocation of environmental burdens associated with purification and recovery of indium and germanium from the leaching residues is based on 2006-2010 average prices. Allocation between lead and silver-containing crust (Table S8 (c)) is based on 2006-2010 average prices, with 10% of the average market price used for the intermediate product containing silver [13].

**Table S8** Relevant parameters to determine the allocation split for (a) lead and zinc mining and concentration, (b) zinc smelting and production, and (c) lead and silver-containing crust, based upon [13].

(A)

|  | **Zn concentrate [kg]** | **Pb concentrate [kg]** | **$/kg**  **(year 2006-2010)** | **Assumed concentration in concentrate** |
| --- | --- | --- | --- | --- |
| Output concentration | 6.26E-01 | 3.74E-01 |  |  |
| Pb |  | 2.06E-01 | 2.26 | 55% |
| Zn | 3.32E-01 |  | 2.57 | 53% |
| Ag |  | 2.99E-04 | 481.36 | 0.080% |
| Cd | 2.07E-03 |  | 4.66 | 0.330% |
| In | 6.88E-05 |  | 692.60 | 0.011% |
| Ge | 1.19E-05 |  | 1172.00 | 0.002% |
|  |  |  |  |  |
| Total value ($) | 9.23E-01 | 6.09E-01 |  |  |
| Reallocation (Our study) | 60% | 40% |  |  |
| Previous (Ecoinvent) | 79% | 21% |  |  |

(B)

| **Metal** | **Quantity [kg]** | **Metal Content [%]** | **Metal output (Quantity x Metal content) [kg]** | **Mass allocation [%]** | **$/kg** | **Value contained in $** | **Economic allocation [%]** | **Previous** |
| --- | --- | --- | --- | --- | --- | --- | --- | --- |
| Zn | 1 | 100.00% | 1 | 99.84% | 2.57 | 2.57 | 99.85% | 99.86% |
| Cd | 0.00338 | 45.00% | 0.001521 | 0.15% | 0.47^1^ | 0.0007 | 0.03% | - |
| In | 0.34 | 0.01% | 0.000034 | 0.003% | 69.26^1^ | 0.0024 | 0.09% | 0.14% |
| Ge |  | 0.0017% | 0.00000578 | 0.001% | 117.20^1^ | 0.0007 | 0.03% |  |

^1^Following (Classen et al., 2009), 10% of the metal price is taken to estimate the residue values.

(C)

| **Lead/Silver bearing concentrate** | **Process yield** | **lead concentrate** | **Parkes process crust** | **lead** | **Value in $/kg** |
| --- | --- | --- | --- | --- | --- |
| composition |  | input | output | output |  |
| Pb | 98% | 55% | 39% | 100% | 2.26 |
| Ag | 97% | 0.08% | 18% | 0% | 48.14^1^ |
| Amount | kg | 1.86 | 0.0081 | 1 |  |
| Value contained in $ | $ |  | 0.07 | 2.26 |  |
| Allocation by value |  |  | 3.01% | 96.99% |  |
|  |  | Ecoinvent | 3% | 97% |  |

^1^Following (Classen et al., 2009) [13], 10% of the metal price is taken to estimate the value of silver containing Parkes process crust.

1. **Arsenic (As, Z=33)**

Arsenic is obtained mostly as a byproduct from the smelting of nonferrous metal ores (i.e., gold, silver, lead, copper, nickel, and cobalt) [41]. This study models arsenic as a byproduct from copper smelting using the ecoinvent unit process for blister copper [13], and the ProBas entry for metallic arsenic [42].

**Arsenic trioxide.** During mining and beneficiation of copper ores, arsenic is present in the copper concentrate at concentrations of 0.5 – 1% [41], with a concentration of 0.75% used in this study. Given the molecular mass of arsenic and arsenic trioxide, this equals 2% arsenic trioxide. Globally, copper is present in the copper concentrate at a concentration of 29.7% [13]. The separation of the arsenic (as arsenic trioxide) takes place during roasting (pyrometallurgy) of the copper concentrate. Arsenic is distributed between mattee and slag, or blister copper and slag [41]. In this study, the unit process for blister copper^[[2]](#footnote-2)^ (98% copper) [13] is used to approximate the environmental burden of arsenic production. Copper is recovered at a 97% yield [13] and arsenic trioxide at a 100% yield (own assumption). The ecoinvent entry for blister copper [13] is modified by replacing the upstream link to copper concentrate from Europe (RER) (4.06 kg per kg blister copper) with copper concentrate produced globally (GLO) (3.48 kg per kg blister copper) which is reallocated between copper and molybdenum produced as byproduct described in section 17. The allocation percentages (based on 2006-2010 price data) are shown in the table below.

Table S9 Allocation between blister copper and arsenic trioxide.

|  | Input of Cu concentrate (kg) | Percent in roast (%) | Yield (%) | 2006-2010 price (US$/kg) | Allocation Percentage (%) | Output (kg) |
| --- | --- | --- | --- | --- | --- | --- |
| **Cu** | 3.41^a^ | 29.7% | 97% | $ 6.70 | 99.53% | 1.00 |
| **As2O3** |  | 2.0%^b^ | 100% | $ 0.46 | 0.47% | 0.07 |

^a^ 3.48 kg x 98% = 3.41 kg (the copper content in blister copper equals 98%). ^b^ 0.75% arsenic x M_As2O3_/M_As_ = 1.98% As_2_O_3_.

**Arsenic (metallic).** Commercial-grade metallic arsenic is obtained by reducing arsenic trioxide, e.g., with carbon [41]. The reaction is endothermic and requires external heat. The inputs and types of energy, water requirements, land occupation, and emissions to air and water are based on the ProBas entry “Arsen” [42]. Since the ProBas dataset represents arsenic production at system process level (i.e., inputs and outputs at elementary flow level after allocation between copper and arsenic), it includes the environmental burdens of intermediate arsenic trioxide production. In this study the difference between the environmental burdens of metallic arsenic provision (ProBas entry - Table S10) and arsenic trioxide provision (see entry based on blister copper above) is reported as the environmental impact of upgrading arsenic trioxide into metallic arsenic.

Table S10 Life cycle inventory showing process names chosen in the Ecoinvent database for 1 metric ton of arsenic production as byproduct from copper. The inventory is based on data given in the ProBas database [42] and is publicly available at http://www.probas.umweltbundesamt.de.

| **Inputs** | Amount | Unit | Distribution | StDv95% | Notes |
| --- | --- | --- | --- | --- | --- |
| Biomass | 7.37 | kg | Lognormal | 1.25 | (2,2,2,5,1,5) |
| Occupation, arable | 10.4 | m2a | Lognormal | 1.58 | (2,2,2,5,1,5) |
| Occupation, industrial area | 139 | m2a | Lognormal | 1.58 | (2,2,2,5,1,5) |
| Transformation, from unknown | 1.12 | m2 | Lognormal | 2.07 | (2,2,2,5,1,5) |
| Energy, from biomass | 1866 | MJ | Lognormal | 1.25 | (2,2,2,5,1,5) |
| Energy, from coal | 12380 | MJ | Lognormal | 1.25 | (2,2,2,5,1,5) |
| Energy, from uranium | 2264 | MJ | Lognormal | 1.25 | (2,2,2,5,1,5) |
| Energy, unspecified | 0.116 | MJ | Lognormal | 1.25 | (2,2,2,5,1,5) |
| Copper ore, in ground | 23.9 | ton | Lognormal | 1.25 | (2,2,2,5,1,5) |
| Water, cooling, drinking | 18862 | kg | Lognormal | 1.25 | (2,2,2,5,1,5) |
| **Outputs** |  |  |  |  |  |
| Arsenic, metallic |  |  |  |  |  |
| **Emissions to air** |  |  |  |  |  |
| Trichloroethane | 5.63E-10 | kg | Lognormal | 2.07 | (2,2,2,5,1,5) |
| Ethane, 1,2-dichloro- | 3.56E-05 | kg | Lognormal | 2.07 | (2,2,2,5,1,5) |
| Arsenic | 7.23 | kg | Lognormal | 5.07 | (2,2,2,5,1,5) |
| Benzo(a)pyrene | 0.00517 | kg | Lognormal | 3.06 | (2,2,2,5,1,5) |
| Benzene | 0.00517 | kg | Lognormal | 2.07 | (2,2,2,5,1,5) |
| Lead | 0.0542 | kg | Lognormal | 5.07 | (2,2,2,5,1,5) |
| Cadmium | 0.00237 | kg | Lognormal | 5.07 | (2,2,2,5,1,5) |
| Chlorinated fluorocarbons, soft | 1.72E-05 | kg | Lognormal | 2.07 | (2,2,2,5,1,5) |
| Methane, biogenic | 0.00767 | kg | Lognormal | 1.58 | (2,2,2,5,1,5) |
| Methane | 2.33 | kg | Lognormal | 1.58 | (2,2,2,5,1,5) |
| Chromium | 0.015 | kg | Lognormal | 5.07 | (2,2,2,5,1,5) |
| Ozone | 0 | kg | Undefined |  | (2,2,2,5,1,5) |
| Carbon monoxide | 19.2 | kg | Lognormal | 5.07 | (2,2,2,5,1,5) |
| Carbon dioxide, fossil | 998 | kg | Lognormal | 1.25 | (2,2,2,5,1,5) 331814-(1600kg CO2-eq/kg CFC11*14kg CFC-11)=309414 |
| Carbon monoxide, biogenic | 17.3 | kg | Lognormal | 5.07 | (2,2,2,5,1,5) |
| Methane, dichloro-, HCC-30 | 1.76E-08 | kg | Lognormal | 1.58 | (2,2,2,5,1,5) |
| Dioxins (unspec.) | 2.62E-09 | kg | Lognormal | 1.58 | (2,2,2,5,1,5) |
| Ethene | 0.0134 | kg | Lognormal | 1.58 | (2,2,2,5,1,5) |
| Particulates, < 10 um | 9.9 | kg | Lognormal | 2.07 | (2,2,2,5,1,5) |
| Hydrogen sulfide | 0.0115 | kg | Lognormal | 1.58 | (2,2,2,5,1,5) |
| Hydrogen chloride | 0.0838 | kg | Lognormal | 1.58 | (2,2,2,5,1,5) |
| Benzene, hexachloro- | 1.26E-07 | kg | Lognormal | 1.58 | (2,2,2,5,1,5) |
| Hydrogen fluoride | 0.0109 | kg | Lognormal | 1.58 | (2,2,2,5,1,5) |
| Methane, trichlorofluoro-, CFC-11 | 0.0527 | kg | Lognormal | 1.58 | (2,2,2,5,1,5) Based on stratospheric ozone depletion potential in total. |
| Copper | 0.0898 | kg | Lognormal | 1.58 | (2,2,2,5,1,5) |
| Nitrogen monoxide | 0.0917 | kg | Lognormal | 1.58 | (2,2,2,5,1,5) |
| Ammonia | 1.08 | kg | Lognormal | 1.58 | (2,2,2,5,1,5) |
| Nickel | 0.0218 | kg | Lognormal | 5.07 | (2,2,2,5,1,5) |
| NMVOC, non-methane volatile organic compounds, unspecified origin | 21.7 | kg | Lognormal | 1.58 | (2,2,2,5,1,5) |
| Nitrogen oxides | 7.36 | kg | Lognormal | 1.58 | (2,2,2,5,1,5) |
| Polychlorinated biphenyls | 1.83E-07 | kg | Lognormal | 3.06 | (2,2,2,5,1,5) |
| Phenol, pentachloro- | 1.69E-06 | kg | Lognormal | 2.07 | (2,2,2,5,1,5) |
| Mercury | 8.33E-05 | kg | Lognormal | 5.07 | (2,2,2,5,1,5) |
| Sulfur dioxide | 16.4 | kg | Lognormal | 1.58 | (2,2,2,5,1,5) |
| Zinc | 0.0569 | kg | Lognormal | 5.07 | (2,2,2,5,1,5) |
| **Emissions to water** |  |  |  |  |  |
| Arsenic | 0.26 | kg | Lognormal | 5.07 | (2,2,2,5,1,5) |
| Benzo(a)pyrene | 0 | kg | Lognormal | 3.06 | (2,2,2,5,1,5) |
| Benzene | 0.00267 | kg | Lognormal | 3.06 | (2,2,2,5,1,5) |
| Lead | 0.000524 | kg | Lognormal | 5.07 | (2,2,2,5,1,5) |
| BOD5, Biological Oxygen Demand | 1.81 | kg | Lognormal | 1.58 | (2,2,2,5,1,5) |
| Cadmium | 4.26E-05 | kg | Lognormal | 5.07 | (2,2,2,5,1,5) |
| Chloride | 5.51 | kg | Lognormal | 1.58 | (2,2,2,5,1,5) |
| Chromium | 0.00392 | kg | Lognormal | 5.07 | (2,2,2,5,1,5) |
| COD, Chemical Oxygen Demand | 3.49 | kg | Lognormal | 1.58 | (2,2,2,5,1,5) |
| Cyanide | 0.0702 | kg | Lognormal | 1.58 | (2,2,2,5,1,5) |
| Fluoride | 0.0212 | kg | Lognormal | 1.58 | (2,2,2,5,1,5) |
| CFCs, unspecified | 2.23E-05 | kg | Lognormal | 3.06 | (2,2,2,5,1,5) |
| Butadiene, hexachloro- | 0 | kg | Lognormal | 3.06 | (2,2,2,5,1,5) |
| Copper | 0.0139 | kg | Lognormal | 5.07 | (2,2,2,5,1,5) |
| Ammonia | 0.0077 | kg | Lognormal | 1.58 | (2,2,2,5,1,5) |
| Nickel | 0.275 | kg | Lognormal | 5.07 | (2,2,2,5,1,5) |
| Nitrate | 0.0272 | kg | Lognormal | 1.58 | (2,2,2,5,1,5) |
| Tin | 5.92E-06 | kg | Lognormal | 5.07 | (2,2,2,5,1,5) |
| TOC, Total Organic Carbon | 1.28 | kg | Lognormal | 1.58 | (2,2,2,5,1,5) |
| Phenol | 0 | kg | Lognormal | 3.06 | (2,2,2,5,1,5) |
| Polychlorinated biphenyl, PCB-1254 | 0.000328 | kg | Lognormal | 3.06 | (2,2,2,5,1,5) |
| Mercury | 6.85E-06 | kg | Lognormal | 5.07 | (2,2,2,5,1,5) |
| Sulfate | 32.7 | kg | Lognormal | 1.58 | (2,2,2,5,1,5) |
| Phosphorus | 0.0105 | kg | Lognormal | 1.58 | (2,2,2,5,1,5) |
| Nitrogen, total | 0.346 | kg | Lognormal | 1.58 | (2,2,2,5,1,5) |
| Zinc | 0.00333 | kg | Lognormal | 5.07 | (2,2,2,5,1,5) |

The global end-use shares of arsenic trioxide and metallic arsenic used are as follows:

| Use | Percentage into use^a^ | Chemical form [15] |
| --- | --- | --- |
| Wood preservation and pesticides | 68 | As_2_O_3_ |
| Semiconductors | 7 | As metal |
| Copper alloys | 7 | As metal |
| Other | 18 | As_2_O_3_ |

^a^ Based on estimates for the United States as given in Nassar et al (2012) [43]

1. **Selenium (Se, Z=34)**

Selenium is obtained together with tellurium and silver from copper refining, during which the three elements accumulate in the anode slimes. The unit processes for selenium, tellurium, and silver, are based on revised ecoinvent datasets described in further detail in Classen et al. (2009) [13]. Selenium’s upstream environmental burdens are included in the assessment as described below.

Allocation of environmental burdens takes place at multiple stages during the product chain and is illustrated in Figure S4. Each stage is described in further detail below.

Figure S4 Copper multioutput system modeled in this study.


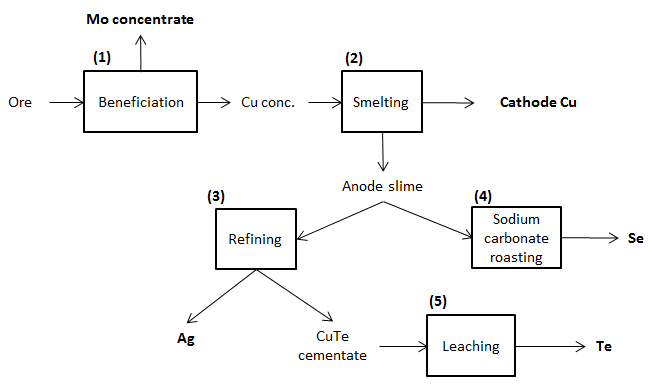


**Stage 1. Allocation between copper and molybdenum concentrates**

The allocation between copper concentrate and molybdenum is described in the copper entry above (section 17).

**Stage 2. Allocation between cathode copper and anode slime**

The allocation between cathode copper and anode slime is based on the 2006-2010 market values of all product constituents. The effective amount of elements present in the anode slime is based on global production as shown in Table S11.

Table S11 Production factors for the byproducts silver, tellurium, and selenium, in the anode slime from copper production (created using the example in Classen et al. (2009) [13]). Production data comes from USGS Mineral Yearbooks [26].

| **Year** | **Copper mine production (metric tons/year)** | **Silver from copper (metric tons/year)** | **Tellurium from copper (metric tons/year)** | **Selenium from copper (metric tons/year)** | **Silver per copper (metric tons/metric tons)** | **Tellurium per copper (metric tons/metric tons)** | **Selenium per copper (metric tons/metric tons)** |
| --- | --- | --- | --- | --- | --- | --- | --- |
| 2006 | 15,400,000 | 5,200 | 104 | 1,986 | 3.38E-04 | 6.78E-06 | 1.29E-04 |
| 2007 | 15,400,000 | 5,200 | 112 | 1,980 | 3.38E-04 | 7.25E-06 | 1.29E-04 |
| 2008 | 15,700,000 | 5,225 | 115 | 1,962 | 3.33E-04 | 7.31E-06 | 1.25E-04 |
| 2009 | 15,900,000 | 5,450 | 116 | 1,971 | 3.43E-04 | 7.31E-06 | 1.24E-04 |
| 2010 | 16,200,000 | 5,775 | 111 | 1,908 | 3.56E-04 | 6.83E-06 | 1.18E-04 |
| **Average** | 15,720,000 | 5,370 | 112 | 1,961 | **3.41E-04** | **7.10E-06** | **1.25E-04** |

^a^Assuming that copper refining accounts for 90% of global selenium and tellurium production, and 25% of global silver production [13].

As shown in the table, from 2006-2010 on average 341 g silver, 7.1 g tellurium, and 125 g selenium were obtained per metric ton of cathode copper produced. The total amount of anode slime generated per metric ton of cathode copper equals 1.63 kg [13]. Using 2006-2010 price averages, the allocation between the anode slime (used in subsequent recovery of sleneium, tellurium, and silver) and the cathode copper (used in subsequent production of primary copper) can be calculated as shown in Table S12.

Table S12 Revenue allocation between cathode copper and anode slime.

| **Allocation** | **kg output** | **$/kg** | **Allocation %** |
| --- | --- | --- | --- |
| **Cathode Copper** | **1.00E+00** | **$ 6.70** | **97.47%** |
| Silver in anode slime | 3.41E-04 | $ 481.36 | 2.39% |
| Tellurium in anode slime | 7.10E-06 | $ 150.40 | 0.02% |
| Selenium in anode slime | 1.25E-04 | $ 66.51 | 0.12% |
| **Anode Slime (total)** | **1.63E-03** | **$ 10.66^a^** | **2.53%** |

^a^Following Classen et al. (2009), a value of 10% of the price of the refined commodity (selenium, tellurium, silver) is used to calculate the market price of the intermediate product anode slime.

**Stage 3. Allocation between silver (metallic) and CuTe cementate**

This unit process generates metallic silver and CuTe cementite from anode slime. It is based on the multiouput process “ Silver and coppertelluride cement, from copper production” in Ecoinvent [13]. Given the calculations in stage 2, the silver content in the anode slime equals 21% and the tellurium content 0.44%. The recovery yields are 100% for silver (closed cycle) and 90% for tellurium [13]. Allocation based on 2006-2010 price averages gives an allocation percentage of all upstream environmental burdens of 99.9% to silver and 0.1% to tellurium (Table S13). This is similar to what is reported in the original Ecoinvent report [13].

Table S13 Updated allocation percentages of anode slime recovery towards silver and CuTe cementate.

| Element | Percent per kg anode slime | Yield (%) | 2006-2010 price (US$/kg) | Allocation Percentage (%) | kg per kg Ag |
| --- | --- | --- | --- | --- | --- |
| Ag | 20.95% | 100% | $ 481.36 | 99.9% | 1.00E+00 |
| Te | 0.44% | 90% | $ 15.04^a^ | 0.1% | 4.16E-02^b^ |

^a^ Following Classen et al (2009) [13], 10% of the market price of tellurium (2006-2010 price average) is used because the CuTe cementate represents an intermediate product. Silver on the other hand represents the final product and therefore the full market price is used. ^b^ The tellurium content in CuTe cementate equals 50%.

**Stage 4. Allocation between silver/CuTe cementate and selenium**

Selenium is obtained from the anode slime via sodium carbonate roasting [4]. While the original Ecoinvent dataset does not include the upstream burden of anode slime production, in this study the selenium production process is linked to the anode slime using the 5-year price averages shown in Table S14. According to this, the environmental burdens associated with the generation of 0.02 kg of anode slime are allocated towards the selenium. It should be noted that resource inputs of selenium from ground still need to be allocated based on the selenium content in the anode slime. This has been excluded here as this assessment is not concerned with resource depletion related impacts.

Table S14 Inclusion of the upstream environmental burdens of anode slime production in the selenium life cycle.

|  | Percent per kg anode slime | Yield (%) | 2006-2010 price (US$/kg) | Allocation Percentage (%) | kg per kg Ag | Old anode slime input (Ecoinvent) | New anode slime input (this study) |
| --- | --- | --- | --- | --- | --- | --- | --- |
| Ag | 20.95% | 100% | $ 481.36 | 99.5% | 1.00E+00 | 4.995 | 4.977 |
| Te | 0.44% | 90% | $ 15.04 | 0.1% | 2.08E-02 | 0.005 | 0.003 |
| Se | 7.66% | 80% | $ 6.65 | 0.4% | 3.66E-01 | 0 | **0.020** |

**Stage 5. Leaching of CuTe cementate towards metallic Te**

This stage is based on the ecoinvent process “tellurium, semiconductor grade, at plant” from the ecoinvent database [13].

1. **Strontium (Sr, Z=38)**

Strontium compounds are obtained from the minerals strontianite (SrCO_3_) and celestite (SrSO_4_). However, while strontianite was of great importance for strontium production between 1870 and 1920, it has now been replaced largely by celestite, mined from its own ores [44]. In 2008, the majority of celestite originated from Spain (51%), China (30%), Mexico (15%), and others (4%) [15].

Celestite is mined from both open-cast and underground mines. Hand picking can give an intermediate material containing >90% SrSO_4_ [44]. Impurities are removed via desliming and celestite separated from other ores by means of density separation. Strontium carbonate, used in ceramics and glasses, is the principal strontium compound consumed [45]. The main production route uses hot sodium carbonate for conversion of ground celestite. It is also the starting material for a variety of other strontium compounds. Another important strontium compound, strontium nitrate used in fireworks, is produced from celestite in nitric acid. Strontium metal, used e.g. in master alloys such as 10% Sr – 90% Al or 90% Sr – 10% Al to make aluminum more suitable for casting [45], is produced by the Pidgeon process in which the material reacts with aluminum under a vacuum. Table S15 shows typical strontium end-uses in the United States in 2004.

Table S15 End-uses of strontium in the United States in 2004 [45].

| **End-use percentage (%)** | **End-use type** | **Form used** |
| --- | --- | --- |
| 75 | Ceramics/Glass (e.g. cathode ray tubes, X-ray absorbing glass) | SrCO_3_ |
| 9 | Ferrite ceramic magnets | SrO∙6Fe_2_O_3_ (from SrCO_3_) |
| 9 | Fireworks | SrNO_3_ |
| 7 | Others | Various |

**Mining and beneficiation**

Celestite is mined both in open-cast and underground mines [44], but the exact global share of both operations unknown. Open-cast mining is assumed in this assessment. During mining, rock is broken by drilling and blasting and the raw ore is mined using backhoes and front end loaders [46]. Roughly 47-87 kg celestite (3.8-3.9 g/cm3) are contained in one cubic meter of ore containing limestone, quartz, gypsum, and dolomite (all <3 g/cm3) [44]. For explosives used, a typical amount of 0.26kg/t from manganese mining [13] is used. The energy use is approximated from barite mining [47] to be 0.07 MJ electricity per kg ore (weighted by 2008 producing countries, i.e., Spain (51%), China (30%), Mexico (15%), and others (4%)). Similarly, water use is approximated with a number from barite mining (12600 l water/m3 ore) [47]. The overburden is assumed to be disposed in piles near the mine (the standard Ecoinvent module for disposal of non-sulphidic overburden was chosen). Once the ore is mined, it is ground and celestite separated from limestone, quartz, gypsum, and dolomite in a flotation plant [44]. Assuming a yield of 67 kg celestite/m3 ore, a density of 3.85 t/m3 ore, and a recovery efficiency of 75%, around 43 kg of ore are mined per kg of celestite. The infrastructure of mining and beneficiation is included via respective generic Ecoinvent datasets [13]. No data on emissions to air, soil, and water is available from the literature. While the celestite concentrates from the Montevieve’s mine in Spain (operated by Bruno SA) contain around 95% SrSO_4_ for the hand sorted concentrate and around 90% for the crushed and graded concentrate, most of China’s celestite concentrates contain less than 80% SrSO_4_ [46]. The final product from mining and beneficiation is celestite containing 90% SrSO_4_. The life cycle inventory is shown in Table S16.

Table S16 Life Cycle Inventory for celestite mining and beneficiation.

| **Input** | **Unit** | **Amount** | **Distribution** | **StDv95%** | **Notes** |
| --- | --- | --- | --- | --- | --- |
| Celestite, in crude ore, in ground | kg | 43.1 | Lognormal | 1.69 | (4,5,5,5,1,5) Assuming a yield of 67 kg celestite/m3 ore, a density of 3.85 t/m3 ore, and a recovery efficiency of 75%. |
| Water | m3 | 0.14 | Lognormal | 1.69 | (4,5,5,5,1,5) Same value per kg ore as in barite mining [47] |
| Electricity | kWh | 0.84 | Lognormal | 1.69 | (4,5,5,5,1,5) Same value per kg ore as in barite mining [47]. Spain (51%), China (30%), Mexico (15%), and others (4%) [15] |
| Blasting | kg | 0.011 | Lognormal | 1.69 | (4,5,5,5,1,5) Same value per kg ore as for manganese mining [13] |
| Conveyor belt, at plant | unit | 3.28E-06 | Lognormal | 1.69 | (4,5,5,5,1,5) same values as in copper exploitation (7.6E-5m/t ore) [13] |
| Non-ferrous metal mine, surface | unit | 2.15E-09 | Lognormal | 3.38 | (4,5,5,5,1,5) Generic Ecoinvent process, 20,000Mt assumed during 50 year lifetime [13] |
| Transformation from unknown | m2 | 2.57E-03 | Lognormal | 2.38 | (4,5,5,5,1,5) Based on non-ferrous metals processing estimate from [13] |
| Transformation, to mineral extraction site | m2 | 2.57E-03 | Lognormal | 2.38 | (4,5,5,5,1,5) Based on non-ferrous metals processing estimate from [13] |
| Transformation, to unknown | m2 | 2.57E-03 | Lognormal | 2.38 | (4,5,5,5,1,5) Based on non-ferrous metals processing estimate from [13] |
| Occupation, mineral extraction site | m2a | 3.02E-01 | Lognormal | 1.94 | (4,5,5,5,1,5) Based on non-ferrous metals processing estimate from [13] |
| **Ouput** |  |  |  |  |  |
| Strontium sulfate (90%), at plant |  | 1 |  |  |  |
| Disosal, non-sulfidic overburden |  | 42.1 | Lognormal | 1.69 | (4,5,5,5,1,5) [13] |

**Strontium carbonate production**

Commercial strontium carbonate is produced via the black ash process, in which the crushed celestite concentrate is fed into rotary kilns and mixed with ground coke [45]. The mixture is then heated to 1,100 ºC. The chemical reaction describing the process is:

SrSO_4_ + 2 C 🡪 SrS + 2 CO_2_

The strontium sulfide intermediate is collected and fed into a leacher circuit where it is dissolved in water and the muds separated via decantation, collected in a filter press, and discarded after washing. The solution contains 12 to 13 wt% strontium sulfide which is precipitated as strontium carbonate in agitation tanks using sodium carbonate solution.

SrS + Na_2_CO_3_ 🡪 SrCO_3_ + Na_2_S

The final product is a filter cake with 60 w % strontium carbonate which is further dried to obtain 90% strontium carbonate. Sodium sulfide is generated as byproduct product. The inputs of reaction substances and the generation of carbon dioxide per kg strontium carbonate produced is based solely on stoichiometric relationships, assuming a yield of 80%. No information on inputs of heat and water, and outputs of effluent are available and they are therefore excluded from the analysis.

Table S17 Life Cycle Inventory for the production of strontium carbonate. Based on stoichiometric relationships, assuming a yield of 80%.

| **Input** | **Unit** | **Amount** | **Distribution** | **StDv95%** | **Notes** |
| --- | --- | --- | --- | --- | --- |
| Strontium sulfate (90%), at plant | kg | 1.64 | Lognormal | 1.69 | (4,5,5,5,1,5) 80% recovery efficiency |
| Hard coal coke | MJ | 6.14 | Lognormal | 1.69 | (4,5,5,5,1,5) Carbon content = 0.9kgC/kg, Heating value = 28.6MJ/kg |
| Sodium carbonate | kg | 0.85 | Lognormal | 1.69 | (4,5,5,5,1,5) |
| Water | kg | unknown | Lognormal |  | Unknown and hence excluded from the analysis |
| Heat | MJ | unknown | Lognormal |  | Unknown and hence esxlcuded from the analysis |
| **Output** |  |  |  |  |  |
| Strontium carbonate (95%) | kg | 1.00 |  |  | Main product (all environmental burdens allocated to the main product) |
| Sodium sulfide | kg | 0.50 | Lognormal |  | By-product (Burden-free) |
| Carbon dioxide (fossil) | kg | 0.71 | Lognormal | 1.69 | (4,5,5,5,1,5) |
| Effluent |  | unknown | Lognormal | - | Unknown and hence excluded from the analysis |

1. **Yttrium (Y, Z=39)**

Please see entry for lanthanum.

1. **Zirconium (Zr, Z=40)**

Zircon (ZrSiO_4_) is the predominant mineral source for zirconium production, but the minerals baddeleyite and eudialyte are also being utilized [48]. Zircon is mainly produced as a byproduct of heavy minerals sand mining and processing for the titanium minerals ilmenite and rutile [49]. Hafnium is available as a byproduct of zirconium metal and present in zircon at concentrations of worldwide roughly 2% of the zirconium content [50].

Zirconium oxide (zirconia) production is available from Ecoinvent [17]. 1.57 kg of zircon (50% zirconium) is required for the production of 1 kg of pure zirconium oxide. Hafnium oxide is generated as byproduct (mass allocation used). Production of subsequent zirconium and hafnium metal takes place via carbochlorination and Kroll reduction to obtain the metal sponge followed by vacuum arc melting for the generation of the pure metal ingot or alloy (Figure S5).

Figure S5 Overview of zirconium sponge production (adapted from [51]).


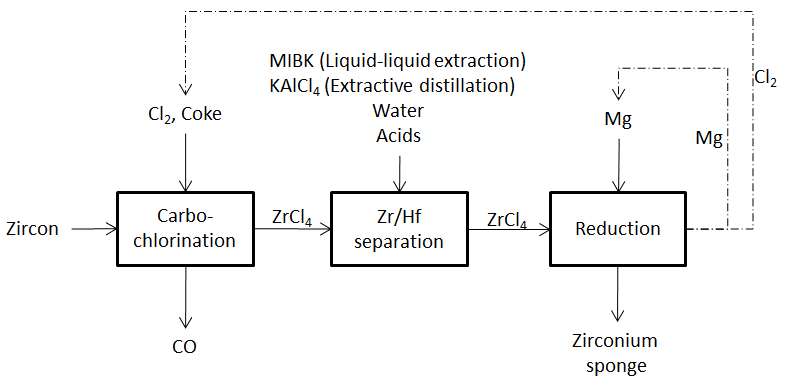


Inventory data on zirconium and hafnium metal production is not reported in the open literature [51]. The process consists of three steps (described in detail in [51] including: (1) Carbo-chlorination in which both zirconium and hafnium are chlorinated in a fluidized bed carbo-chlorinator using coke and chlorine at temperatures of 1200ºC, (2) Zirconium-hafnium separation using either liquid-liquid extraction (methyl isobutyl ketone (MIBK) is used as solving agent; this is the dominant process in the United States), or extractive distillation (molten potassium chloroaluminate (KAlCl_4_) is used for separation; this is the dominant process in France), and (3) Zirconium or hafnium sponge production process via the Kroll process using Magnesium (magnesium and chlorine generated are reused in the Kroll and carbo-chlorination process, respectively).

As no detailed information regarding the carbo-chlorination and zirconium-hafnium separation process could be obtained (see [51] for a discussion of data challenges), it was decided to base the life cycle inventory on estimates of energy inputs to the Kroll process and combine these with Ecoinvent data entries on zirconia and zircon production from [17]. 1.42 kg of zirconium oxide is required for the generation of 1 kg zirconium metal (95% yield). We use information on energy requirements of titanium metal production via the Kroll process (44.41 kWh/kg metal) from [52], and for arc melting of zirconium sponge (1.10 kWh/kg metal) from [53]. Energy inputs to the Kroll process were adjusted by the ratio of the enthalpies of formation *Δ_f_H^0^_298_* of ZrCl_4_ and TiCl_4_. The main producers of Zr/Hf metal are France, the United States, and Russia [49], with the bulk of production (roughly 50% each) taking place in the two former countries [54]. Using SimaPro8, we model electricity inputs to the Kroll reduction and arc melting to be equal shares from the U.S. and French power mix. Hafnium is a byproduct of zirconium production. Environmental burdens are allocated using average prices over the five-year period 2006-2010, as shown in USGS documents [55] (Table S18). Uncertainty of each parameter of the life cycle inventory is assessed using the Pedigree matrix [1,2]. The compiled life cycle inventory is shown in Table S19.

Table S18 Allocation factors applied to metal production from zirconium oxide.

|  | Amount (kg) | Value ($/kg) | Allocation factor |
| --- | --- | --- | --- |
| Zirconium metal | 0.98 | 40.4 | **87** |
| Hafnium metal | 0.02 | 299.8 | **13** |

Table S19 Life Cycle Inventory for the production of metallic zirconium and hafnium.

| **Input** | **Unit** | **Amount** | **Distribution** | **StDv95%** | **Notes** |
| --- | --- | --- | --- | --- | --- |
| Zirconium oxide | kg | 1.39 | Lognormal | 1.94 | (4,5,5,5,4,5) Stoichiometric calculation, 95% yield |
| Hafnium oxide | kg | 0.02 | Lognormal | 1.94 | (4,5,5,5,4,5) Stoichiometric calculation, 95% yield |
| Electricity | kWh | 22.31 | Lognormal | 1.9 | (2,5,5,5,4,5) [52,53]. French power mix |
| Electricity | kWh | 22.31 | Lognormal | 1.9 | (2,5,5,5,4,5) [52,53]. United States power mix |
| Non-ferrous metal smelter GLO U | unit | 7.55E-12 | Lognormal | 3.61 | (4,5,5,5,4,5) Generic Ecoinvent process. Lifetime 50 years, input 6750000 to ore concentrate (39%), output: 2650000 tons metal per year |
| Chlorine gas | kg | 0.00 | Lognormal | 1.94 | Recycled internally and not accounted for |
| Magnesium metal | kg | 0.00 | Lognormal | 1.94 | Recycled internally and not accounted for |
| **Output** |  |  |  |  |  |
| Zirconium metal ingots | kg | 0.98 | - | - | Metal ingot at the factory gate. 87% of environmental burdens allocated to zirconium metal |
| Hafnium metal ingots | kg | 0.02 | - | - | Metal ingot at the factory gate. 13% of environmental burdens allocated to hafnium metal |

1. **Niobium (Nb, Z=41)**

Roughly 77% of global niobium consumed is in the form of ferroniobium, mainly used as an additive in steelmaking [56]. The leading use for metallic niobium is in superalloys, amongst others for aircraft engines and high-temperature application [57,58]. We model both ferroniobium and metallic niobium routes as described below.

**Ferroniobium.** Ferroniobium is generally produced by aluminothermic reduction of niobiumoxide ores (e.g. pyrochlore) with the addition of iron oxides [57]. The LCI for niobium oxide (pyrochlore) production is taken from the ProBas database [42] and shown in the table below.

Table S20 Life cycle inventory showing process names chosen in the Ecoinvent database for 1 metric ton of niobium oxide production. The inventory is based on data given in the ProBas database [42] and is publicly available at http://www.probas.umweltbundesamt.de.

| **Input** | **Unit** | **Amount** | **Distribution** | **StDv95%** | **Notes** |
| --- | --- | --- | --- | --- | --- |
| **Resources** |  |  |  |  |  |
| Occupation, arable | m2a | 3.03 | Lognormal | 1.25 | (2,3,3,3,1,5) |
| Biomass | kg | 2.79 | Lognormal | 1.25 | (2,3,3,3,1,5) |
| Occupation, industrial area | m2a | 90.7 | Lognormal | 1.58 | (2,3,3,3,1,5) |
| Transformation, from unknown | m2 | 0.713 | Lognormal | 2.07 | (2,3,3,3,1,5) |
| Energy, potential (in hydropower reservoir), converted | MJ | 878 | Lognormal | 1.25 | (2,3,3,3,1,5) |
| Energy, from coal | MJ | 1929 | Lognormal | 1.25 | (2,3,3,3,1,5) |
| Energy, from uranium | MJ | 773 | Lognormal | 1.25 | (2,3,3,3,1,5) |
| Energy, unspecified | MJ | 0.0545 | Lognormal | 1.25 | (2,3,3,3,1,5) |
| Water, cooling, drinking | kg | 10192 | Lognormal | 1.25 | (2,3,3,3,1,5) |
| Niobium | ton | 13.4 | Lognormal | 1.25 | (2,3,3,3,1,5) |
| Metals n.e.c., extracted for use | ton | 0.0328 | Lognormal | 1.25 | (2,3,3,3,1,5) |
| Metals, n.e.c., related unused extraction | ton | 0.0245 | Lognormal | 1.25 | (2,3,3,3,1,5) |
| **Output** |  |  |  |  |  |
| Niobiumoxide (57.6% Nb2O5, 1.88% Ta2O5) | ton | 1 | - | - | - |
| **Emissions to air** |  |  |  |  |  |
| Trichloroethane | kg | 2.2E-10 | Lognormal | 1.25 | (2,3,3,3,1,5) |
| Ethane, 1,2-dichloro- | kg | 2.4E-05 | Lognormal | 1.25 | (2,3,3,3,1,5) |
| Arsenic | kg | 6.9E-05 | Lognormal | 1.64 | (4,3,3,3,1,5) |
| Benzo(a)pyrene | kg | 0.0012 | Lognormal | 2.11 | (4,3,3,3,1,5) |
| Benzene | kg | 0.0012 | Lognormal | 2.11 | (4,3,3,3,1,5) |
| Lead | kg | 0.00041 | Lognormal | 1.64 | (4,3,3,3,1,5) |
| Cadmium | kg | 4.3E-06 | Lognormal | 5.12 | (4,3,3,3,1,5) |
| Chlorinated fluorocarbons, soft | kg | 4.6E-06 | Lognormal | 2.11 | (4,3,3,3,1,5) |
| Methane, biogenic | kg | 0.00353 | Lognormal | 1.64 | (4,3,3,3,1,5) |
| Methane | kg | 0.336 | Lognormal | 1.64 | (4,3,3,3,1,5) |
| Chromium | kg | 0.0101 | Lognormal | 5.12 | (4,3,3,3,1,5) |
| Carbon monoxide | kg | 1.23 | Lognormal | 5.12 | (4,3,3,3,1,5) |
| Carbon dioxide, fossil | kg | 153 | Lognormal | 1.33 | (4,3,3,3,1,5) |
| Carbon monoxide, biogenic | kg | 3.35 | Lognormal | 5.12 | (4,3,3,3,1,5) |
| Methane, dichloro-, HCC-30 | kg | 9E-09 | Lognormal | 2.11 | (4,3,3,3,1,5) |
| Dioxins (unspec.) | kg | 1E-10 | Lognormal | 3.11 | (4,3,3,3,1,5) |
| Ethene | kg | 0.00091 | Lognormal | 2.11 | (4,3,3,3,1,5) |
| Particulates, < 10 um | kg | 5.55 | Lognormal | 3.11 | (4,3,3,3,1,5) |
| Formaldehyde | kg | 0.00021 | Lognormal | 2.11 | (4,3,3,3,1,5) |
| Ozone | kg | 0 | Lognormal | 1.64 | (4,3,3,3,1,5) |
| Hydrogen sulfide | kg | 0.00055 | Lognormal | 1.64 | (4,3,3,3,1,5) |
| Hydrogen chloride | kg | 0.0136 | Lognormal | 1.64 | (4,3,3,3,1,5) |
| Phosphate | kg | 0 | Lognormal | 1.64 | (4,3,3,3,1,5) |
| Benzene, hexachloro- | kg | 8.3E-08 | Lognormal | 2.11 | (4,3,3,3,1,5) |
| Hydrogen fluoride | kg | 0.00205 | Lognormal | 1.64 | (4,3,3,3,1,5) |
| Copper | kg | 0.00066 | Lognormal | 1.64 | (4,3,3,3,1,5) |
| Dinitrogen monoxide | kg | 0.0527 | Lognormal | 1.64 | (4,3,3,3,1,5) |
| Nickel | kg | 0.00066 | Lognormal | 5.12 | (4,3,3,3,1,5) |
| Nitrogen oxides | kg | 4.3 | Lognormal | 1.64 | (4,3,3,3,1,5) |
| Polychlorinated biphenyls | kg | 1.2E-07 | Lognormal | 3.11 | (4,3,3,3,1,5) |
| Anisole, pentachloro- | kg | 6.6E-07 | Lognormal | 2.11 | (4,3,3,3,1,5) |
| Mercury | kg | 1.8E-05 | Lognormal | 5.12 | (4,3,3,3,1,5) |
| Sulfur hexafluoride | kg | 1E-05 | Lognormal | 1.64 | (4,3,3,3,1,5) |
| Sulfur dioxide | kg | 0.534 | Lognormal | 1.64 | (4,3,3,3,1,5) |
| Zinc | kg | 0.0021 | Lognormal | 5.12 | (4,3,3,3,1,5) |
| Methane, trichlorofluoro-, CFC-11 | kg | 0.0107 | Lognormal | 2.11 | (4,3,3,3,1,5) |

Table S21 shows the raw materials needed for the provision of 1 kilogram ferroniobium.

Table S21 Raw material needs for aluminothermic provision of 1 kg ferroniobium.

| **Input** | **Unit** | **Amount** | **Distribution** | **StDv95%** | **Notes** |
| --- | --- | --- | --- | --- | --- |
| Pyrochlore concentrate (60% Nb2O5) | kg | 1.64 | Lognormal | 1.31 | (2,3,4,3,1,5) [57],  Pyrochlore inventory is from  [42] |
| Iron ore (65% Fe), at beneficiation/GLO U | kg | 0.36 | Lognormal | 1.31 | (2,3,4,3,1,5) [57] |
| Aluminum, primary, at plant/RER U | kg | 0.55 | Lognormal | 1.31 | (2,3,4,3,1,5) [57] |
| Fluorspar, 97%, at plant/GLO U | kg | 0.068 | Lognormal | 1.31 | (2,3,4,3,1,5) [57] |
| Limestone, milled, loose, at plant/CH U | kg | 0.045 | Lognormal | 1.31 | (2,3,4,3,1,5) [57] |
| Non-ferrous metal smelter/GLO/I U | unit | 7.55E-12 | Lognormal | 3.1 | (2,3,4,3,1,5) [13] |
| **Output** |  |  |  |  |  |
| Ferroniobium | kg | 1.00 |  |  | [57] |
| Disposal, refinery sludge, 89.5% water, to sanitary landfill/CH U | kg | 1.82 | Lognormal |  | (2,3,4,3,1,5) Proxy for slag  [57] |

We link each input to the respective unit process from Ecoinvent [4]. The provision of pyrochlore concentrate is modeled using the inventory entry^[[3]](#footnote-3)^ given in [42]. The smelting process takes place as a batch process in a refractory lined crucible. The reaction is exothermic with the heat being used as energy source for the process [59]. However, the enthalpy between the niobium concentrate and aluminum is slightly lower than the threshold value for self-sustaining aluminothermic reactions, therefore oxygen-releasing lime^[[4]](#footnote-4)^ is being added [57]. Using this data, the CED for ferroniobium production is 113 MJ-eq/kg and GWP equals 7.13 kg CO_2_-eq. This is slightly higher than the 2.1 kg CO_2_-eq/kg reported by IAMGOLD in Canada in their corporate sustainability report [60], and may be due to the fact that system-wide GWP associated with aluminum powder provision (the major contributor to GWP) is not included in their estimate and higher shares of hydropower for electricity inputs may be assumed.

**Pure niobium (>99.75%).** Environmental burdens were roughly estimated based on reports of embodied energy of primary production. In its materials database, CES 2012 Selector software [22] reports an embodied energy content for pure niobium of 720 MJ/kg (a range of 684 – 756 MJ/kg is given in the database). Final energy use during the niobium production process is roughly 65% heat, 25% electricity, and 10% from diesel use during mining (Table S22).

Table S22 Energy use for Niobium production in the early 1990s [61].

| **Niobium production process** | **GJ/t** | **%** | **Probable energy type** |
| --- | --- | --- | --- |
| Mining | 37.5 | 10.17 | Diesel Fuel |
| Beneficiation | 15.7 | 4.26 | Electricity |
| Chemistry | 87.2 | 23.65 | Coal |
| Reduction | 151.8 | 41.17 | Coal |
| Refinery | 76.5 | 20.75 | Electricity |
| **Total** | **369** | **100.00** |  |

Assuming an average conversion efficiency of 80% for heat, 35% for electricity, and 60% for diesel fuel burned in a machine, this equals 373 MJ/kg of final heat, 17.5 kWh of final electricity, and 44 MJ of final diesel demand. The major producers of Niobium are CBMM in Brazil and IAMGOLD in Canada [26]. Total production in 2008 was 63,000 metric tons, of which 92% was produced in Brazil and roughly 7% in Canada (Table S23).

Table S23 Niobium production statistics [26].

| **Country** | **Reserves^1^**  **(metric tons, 2008)** | **Production**  **(metric tons, 2008)** | **Global Share (%)** |
| --- | --- | --- | --- |
| Brazil | 246,650 | 58,000 | 92.0 |
| Canada | 13,300 | 4,383 | 7.0 |
| Others | 4,050 | 617 | 1.0 |
| Total | 264,000 | 63,000 | 100.0 |

^1^Nb/Ta minerals.

Cradle-to-gate environmental burdens are then approximated by using a weighted average electricity input for the two major producing countries (Brazil and Canada) using 2008 fuel shares from [25] and linking those to respective Ecoinvent electricity production processes in SimaPro. For heat inputs, a mix of 50% natural gas and 50% coal is used. A triangular uncertainty distribution with the min and max values provided by [22] is assumed.

Results for the 100-year GWP and CED, derived from SimaPro, are with 46.6 kg CO_2_-eq and 639 MJ-eq (all numbers per kg) similar to a GWP of 45.3 kg CO_2_-eq and CED of 720 MJ-eq reported by [22].

1. **Molybdenum (Mo, Z=42)**

The Mo environmental burdens are derived using inventory data of molybdenum metal, molybdenum concentrate (main product), and molybdenum concentrate (couple production from Cu ores) [13] (see Table S38). The copper entry above (Table S6) shows respective allocation percentages.

Rhenium is generated as co-product during molybdenum roasting and environmental burdens allocated based on 2006-2010 prices (see the rhenium entry).

1. **Ruthenium (Ru, Z=44)**

Please see the platinum entry.

1. **Rhodium (Rh, Z=45)**

Please see the platinum entry.

1. **Palladium (Pd, Z=46)**

Please see the platinum entry.

1. **Silver (Ag, Z=47)**

The environmental burdens of silver production are derived using data of primary silver (copper production), primary silver (lead production), primary silver (gold-silver production), and secondary silver (recovered with Au, Ni, Pb, Pd, and Cu in a precious metals refinery) [13] (see Table S38).

1. **Cadmium (Cd, Z=48)**

Please see the germanium entry.

1. **Indium (In, Z=49)**

Please see the germanium entry.

1. **Tin (Sn, Z=50)**

The life cycle inventory for tin is based on the Ecoinvent entry “Tin, at regional storage/RER U” [13]. The majority of tin is mined from its own ore cassiterite (SnO_2_) [62]. In 2008, 94.5% of global tin smelting production was from primary production and 5.5% from secondary sources (e.g. new scrap and tinplate) [15]. No inventory data on secondary tin production is available.

1. **Antimony (Sb, Z=51)**

Antimony is obtained mostly from its own ore (stibnite) and as a byproduct of the smelting of base metal ores (e.g. from copper, lead, and gold production) [15]. Some antimony is obtained from recycling of lead-acid batteries, where antimony is used as alloying element. In 2008, the majority of antimony was mined in China (91%) followed by Bolivia, Russia, and South Africa (each roughly 2%). China produces metallic antimony mainly from stibnite [13]. Besides metallic antimony, also antimony oxides are sold into the market. Due to a lack of more detailed data, we use inventory data for metallic antimony production from Ecoinvent as a proxy of global environmental burdens.

1. **Tellurium (Te, Z=52)**

Please see the selenium entry.

1. **Barium (Ba, Z=56)**

Today’s barium compounds are almost exclusively produced from barite (BaSO_4_) which is present in large deposits throughout the world [63]. In 2008, the main producers of barite were China (56%) and India (13%), followed by the United States (8%), Morocco (7%) and Turkey (2%) [15]. The majority of global barite produced (around 90%) is used as weighting agent in natural gas and oil field drilling, with the remainder going to barium chemicals production [15]. Global demand for barium metal, produced from barium oxide (a subsequent product from barite), is covered mostly by only one producer (Chemetall GmbH) in Germany [63].

As barite is almost exclusively used as the raw material for barium compounds, it is appropriate to investigate the environmental burdens of barite provision, rather than barium in its metallic form. We calculate environmental burdens from Ecoinvent inventory data for barite production [4].

**Barium metal (included for completion):**

Commercial barium metal is produced from barium oxide in a vacuum using aluminum as the reducing agent [63]. Barium oxide itself is obtained in multiple steps from barium sulfate (barite). The environmental burdens of barium metal are approximated as follows:

Step 1. Barium sulfate (barite). The mining and provision of barium sulfate is based on the ecoinvent unit process “Barite, at plant RER/U”.

Step 2. Barium carbonate production. Barite is transformed into barium oxide following a number of steps. First, the crushed barite (BaSO_4_ content ≥ 95w %) is fed into rotary kilns and mixed with ground coke. The mixture is then heated to 900 – 1200ºC. The reducing agent in the reaction is carbon monoxide formed from the carbon in the ground coke according to the Boudouard equilibrium [63]. The net chemical reaction can be described as:

BaSO_4_ + 2 C 🡪 BaS + 2 CO_2_

The barium sulfide intermediate is collected and placed into a 30% wt% sofa solution in agitator vessels. The final solution contains barium carbonate as well as diluted sodium sulfide solution which is typically upgraded to crystalline sodium sulfide hydrate. The chemical reaction is:

BaS + Na_2_CO_3_ 🡪 BaCO_3_ + Na_2_SO_4_

The inputs of reaction substances and the generation of carbon dioxide per kg strontium carbonate produced is based solely on stoichiometric relationships, assuming a yield of 80%. No information on inputs of heat and water, and outputs of effluent are available and they are therefore excluded from the analysis.

Table S24 Life Cycle Inventory for the production of calcium carbonate. Based on stoichiometric relationships, assuming a yield of 80%.

| **Input** | **Unit** | **Amount** | **Distribution** | **StDv95%** | **Notes** |
| --- | --- | --- | --- | --- | --- |
| Barite, at plant | kg | 1.48 | Lognormal | 1.69 | (4,5,5,5,1,5) 80% recovery efficiency |
| Hard coal coke | MJ | 4.83 | Lognormal | 1.69 | (4,5,5,5,1,5) Carbon content = 0.9kgC/kg, Heating value = 28.6MJ/kg |
| Sodium carbonate | kg | 0.67 | Lognormal | 1.69 | (4,5,5,5,1,5) |
| Water | kg | unknown | Lognormal |  | Unknown and hence excluded from the analysis |
| Heat | MJ | unknown | Lognormal |  | Unknown and hence excluded from the analysis |
| **Output** |  |  |  |  |  |
| Barium carbonate | kg | 1.00 |  |  |  |
| Sodium sulfide | kg | 0.40 |  |  |  |
| Carbon dioxide (fossil) | kg | 0.56 | Lognormal | 1.69 | (4,5,5,5,1,5) |
| Effluent | kg | unknown | Lognormal |  | Unknown and hence excluded from the analysis |

Step 3. Barium oxide production. The barium carbonate is transformed into barium oxide at high temperatures following the equation:

BaCO_3_ 🡪 BaO + CO_2_

The energy input to this process is approximated using the ecoinvent unit process “quicklime, in pieces, loose, at plant. A conversion efficiency of 100% is assumed.

Step 4. Barium metal production. The chemical reaction of industrial barium metal production is:

4BaO + 2 Al 🡪 BaAl_2_O_4_ + 3 Ba

The energy use of this step is approximated using the upgrading energy from calcium oxide to metallic calcium (19.5kWh/kg) and adjusting this by the ratio of the enthalpies of formation *Δ_f_H^0^_298_* of BaO and CaO. A conversion efficiency of 90% is assumed. Refining to metallic barium is undertaken mostly by only one producer (Chemetall GmbH) in Germany [63] and the electricity mix for Germany is used to model the energy requirement.

1. **Lanthanum (La, Z=57)**

The following rare earth elements are included: lanthanum (La), cerium (Ce), praseodymium (Pr), neodymium (Nd), samarium (Sm), europium (Eu), gadolinium (Gd), terbium (Tb), dysprosium (Dy), holmium (Ho), erbium (Er), thulium (Tm), ytterbium (Yb), lutetium (Lu), and yttrium (Y).

Environmental implications of the rare earth oxides are based on the ecoinvent entry ‘Rare earth concentrate, 70% REO, from bastnaesite, at beneficiation/CN U’ and subsequent rare earth separation [17]. This unit process is based on typical bastnaesite mining and refining in China. Rare earth production was primarily from bastnaesite mineral in 2008 with the major producer being China (97%) followed by India (2%), Brazil, Malaysia, and others (together 1%) [15]. We first unallocate the inventory for rare earth separation given in Ecoinvent 2.2. and then reallocate environmental burdens based on year 2006-2010 price averages from [64] and using rare earth element distribution in Bayan Obo bastnasite concentrate from [65,66] (Table S25). This is a simplification because it assumes that rare earths are recovered all at similar rates.

Furthermore, the vast majority of the global production for the heavy rare earth oxides (HREEs) (Tb, Dy, Ho, Er, Tm, Yb, and Lu) is obtained from ion absorption deposits [67], and the process used to extract and recover these HREEs and related environmental impacts are different from the process route described in Classen et al (2009) [13]. Recovery of HREEs from ion-absorption clays was not further investigated in this study and it is recommended to do so in future studies.

**Table S25** Calculation of the environmental impacts of rare earth oxide (REO) production.

| **A** | **B** | **D** | **E** | **F** | **G** | **H** |
| --- | --- | --- | --- | --- | --- | --- |
| **REOs** | **2006-2010 Price per kg** | **Bastnasite, Bayan Obo, Inner Mongolia** [68] | **Bastnasite, Bayan Obo, Inner Mongolia** [65] | **Bastnasite, Bayan Obo, Inner Mongolia** [66] | **Used in this study** | **Allocation %** |
| La_2_O_3_ | $ 35.60 | 23.00% | 25.00 | 21.32 | 25.00^1^ | 17.312% |
| CeO_2_ | $ 41.67 | 50.00% | 50.07 | 40.76 | 50.07^1^ | 40.577% |
| Pr_6_O_11_ | $ 62.08 | 6.20% | 5.10 | 4.22 | 5.10^1^ | 6.158% |
| Nd_2_O_3_ | $ 56.84 | 18.50% | 16.60 | 14.23 | 16.60^1^ | 18.352% |
| Sm_2_O_3_ | $ 191.19 | 0.80% | 1.20 | 1.03 | 1.20^1^ | 4.462% |
| Eu_2_O_3_ | $ 1,280.13 | 0.20% | 0.18 | 0.16 | 0.18^1^ | 4.482% |
| Gd_2_O_3_ | $ 151.02 | 0.70% | 0.70 | 0.61 | 0.70^1^ | 2.056% |
| Tb_4_O_7_ | $ 960.10 | 0.10% | <0.01 | 0.01 | 0.01^2^ | 0.187% |
| Dy2O3 | $ 191.92 | 0.10% | <0.01 | 0.02 | 0.02^2^ | 0.075% |
| Ho_2_O_3_ | $ 730.73 | trace | <0.01 | 0.03 | 0.03^2^ | 0.426% |
| Er_2_O_3_ | $ 157.29 | trace | <0.01 | 0.03 | 0.03^2^ | 0.092% |
| Tm_2_O_3_ | $ 2,102.10 | trace | <0.01 | 0.04 | 0.04^2^ | 1.635% |
| Yb_2_O_3_ | $ 404.04 | trace | <0.01 | 0.05 | 0.05^2^ | 0.393% |
| Lu_2_O_3_ | $ 2,900.29 | trace | <0.01 | 0.06 | 0.06^2^ | 3.385% |
| Y_2_O_3_ | $ 48.80 | trace | 0.43 | 0.34 | 0.43^1^ | 0.408% |
| **Total** | **-** | **99.60%** | **99.28** | **82.91** | **99.52** | **100%** |

^1^ Chinese Rare Earth Yearbook (2010) [65]

^2^ Du and Graedel (2011) [66]

1. **Cerium (Ce, Z=58)**

Please see entry for lanthanum.

1. **Praseodymium (Pr, Z=59)**

Please see entry for lanthanum.

1. **Neodymium (Nd, Z=60)**

Please see entry for lanthanum.

1. **Samarium (Sm, Z=62)**
2. Please see entry for lanthanum.
3. **Europium (Eu, Z=63)**

Please see entry for lanthanum.

1. **Gadolinium (Gd, Z=64)**

Please see entry for lanthanum.

1. **Terbium (Tb, Z=65)**

Please see entry for lanthanum.

1. **Dysprosium (Dy, Z=66)**

Please see entry for lanthanum.

1. **Holmium (Ho, Z=67)**

Please see entry for lanthanum.

1. **Erbium (Er, Z=68)**

Please see entry for lanthanum.

1. **Thulium (Tm, Z=69)**
2. Please see entry for lanthanum.
3. **Ytterbium (Yb, Z=70)**

Please see entry for lanthanum.

1. **Lutetium (Lu, Z=71)**

Please see entry for lanthanum.

1. **Hafnium (Hf, Z=72)**

Please see entry for zirconium.

1. **Tantalum (Ta, Z=73)**

The life cycle inventory for tantalum is based on the Ecoinvent entry “Tantalum powder, capacitor-grade, at regional storage/GLO U” [13] (see Table S38).

1. **Tungsten (W, Z=74)**

Life cycle inventory data for primary tungsten production is available from [34,42]. The estimate by [34] is largely based on energy inputs to the mining and refining stages, while [42] provides detailed cradle-to-gate estimates for emissions to air and water associated with tungsten production. However, estimates of cumulative energy use per kg of metal produced vary between 52.4 MJ/kg [42] and 214.5 MJ/kg [34]. The latter estimate is closer to embodied energy use reported elsewhere (329 MJ/kg [22] and 400 MJ/kg [69]. For this study, we report the average impact of both unit processes (i.e., [34,42]). Uncertainty of each parameter of the life cycle inventory is assessed using the Pedigree matrix [1,2]. The Ecoinvent process names chosen based on the inventory provided in ProBas is shown in Table S26.

Table S26 Life cycle inventory showing process names chosen in the Ecoinvent database for 1 metric ton of tungsten production. The inventory is based on data given in the ProBas database [42] and is publicly available at http://www.probas.umweltbundesamt.de.

| **Inputs** | **Amount** | **Unit** | **Distribution** | **StDv95%** | **Notes** |
| --- | --- | --- | --- | --- | --- |
| Biomass | 35.7 | kg | Lognormal | 1.27 | (2,3,3,5,1,5) |
| Occupation, arable | 43.3 | m2a | Lognormal | 1.6 | (2,3,3,5,1,5) |
| Occupation, industrial area | 2344 | m2a | Lognormal | 1.6 | (2,3,3,5,1,5) |
| Transformation, from unknown | 18.3 | m2 | Lognormal | 2.08 | (2,3,3,5,1,5) |
| Energy, potential (in hydropower reservoir), converted | 10165 | MJ | Lognormal | 1.27 | (2,3,3,5,1,5) |
| Energy, from coal | 32872 | MJ | Lognormal | 1.27 | (2,3,3,5,1,5) |
| Energy, from uranium | 9374 | MJ | Lognormal | 1.27 | (2,3,3,5,1,5) |
| Energy, unspecified | 0.503 | MJ | Lognormal | 1.27 | (2,3,3,5,1,5) |
| Water, cooling, drinking | 148327 | kg | Lognormal | 1.27 | (2,3,3,5,1,5) |
| Tungsten ore, in ground | 341 | ton | Lognormal | 1.27 | (2,3,3,5,1,5) |
| Metals n.e.c., extracted for use | 0.17 | ton | Lognormal | 1.27 | (2,3,3,5,1,5) |
| Metals, n.e.c., related unused extraction | 1.26 | ton | Lognormal | 1.27 | (2,3,3,5,1,5) |
| **Outputs** |  |  |  |  |  |
| Tungsten, metallic | 1 | ton | - | - | - |
| **Emissions to air** |  |  |  |  |  |
| Trichloroethane | 2.7E-09 | kg | Lognormal | 2.3 | (2,3,3,5,1,5) |
| Ethane, 1,2-dichloro- | 0.000222 | kg | Lognormal | 2.3 | (2,3,3,5,1,5) |
| Arsenic | 0.907 | kg | Lognormal | 5.09 | (2,3,3,5,1,5) |
| Benzo(a)pyrene | 0.0148 | kg | Lognormal | 3.08 | (2,3,3,5,1,5) |
| Benzene | 0.0148 | kg | Lognormal | 2.3 | (2,3,3,5,1,5) |
| Lead | 2.26 | kg | Lognormal | 5.09 | (2,3,3,5,1,5) |
| Cadmium | 0.317 | kg | Lognormal | 5.09 | (2,3,3,5,1,5) |
| Chlorinated fluorocarbons, soft | 0.0000696 | kg | Lognormal | 2.3 | (2,3,3,5,1,5) |
| Methane, biogenic | 0.0395 | kg | Lognormal | 1.6 | (2,3,3,5,1,5) |
| Methane | 4.38 | kg | Lognormal | 1.6 | (2,3,3,5,1,5) |
| Chromium | 0.0924 | kg | Lognormal | 5.09 | (2,3,3,5,1,5) |
| Carbon monoxide | 13.8 | kg | Lognormal | 5.09 | (2,3,3,5,1,5) |
| Carbon dioxide, fossil | 2554 | kg | Lognormal | 1.27 | (2,3,3,5,1,5) |
| Carbon dioxide, biogenic | 44.4 | kg | Lognormal | 1.27 | (2,3,3,5,1,5) |
| Methane, dichloro-, HCC-30 | 9.51E-08 | kg | Lognormal | 1.6 | (2,3,3,5,1,5) |
| Dioxins (unspec.) | 2.78E-09 | kg | Lognormal | 3.08 | (2,3,3,5,1,5) |
| Ethene | 0.00886 | kg | Lognormal | 2.3 | (2,3,3,5,1,5) |
| Particulates, < 10 um | 51.8 | kg | Lognormal | 3.08 | (2,3,3,5,1,5) |
| Formaldehyde | 0.00558 | kg | Lognormal | 2.3 | (2,3,3,5,1,5) |
| Ozone | 0 | kg | Lognormal | 2.3 | (2,3,3,5,1,5) |
| Hydrogen sulfide | 0.00804 | kg | Lognormal | 2.3 | (2,3,3,5,1,5) |
| Hydrogen chloride | 0.15 | kg | Lognormal | 2.3 | (2,3,3,5,1,5) |
| Phosphate | 0 | kg | Lognormal | 2.3 | (2,3,3,5,1,5) |
| Benzene, hexachloro- | 0.000000748 | kg | Lognormal | 3.08 | (2,3,3,5,1,5) |
| Hydrogen fluoride | 0.0244 | kg | Lognormal | 2.3 | (2,3,3,5,1,5) |
| Copper | 2.5 | kg | Lognormal | 2.3 | (2,3,3,5,1,5) |
| Dinitrogen monoxide | 0.586 | kg | Lognormal | 2.3 | (2,3,3,5,1,5) |
| Ammonia | 8.27 | kg | Lognormal | 1.6 | (2,3,3,5,1,5) |
| Nickel | 1.78 | kg | Lognormal | 2.3 | (2,3,3,5,1,5) |
| NMVOC, non-methane volatile organic compounds, unspecified origin | 18.5 | kg | Lognormal | 1.6 | (2,3,3,5,1,5) |
| Nitrogen oxides | 50.5 | kg | Lognormal | 2.3 | (2,3,3,5,1,5) |
| Polychlorinated biphenyls | 0.00000112 | kg | Lognormal | 3.08 | (2,3,3,5,1,5) |
| Anisole, pentachloro- | 0.00000807 | kg | Lognormal | 3.08 | (2,3,3,5,1,5) |
| Polycyclic organic matter, unspecified | 0.00102 | kg | Lognormal | 3.08 | (2,3,3,5,1,5) |
| Mercury | 0.002 | kg | Lognormal | 5.09 | (2,3,3,5,1,5) |
| Sulfur hexafluoride | 0.000108 | kg | Lognormal | 2.3 | (2,3,3,5,1,5) |
| Sulfur dioxide | 424 | kg | Lognormal | 2.3 | (2,3,3,5,1,5) |
| Zinc | 0.699 | kg | Lognormal | 2.3 | (2,3,3,5,1,5) |
| Methane, trichlorofluoro-, CFC-11 | 0.217 | kg | Lognormal | 1.6 | (2,3,3,5,1,5) |
| **Emissions to water** |  |  |  |  |  |
| Arsenic | 0.00235 | kg | Lognormal | 5.09 | (2,3,3,5,1,5) |
| Benzo(a)pyrene | 0 | kg | Lognormal | 3.08 | (2,3,3,5,1,5) |
| Benzene | 0.0137 | kg | Lognormal | 3.08 | (2,3,3,5,1,5) |
| Lead | 0.00218 | kg | Lognormal | 5.09 | (2,3,3,5,1,5) |
| BOD5, Biological Oxygen Demand | 7.29 | kg | Lognormal | 1.6 | (2,3,3,5,1,5) |
| Cadmium | 0.000215 | kg | Lognormal | 5.09 | (2,3,3,5,1,5) |
| Chloride | 13.5 | kg | Lognormal | 1.6 | (2,3,3,5,1,5) |
| Chromium | 0.0215 | kg | Lognormal | 5.09 | (2,3,3,5,1,5) |
| COD, Chemical Oxygen Demand | 12.9 | kg | Lognormal | 1.6 | (2,3,3,5,1,5) |
| Cyanide | 0.438 | kg | Lognormal | 1.6 | (2,3,3,5,1,5) |
| Fluoride | 0.13 | kg | Lognormal | 1.6 | (2,3,3,5,1,5) |
| CFCs, unspecified | 0.000148 | kg | Lognormal | 3.08 | (2,3,3,5,1,5) |
| Butadiene, hexachloro- | 0 | kg | Lognormal | 3.08 | (2,3,3,5,1,5) |
| Copper | 0.0432 | kg | Lognormal | 1.6 | (2,3,3,5,1,5) |
| Ammonia | 0.0361 | kg | Lognormal | 1.6 | (2,3,3,5,1,5) |
| Nickel | 0.856 | kg | Lognormal | 1.6 | (2,3,3,5,1,5) |
| Nitrate | 0.189 | kg | Lognormal | 1.6 | (2,3,3,5,1,5) |
| Tin | 0.0000139 | kg | Lognormal | 1.6 | (2,3,3,5,1,5) |
| TOC, Total Organic Carbon | 4.62 | kg | Lognormal | 1.6 | (2,3,3,5,1,5) |
| Phenol | 0.00134 | kg | Lognormal | 3.08 | (2,3,3,5,1,5) |
| Polychlorinated biphenyl, PCB-1254 | 0.0000979 | kg | Lognormal | 3.08 | (2,3,3,5,1,5) |
| Mercury | 0.0000373 | kg | Lognormal | 5.09 | (2,3,3,5,1,5) |
| Sulfate | 272 | kg | Lognormal | 1.6 | (2,3,3,5,1,5) |
| Phosphate | 0.0565 | kg | Lognormal | 1.6 | (2,3,3,5,1,5) |
| Nitrogen | 2.85 | kg | Lognormal | 1.6 | (2,3,3,5,1,5) |
| Zinc | 0.0162 | kg | Lognormal | 1.6 | (2,3,3,5,1,5) |

1. **Rhenium (Re, Z=75)**

Rhenium is recovered primarily from the roasting of molybdenite (MoS_2_) from porphyry copper but today includes rhenium from copper deposits [70]. Rhenium is present in molybdenite ores (MoS_2_) at concentrations of 0.001-0.2% rhenium, with a value of 0.1% used here [60]. The production of 1kg of molybdenum requires 1.8 kg of molybdenite inputs into the roasting process [13]. The roasting process itself is assumed here to require 0.34 MJ of heat per kg of feed from heavy fuel oil [60], using the ecoinvent entry “Heat heavy fuel oil, at industrial furnace 1MW/RER U”. The rhenium recovery from Mo roaster flue dust equals 80% [70].

We model the roasting process using global average molybdenite inputs from [4] in SimaPro8 and allocate environmental burdens between molybdenum and rhenium (Table S27) based on 2006-2010 price averages (see Table S38).

Table S27 Allocation factors used for rhenium production as a by-product from molybdenum roasting.

| Product | kg | US$/kg^1^ | Allocation Percentage |
| --- | --- | --- | --- |
| Molybdenum, at regional storage/RER U, reallocated | 1 | $ 49.03 | 86.5% |
| Rhenium, at plant, co-product of Mo roasting | 0.00147 | $ 5,216.00 | 13.5% |

^1^2006-2010 price averages as reported in USGS Mineral Commodity Summaries [32].

1. **Osmium (Os, Z=76)**

Please see entry for platinum.

1. **Iridium (Ir, Z=77)**

Please see entry for platinum.

1. **Platinum (Pt, Z=78)**

Environmental burdens of the platinum group metals (PGMs = platinum, palladium, rhodium, ruthenkum, iridium, and osmium) are based on the life cycle inventories in the ecoinvent database [13]. Ecoinvent provides data for the elements platinum, palladium, and rhodium (but not ruthenium, iridium, and osmium) mined together with copper and nickel in South Africa (Bushveld mine, PGM dominated) and Russia (Noril’sk mine, Cu-Ni dominated). In the database, the split of environmental burdens is based on price data for years 1993-2002 assuming that the ratio of PGM produced corresponds to ratios in the raw ores [13]. The allocation of mass is solely used for the allocation for PGM-ores to the respective metal products.

For our assessment, LCI datasets given in [13] were first unallocated and then reallocated using 2006-2010 price averages and production data for South Africa and Russia, for the three metals mentioned above, as well as ruthenium, iridium, and osmium [71]. Production data is chosen as it may provide a better estimate of the quantity of PGMs recovered than simply using elemental ore compositions. However, as shown in Table S28, production data for all six elements are only available for years from 1970 to 1992^[[5]](#footnote-5)^. The majority of other public sources only report PGM production for platinum, palladium, and rhodium (see e.g., [72,73]). While for this assessment the historical production data until 1992 are used for mass allocation, Table S29 shows ore distributions of the PGMs for South Africa and Russia that may be used instead. In our assessment, the quantities of copper and nickel co-produced are derived using the production grades from [74], according to which 106 kg copper and 229 kg nickel, and 3738 kg copper and 1834 kg nickel are produced per kg of PGM in South Africa and Russia, respectively.

Table S28 Production from year 1970 to 1992 by platinum grade metal (PGM) based on [71].

| **South Africa (kg)** | **1970-1974** | **1975-79** | **1980-84** | **1985-89** | **1990-92** | **Allocation by mass (ZA)** | **PGM only** |
| --- | --- | --- | --- | --- | --- | --- | --- |
| Pt | 35,462 | 55,147 | 58,223 | 77,606 | 87,944 | 0.182% | 61.01% |
| Pd | 15,169 | 23,083 | 26,053 | 33,736 | 40,357 | 0.080% | 26.86% |
| Ru | 4,852 | 6,963 | 7,353 | 10,131 | 10,412 | 0.023% | 7.71% |
| Rh | 1,812 | 2,604 | 2,276 | 4,290 | 5,770 | 0.010% | 3.25% |
| Ir | 636 | 911 | 762 | 1,020 | 1,048 | 0.003% | 0.85% |
| Os | 292 | 418 | 272 | 347 | 357 | 0.001% | 0.33% |
| **PGM** | **58,223** | **89,126** | **94,939** | **127,130** | **145,888** |  |  |
| Cu | 6,161,164 | 9,431,323 | 10,046,455 | 13,452,910 | 15,437,884 | 31.5% |  |
| Ni | 13,349,189 | 20,434,533 | 21,767,319 | 29,147,972 | 33,448,748 | 68.2% |  |
| **TOTAL** | **19,568,576** | **29,954,981** | **31,908,713** | **42,728,012** | **49,032,519** | **100.0%** |  |
|  |  |  |  |  |  |  |  |
| **Russia (kg)** | **1970-1974** | **1975-79** | **1980-84** | **1985-89** | **1990-92** | **Allocation by mass (RU)** | **PGM only** |
| Pt | NA | NA | 25,605 | 29,249 | 30,376 | 0.004% | 24.73% |
| Pd | NA | NA | 73,738 | 85,612 | 83,629 | 0.012% | 68.09% |
| Ru | NA | NA | 1,971 | 2,013 | 2,061 | 0.0003% | 1.68% |
| Rh | NA | NA | 3,087 | 3,090 | 3,206 | 0.0005% | 2.61% |
| Ir | NA | NA | 1,961 | 2,160 | 2,452 | 0.0003% | 2.00% |
| Os | NA | NA | 1,014 | 1,028 | 1,101 | 0.0002% | 0.90% |
| **PGM** | **NA** | **NA** | **107,376** | **123,152** | **122,825** |  |  |
| Cu | NA | NA | 401,415,612 | 460,392,782 | 459,170,322 | 67.1% |  |
| Ni | NA | NA | 196,961,260 | 225,899,392 | 225,299,571 | 32.9% |  |
| **TOTAL** | **NA** | **NA** | **598,484,248** | **686,415,326** | **684,592,718** | **100.0%** |  |

NA: Not available

Table S29 Ore distribution of platinum grade metals (PGMs) in South Africa and Russia based on various literature sources.

| **Country** | **South Africa** | | | | | **Russia** | | |
| --- | --- | --- | --- | --- | --- | --- | --- | --- |
| **Mine** | Merensky | Merensky | Bushveld | Bushveld | Bushveld^1^ | Noril'sk | Noril'sk | Noril'sk^1^ |
| **Source** | [75] | [13] | [75] | [13] | [74] | [75] | [13] | [74] |
| **Pt** | 61.0% | 63.1% | 47.0% | 40.8% | 51.1% | 25.0% | 25.0% | 18.9% |
| **Pd** | 26.0% | 26.7% | 32.0% | 41.0% | 32.1% | 67.0% | 73.0% | 76.8% |
| **Rh** | 3.0% | 3.1% | 7.0% | 6.8% | 5.5% | 3.0% | 2.0% | 2.3% |
| **Ru** | 8.0% | 5.8% | 11.0% | 9.1% | 9.6% | 2.0% | NA | 1.2% |
| **Ir** | 1.0% | 0.8% | 2.0% | 1.4% | 1.8% | 2.0% | NA | 0.3% |
| **Os** | 1.0% | 0.5% | 1.0% | 0.9% | 1.0% | 1.0% | NA | 0.5% |

^1^Used in this study. NA = not available

Secondary production of platinum, palladium and rhodium (from auto catalysts) is based on [13] and environmentally relevant flows reallocated using 2006-2010 price averages. Allocation percentages based on revenue and how they compare to previous allocation percentages given in Ecoinvent are shown in the following tables.

Table S30 Reallocation of environmental burdens of the platinum grade metals (PGMs).

|  | **Allocation by mass (ZA)^1^** | **Allocation by mass (RU)^1^** | **Allocation by mass (Secondary) RER)** | **Allocation by revenue (ZA)** | **Allocation by revenue (RU)** | **Allocation by Revenue (secondary) RER** | **Global 5-year average price (2006-2010) (US$/kg)** | **Previous Allocation (ZA) - Ecoinvent** | **Previous Allocation (RU) - Ecoinvent** |
| --- | --- | --- | --- | --- | --- | --- | --- | --- | --- |
| **Pt** | 0.182% | 0.004% | 53% | **58.30%** | **12.25%** | **44.37%** | $ 43,627.17 | 66.0% | 11.0% |
| **Pd** | 0.080% | 0.012% | 26% | **7.25%** | **9.53%** | **6.15%** | $ 12,324.69 | 19.0% | 21.0% |
| **Rh** | 0.023% | 0.0003% | 21% | **20.73%** | **2.34%** | **49.48%** | $ 122,800.98 | 7.0% | 2.0% |
| **Ru** | 0.010% | 0.0005% |  | **0.41%** | **0.17%** | 0.00% | $ 5,774.06 | 0.0% | 0.0% |
| **Ir** | 0.003% | 0.0004% |  | **0.43%** | **0.53%** | 0.00% | $ 23,366.63 | 0.0% | 0.0% |
| **Os** | 0.001% | 0.0002% |  | **0.08%** | **0.12%** | 0.00% | $ 11,654.65 | 0.0% | 0.0% |
| **Cu** | 31.485% | 67.072% |  | **1.55%** | **28.42%** | 0.00% | $ 6.70 | 1.0% | 19.0% |
| **Ni** | 68.217% | 32.910% |  | **11.25%** | **46.64%** | 0.00% | $ 22.40 | 7.0% | 47.0% |

^1^Based on 1970-1992 PGM production statistics as reported in [71] and copper and nickel co-product quantities based on [74].

1. **Gold (Au, Z=79)**

The environmental burdens of gold are derived using data of primary gold (from gold ore), primary gold (from gold-silver production), and secondary gold (at precious metals refinery) [13] (see Table S38).

1. **Mercury (Hg, Z=80)**

Mercury production is either from its own cinnabar ore (HgS), as a byproduct of precious metals processing (gold, silver, tin and zinc mines), or from recycling of industrial waste materials [76]. The main producer of mercury in 2008 was China, followed by Kyrgyztan and Peru [15]. However, production estimates are uncertain due to fact that many countries do not properly report production statistics as a result of the toxic implications of mercury provision.

In the U.S., currently mercury is produced only as a byproduct of domestic gold-silver processing [15]. In 2000, global mercury production per route was approximately 68.5% (from its own ore), 30.2% (as by-product and recycling) and 1.4% (artisanal mining) [77]. Data on the amount of by-product mercury produced was not available. Therefore, we use inventory data from Ecoinvent developed for Hg from its own cinnabar ore as proxy of the environmental burdens [13].

1. **Thallium (Tl, Z=81)**

Although the amounts of thallium in lead and zinc ores are small in comparison to those in salts and other rocks, most of the thallium produced worldwide comes from the former [78]. Data on global thallium production from zinc ores is based on a life cycle inventory given in the ProBas database [42,79] (Table S31). ProBas gives information about the cumulative resource and energy use, as well as emissions to air and water. In the eco-profile, allocation between zinc, thallium, indium and other by-products obtained during mining is based on average metal prices between years 1978 to 1998 given by [80]. Information given is not detailed enough to allow unallocation of environmental burdens and reallocation using 2006-2010 price averages. Hence, the existing dataset was used.

Table S31 Life cycle inventory showing process names chosen in the Ecoinvent database for 1 metric ton of thallium production. The inventory is based on data given in the ProBas database [42] and is publicly available at http://www.probas.umweltbundesamt.de.

| **Input** | **Unit** | **Amount** | **Distribution** | **StDv95%** | **Notes** |
| --- | --- | --- | --- | --- | --- |
| **Resources** |  |  |  |  |  |
| Biomass | kg | 675 | Lognormal | 1.31 | (2,5,1,3,1,5) |
| Occupation, arable | m2a | 512 | Lognormal | 1.62 | (2,5,1,3,1,5) |
| Occupation, industrial area | m2a | 8529 | Lognormal | 1.62 | (2,5,1,3,1,5) |
| Transformation, from unknown | m2 | 150 | Lognormal | 1.31 | (2,5,1,3,1,5) |
| Energy, from biomass | MJ | 733377 | Lognormal | 1.31 | (2,5,1,3,1,5) |
| Energy, from coal | MJ | 3603932 | Lognormal | 1.31 | (2,5,1,3,1,5) |
| Energy, from uranium | MJ | 824518 | Lognormal | 1.31 | (2,5,1,3,1,5) |
| Energy, unspecified | MJ | 7.37 | Lognormal | 1.31 | (2,5,1,3,1,5) |
| Iron | ton | 459 | Lognormal | 1.31 | (2,5,1,3,1,5) |
| Water, cooling, drinking | kg | 8491961 | Lognormal | 1.31 | (2,5,1,3,1,5) |
| **Output** |  |  |  |  |  |
| Thallium (ProBas) | ton | 1 | - | - | - |
| **Emissions to air** |  |  |  |  |  |
| Trichloroethane | kg | 0.0000451 | Lognormal | 2.1 | (2,5,1,3,1,5) |
| Arsenic | kg | 1.32 | Lognormal | 1.62 | (2,5,1,3,1,5) |
| Benzo(a)pyrene | kg | 1.68 | Lognormal | 3.09 | (2,5,1,3,1,5) |
| Benzene | kg | 1.68 | Lognormal | 3.09 | (2,5,1,3,1,5) |
| Lead | kg | 15.6 | Lognormal | 1.62 | (2,5,1,3,1,5) |
| Cadmium | kg | 0.104 | Lognormal | 1.62 | (2,5,1,3,1,5) |
| Chlorinated fluorocarbons, soft | kg | 0.00559 | Lognormal | 1.62 | (2,5,1,3,1,5) |
| Ozone | kg | 165 | Lognormal | 1.62 | (2,5,1,3,1,5) |
| Phosphate | kg | 268 | Lognormal | 1.62 | (2,5,1,3,1,5) |
| Carbon dioxide, fossil | kg | 309414 | Lognormal | 1.31 | (2,5,1,3,1,5 |
| Sulfur dioxide | kg | 4400 | Lognormal | 1.62 | (2,5,1,3,1,5) |
| Methane, trichlorofluoro-, CFC-11 | kg | 14 | Lognormal | 1.62 | (2,5,1,3,1,5) |
|  |  |  |  |  |  |
| **Emissions to water** |  |  |  |  |  |
| Arsenic | kg | 0.325 | Lognormal | 5.11 | (2,5,1,3,1,5) |
| Benzo(a)pyrene | kg | 0 | Lognormal | 3.09 | (2,5,1,3,1,5) |
| Benzene | kg | 0.471 | Lognormal | 3.09 | (2,5,1,3,1,5) |
| Lead | kg | 4.41 | Lognormal | 5.11 | (2,5,1,3,1,5) |
| BOD5, Biological Oxygen Demand | kg | 217 | Lognormal | 1.62 | (2,5,1,3,1,5) |
| Cadmium | kg | 0.384 | Lognormal | 5.11 | (2,5,1,3,1,5) |
| Chloride | kg | 2295 | Lognormal | 1.62 | (2,5,1,3,1,5) |
| Chromium | kg | 0.185 | Lognormal | 5.11 | (2,5,1,3,1,5) |
| COD, Chemical Oxygen Demand | kg | 272 | Lognormal | 1.62 | (2,5,1,3,1,5) |
| Cyanide | kg | 3.64 | Lognormal | 1.62 | (2,5,1,3,1,5) |
| Fluoride | kg | 5.02 | Lognormal | 1.62 | (2,5,1,3,1,5) |
| CFCs, unspecified | kg | 0.00395 | Lognormal | 1.62 | (2,5,1,3,1,5) |
| Butadiene, hexachloro- | kg | 0 | Lognormal | 2.12 | (2,5,1,3,1,5) |
| Copper | kg | 0.608 | Lognormal | 1.62 | (2,5,1,3,1,5) |
| Ammonia | kg | 1.2 | Lognormal | 1.62 | (2,5,1,3,1,5) |
| Nickel | kg | 0.355 | Lognormal | 1.62 | (2,5,1,3,1,5) |
| Nitrate | kg | 5.39 | Lognormal | 1.62 | (2,5,1,3,1,5) |
| Tin | kg | 0.00159 | Lognormal | 1.62 | (2,5,1,3,1,5) |
| TOC, Total Organic Carbon | kg | 78.3 | Lognormal | 1.62 | (2,5,1,3,1,5) |
| Phenol | kg | 0.0745 | Lognormal | 3.09 | (2,5,1,3,1,5) |
| Polychlorinated biphenyl, PCB-1254 | kg | 0.00502 | Lognormal | 3.09 | (2,5,1,3,1,5) |
| Mercury | kg | 0.0182 | Lognormal | 5.11 | (2,5,1,3,1,5) |
| Sulfate | kg | 5401 | Lognormal | 1.62 | (2,5,1,3,1,5) |
| Phosphate | kg | 1.1 | Lognormal | 1.62 | (2,5,1,3,1,5) |
| Phosphate | kg | 30.4 | Lognormal | 1.62 | (2,5,1,3,1,5) |

1. **Lead (Pb, Z=82)**

See also the entry for germanium. The Pb environmental burdens are derived using data of primary lead (from lead/zinc ores), secondary lead, and secondary lead (from electronic and electric scrap) [13] (see Table S38).

1. **Bismuth (Bi, Z=83)**

World production of bismuth stems mainly from processing of lead and copper production, of which the metal is a by-product [81]. According to [82], the main commercial sources of bismuth (90-95%) are waste products from lead refining. Some bismuth is also mined as a by-product associated with tungsten, tin, and molybdenum. In 2008, China was the world’s leading producer of refined bismuth with roughly 78% of the world total refinery production, followed by Mexico (8%), Belgium (5%), and others (9%) [15]. Production in China is from lead but also as an accompanying element of tungsten, tin, and fluorite [82].

Bismuth is not included in Ecoinvent [4] and we use inventory data and uncertainty ranges for each parameter from Andrae et al. (2008) [83] to model the environmental impacts of metallic bismuth provision. Their data is mostly based on publically available Canadian company sustainability reports, where most of the bismuth is obtained as a by-product from lead-zinc mining [82]. The final product mix is given in Table S32. We update market prices (and hence allocation percentages) used by Andrae et al. (2008) [83] to 5-year (2006-2010) price averages based on USGS statistics [84].

Table S32 Bismuth by-production based on Canadian sustainability report [83].

|  |  | **Allocation** [83] | | **Allocation (this study)** | |
| --- | --- | --- | --- | --- | --- |
| **Product** | **Amount ( metric tons)** | **Allocation (%)** | **Price ($/kg)** | **Allocation (%)** | **Price ($/kg)** |
| Lead | 84,300 | 13.67 | 0.89 | 14.81 | 2.26 |
| Zinc | 296,000 | 56.71 | 1.05 | 59.06 | 2.57 |
| Silver | 590.9661 | 23.25 | 215.09 | 22.11 | 481.36 |
| Bismuth | 18 | 0.02 | 7.10 | 0.03 | 21.12 |
| Indium | 30 | 3.48 | 634.00 | 1.62 | 692.60 |
| Germanium | 26.1 | 2.86 | 600.00 | 2.38 | 1172.00 |

In Andrae et al. (2008) [83], electricity used during the refining step is modeled to come from the Canadian power grid. Instead, in order to adapt the assessment to the world average, we use a weighted average electricity input for the three major refining countries (China, Mexico, Belgium) using 2008 fuels shares from World Bank statistics [25] and linking those to respective Ecoinvent electricity production unit processes in SimaPro. The remainder of power inputs (other countries) is approximated using a UCTE average power mix^[[6]](#footnote-6)^. Impacts to GWP and human health are largely governed by electricity inputs from coal-fired power plants in China (78% of world refining production [15] with an electricity mix using 79% coal in 2008 [25]). In contrast, the Canadian power mix relies largely on hydropower (58%) [83] with much lower contributions to CED.

1. **Thorium (Th, Z=90)**

Please note: This inventory is limited by data availability and should only be seen as a first guess estimate of environmental burdens associated with thorium oxide production. It is based on rare earth mining from monazite and refining of the concentrate into rare earth elements. Thorium is separated from rare earths via precipitation as thorium phosphate at a low pH [85] and allocation based on the economic value of product outputs.

Thorium is almost always extracted as a companion metal with the rare earths [85]. The most important mineral for thorium production is monazite [64]. Monazite itself is obtained as a byproduct from the processing of heavy mineral sands, mostly from the extraction of zircon, ilmenite and rutile [86]. The majority of monazite concentrate in 2008 was produced by India (77%), followed by Brazil (19%), and Malaysia (4%) [26]. Much of this comes from coastal placer deposits. After mining and concentration, typical thorium oxide content in monazite ranges from about 3-9 % (wt), with rare earth oxide concentrations being around 40-60% and the remainder consisting mainly of phosphorus oxides [85]. Further ore digestion with acid, water leaching and purification, and separation of the individual metals by solvent extraction yields thorium oxide and rare earth oxides as final products [85,87]. The composition of monazite concentrates used in this study is shown in Table S33 and Table S34.

Table S33 Composition of monazite concentrates [85].

| **Constituent** | **India** | **Brazil** | **Malaysia^2^** | **Florida beach sand** | **South Africa** | **Used in this study^3^** |
| --- | --- | --- | --- | --- | --- | --- |
| **ThO_2_** | 8.88 | 6.5 | 8.75 | 3.1 | 5.9 | **8.42** |
| **U_3_O_8_** | 0.35 | 0.17 | 0.41 | 0.47 | 0.12 | 0.32 |
| **(RE)_2_O_3_^1^** | 59.37 | 59.2 | 46.2 | 40.7 | 46.41 | **58.81** |
| **P_2_O_5_** | 27.03 | 26 | 20 | 19.3 | 27 | 26.55 |
| **Fe_2_O_3_** | 0.32 | 0.51 |  | 4.47 | 4.5 | 0.34 |
| **TiO_2_** | 0.36 | 1.75 | 2.2 |  | 0.42 | 0.70 |
| **SiO_2_** | 1 | 2.2 | 6.7 | 8.3 | 3.3 | 1.46 |

^1^Rare earth oxides with composition shown in Table S34. ^2^The ore originates mostly from Mount Weld in Australia and is being shipped to Malaysia for further processing. ^3^Production-weighted average of the composition of concentrates from India, Brazil and Malaysia in year 2008.

Table S34 Rare earth distribution in monazite from the three major mining locations [68].

| **Rare earth** | **India** | **Brazil, East coast** | **Australia, Mount Weld^1^** | **Used in this study^2^** |
| --- | --- | --- | --- | --- |
| **La_2_O_3_** | 23.00 | 24.00 | 26.00 | 23.31 |
| **CeO_2_** | 46.00 | 47.00 | 51.00 | 46.39 |
| **Pr_6_O_11_** | 5.50 | 4.50 | 4.00 | 5.25 |
| **Nd_2_O_3_** | 20.00 | 18.50 | 15.00 | 19.52 |
| **Sm_2_O_3_** | 4.0 | 3.00 | 1.8 | 3.72 |
| **Eu_2_O_3_** |  | 0.0550 | 0.4 | 0.03 |
| **Gd_2_O_3_** |  | 1.00 | 1.0 | 0.23 |
| **Tb_4_O_7_** |  | 0.1 | 0.1 | 0.02 |
| **Dy2O3** |  | 0.35 | 0.2 | 0.07 |
| **Ho_2_O_3_** |  | 0.035 | 0.1 | 0.01 |
| **Er_2_O_3_** |  | 0.07 | 0.2 | 0.02 |
| **Tm_2_O_3_** |  | 0.005 | Trace | trace |
| **Yb_2_O_3_** |  | 0.02 | 0.1 | 0.01 |
| **Lu_2_O_3_** |  |  | Trace | trace |
| **Y_2_O_3_** | Eu-Y: 1.50 | 1.4 | Trace | trace |

^1^The ore originates from Australia but is transformed into a concentrate in Malaysia. ^2^Production-weighted average of India, Brazil and Australia (Malaysia) in year 2008.

The process of monazite mining and beneficiation of rare earth oxides and thorium consists of the following steps:

1. **Mining and processing of mineral sands**: During this process, heavy mineral sands consisting of ilmenite and rutile (both titanium dioxide), zircon (which also contains hafnium), and monazite, are processed. The life cycle inventory is based on the Ecoinvent unit process “Zircon, 50% zirconium, at plant, AU/U” which represents typical mineral sands processing in Australia [4,17]. The existing inventory is expanded by including monazite at a concentration of 0.1% in the ore [17] and a 2006-2010 price of 0.84$/kg [84] as beneficial by-product. The inventory is based on a heavy mineral concentration of 6.1% (i.e., 16.39 kg of ore body is removed for each kg of heavy mineral concentrate) and yield of 95%. A weighted global average rare earth concentration in the crude monazite of 13.8% is used [13,68,86,87]. The resulting allocation percentages for all product outputs are shown in the table below.

Table S35 Product outputs from the processing of heavy mineral sands.

| **Output** | kg | 2006-2010 Price ($/kg) | Allocation Percentage |
| --- | --- | --- | --- |
| **Zircon, 50% zirconium, at plant** | 0.128 | $ 0.80 | **23%** |
| **Ilmenite, 54% titanium dioxide, at plant** | 0.789 | $ 0.37 | **66%** |
| **Rutile, 95% titanium dioxide, at plant** | 0.069 | $ 0.52 | **8%** |
| **Monazite, 100% (13.83%REO and 0.21% ThO2)^a^** | 0.016 | $ 0.84 | **3%** |

^a^ The rare earth composition of the crude monazite is given by Table S34. In this assessment the market price from the USGS [84] is used to allocate environmental burdens to the monazite.

1. **Monazite mineral concentration by flotation**: During this process step, the monazite is concentrated up to a rare earth oxide (REO) concentration of 59% and thorium content of 8.4%. Due to a lack of more detailed information, data on process energy use for mining and beneficiation, treatment chemicals use, water resources, and emissions to air, soil and water, from the ecoinvent process ‘rare earth concentrate from basnasite, at mine’ is used [13]. A recovery rate of 60 percent is used [13]. In order to obtain 1 kg of rare earth concentrate from monazite with a REO concentration of 59% and thorium content of 8.4%, 8.1 kg of crude monazite is required.
2. **“Cracking” of minerals followed by leaching and purification**: Subsequent rare earth separation and recovery is based on the material balance given in Schmidt (2013) [87] (Table S36). In order to generate 1 kg of REOs mixture and thorium, 2.1 kg of rare earth concentrate (59% REOs and 8.4% thorium oxide) is required. The process generates solid wastes (three types as shown in Table S36), which are treated and stored on site. No information on toxic trace elements such as cadmium, lead, arsenic, etc., is given in the report by Schmidt (2013) [87] and waste treatment not included in our LCI. Waste solvents generated are treated via the Ecoinvent process “Disposal, solvents mixture, 16.5% water, to hazardous waste incineration” [4]. Emissions to air include radon-220 (181.4 MBq/kg), sulfur dioxide (0.05 g/Nm3 flue gas from cracking), hydrogen fluoride (0.05 g/Nm3), and particulate matter (0.1g/Nm3) [87]. Off-gas generated equals 4.42 Nm3/kg REO mixture [87]. Environmental burdens are allocated between the rare earths and thorium dioxide based on 2006-2010 average prices [26] as shown in Table S37.

Table S36 Life cycle inventory for LAMP plant producing rare earth oxides (REOs) and thorium oxide from monazite in Malaysia. Data comes from Schmidt (2013) [87].

| **Input** | **Unit** | **Amount** | **Distribution** | **StDV95%** | **Notes** |
| --- | --- | --- | --- | --- | --- |
| **Input** |  |  |  |  |  |
| Rare earth concentrate, 58.5% REOs, 8.42% ThO2, from monazite, at beneficiation, GLO U | kg | 2.10 | Lognormal | 1.24 | Adjusted by REE percentage in Australian ore |
| Raw water |  | unknown |  |  | - |
| Natural gas, burned in industrial furnace >100kW/RER U | kg | 1.91 | Lognormal | 1.24 | (2,4,1,3,1,5) 53.2 MJ/kg |
| Sulphuric acid, liquid, at plant/RER U | kg | 4.90 | Lognormal | 1.24 | (2,4,1,3,1,5) concentrated 98% |
| Hydrochloric acid, 30% in H2O, at plant/RER U | kg | 6.52 | Lognormal | 1.24 | (2,4,1,3,1,5) concentrated 36% |
| Magnesium oxide, at plant/RER U | kg | 1.04 | Lognormal | 1.24 | (2,4,1,3,1,5) |
| Soda, powder, at plant/RER U | kg | 0.87 | Lognormal | 1.24 | (2,4,1,3,1,5) |
| Lime, hydrated, packed, at plant/CH U | kg | 2.32 | Lognormal | 1.24 | (2,4,1,3,1,5) |
| Acetic acid from acetaldehyde, at plant/RER U | kg | 0.40 | Lognormal | 1.24 | (2,4,1,3,1,5) Proxy for oxalic acid |
| Chemicals organic, at plant/GLO U | kg | 0.03 | Lognormal | 1.24 | (2,4,1,3,1,5) Proxy for solvents |
| Kerosene, at refinery/RER U | kg | 0.01 | Lognormal | 1.24 | (2,4,1,3,1,5) |
| Chemical plant, organic | unit | 8.00E-10 | Lognormal | 3.06 | (2,4,1,3,1,5) Proxy for infrastructure requirements |
| **Output** |  |  |  |  |  |
| *REO mixture, monazite, at plant (0.87 kg REOs, 0.13 kg ThO2)* | *kg* | *1.00* |  |  | *REOs* |
| Process water | kg | unknown |  |  | Process water |
| Off-gas | Nm3 | 4.42 |  |  |  |
| Radon-220 | mBq | 181.40 | Lognormal | 3.06 | (2,4,1,3,1,5) Radon-220 MBq |
| Sulfur dioxide, to air | kg | 2.208E-04 | Lognormal | 1.24 | (2,4,1,3,1,5) 99,344 Nm3/yr, 0.05g SO2/Nm3 |
| Hydrogen fluoride, to air | kg | 2.208E-04 | Lognormal | 1.24 | (2,4,1,3,1,5) 99,344 Nm3/yr, 0.05g Hf/Nm3 |
| Particulate matter (PM10), to air | kg | 4.415E-04 | Lognormal | 2.06 | (2,4,1,3,1,5) 99,344 Nm3/yr, 0.1g PM10/Nm3 |
| Water Leach Purification (WLP) waste | kg | 2.84 |  |  | WLP waste, dry base, stored on site and not accounted for in LCI |
| Flue Gas Desulfurization (FGD) waste | kg | 2.48 |  |  | FGD waste, dry base, stored on site and not accounted for in LCI |
| Neutralization Underflow Residue (NUF) waste | kg | 7.58 |  |  | NUF waste, dry base, stored on site and not accounted for in LCI |
| Disposal, solvents mixture, 16.5% waste, to hazardous waste incieration | kg | 0.03 | Lognormal | 1.24 | Solvent. The process "disposal, solvents mixture to hazardous waste incieration" is used as proxy of the treatment process. |
| Disposal, solvents mixture, 16.5% waste, to hazardous waste incieration | kg | 0.01 | Lognormal | 1.24 | Kerosene. The process "disposal, solvents mixture to hazardous waste incieration" is used as proxy of the treatment process. |

Uncertainty of each parameter of the life cycle inventory is assessed using the Pedigree matrix [1,2]. Finally, the distribution of rare earth elements and thorium, and their respective allocation percentages are shown in the following table.

Table S37 Distribution of rare earths and thorium in the final mixture obtained from monazite and their respective allocation percentages using 2006-2010 price data.

| **Product** | **Amount (kg)** | **20062-2010 Price ($/kg)^a^** | **Allocation %** |
| --- | --- | --- | --- |
| La2O3 | 2.039E-01 | $ 35.60 | 9.9415% |
| CeO2 | 4.058E-01 | $ 41.67 | 23.1565% |
| Pr6O11 | 4.592E-02 | $ 62.08 | 3.9048% |
| Nd2O3 | 1.707E-01 | $ 56.84 | 13.2892% |
| Sm2O3 | 3.256E-02 | $ 191.19 | 8.5252% |
| Eu2O3 | 2.314E-04 | $ 1,280.00 | 0.4056% |
| Gd2O3 | 2.012E-03 | $ 151.00 | 0.4161% |
| Tb4O7 | 2.012E-04 | $ 960.00 | 0.2645% |
| Dy2O3 | 6.517E-04 | $ 191.92 | 0.1713% |
| Ho2O3 | 9.316E-05 | $ 730.73 | 0.0932% |
| Er2O3 | 1.863E-04 | $ 157.29 | 0.0401% |
| Tm2O3 | 4.374E-05 | $ 2,102.10 | 0.1259% |
| Yb2O3 | 6.823E-05 | $ 404.04 | 0.0378% |
| Lu2O3 | 0.000E+00 | $ 2,900.00 | 0.0000% |
| Y2O3 | 1.225E-02 | $ 48.80 | 0.8185% |
| ThO2 | 0.1253 | $ 226.20 | 38.8098% |
| **Total** | **0.9999** | **$ 9,539.47** | **100%** |

^a^Based on [26].

1. **Uranium (U, Z=92)**

Inventory data on uranium production and refining was available from Ecoinvent [4]. We use the ecoinvent entry ‘Uranium, natural, in yellowcake, at mill plant/RNA U’ to model the environmental implications of uranium oxide production.

1. **Summary**

The following table contains a summary of the GWP and CED for each element and all use-forms considered in this study. It also includes uncertainty ranges (95% Stdv) and global production data for year 2008 used in this study to scale up environmental burdens.

Table S38 Summary table of metal environmental implications

| **Z** | **Material** | **Ore** | **Co-mined** | **2006-2010 Price Average**  **[US$/kg]** | **Price Data: Che-mical Form** | **Price Reference** | **Process Name** | **Data Origin** | **2008 Global Production [kg]** | **2008 Weighting [% of Supply]** | **Weighting Reference** | **CED [MJ/kg]** | **CED - 2.5% [MJ/kg]** | **CED - 97.5% [MJ/kg]** | **Global Cumulative Energy Demand (2008) [PJ/yr]** | **GWP 100a**  **[kg CO2-eq/ kg]** | **GWP - 2.5% [kg CO2-eq/ kg]** | **GWP - 97.5% [kg CO2-eq/ kg]** | **Global CO_2_ Emissions (2008) [kg CO2-eq/yr]** |
| --- | --- | --- | --- | --- | --- | --- | --- | --- | --- | --- | --- | --- | --- | --- | --- | --- | --- | --- | --- |
| 2 | He | Natural gas | Natural gas | Allocation based on energy content | Gas | Sutter (2007) [5] | Helium, at plant/GLO U | Ecoinvent 2.2 [4] | 2.975E+07 | 100% |  | 67.5 | 41.4 | 103 | 2.0 | 0.9 | 0.6 | 1.4 | 2.77E+07 |
|  | **He TOTAL** |  |  |  |  |  |  | **Ecoinvent 2.2** [4] | **2.975E+07** | **100%** |  | **67.5** | **41.4** | **103** | **2.0** | **0.9** | **0.6** | **1.4** | **2.77E+07** |
| 3 | Li | Lithium brine (Chile) | None | NA | Metal |  | Lithium, at plant/GLO U | Ecoinvent 2.2 [4] | - | 22% | Gruber et al (2011) [7] | 415 | 305 | 548 | - | 21.1 | 15.9 | 26.8 | - |
|  | Li_2_CO3 | Lithium brine (Chile) | None | NA | Carbonate |  | Lithium carbonate, at plant/GLO U | Ecoinvent 2.2 [4] | 1.410E+08 | 79% | Gruber et al (2011) [7] | 42.5 | 31.6 | 58.7 | 6.0 | 3.1 | 2.4 | 4.1 | 4.33E+08 |
|  | **Li TOTAL** |  |  |  |  |  |  |  | **1.410E+08** | **100%** |  | **125** | **94.0** | **161** | **6.0** | **7.1** | **5.7** | **8.9** | **4.33E+08** |
| 4 | Be | Bertrandite | None | NA | Metal |  | Be metal | Own assessment | 4.950E+04 | 12% | BeST (2012) [11] | 12000 | 7170 | 24800 | 0.6 | 879 | 583 | 1450 | 4.35E+07 |
|  | Be(OH)_2_ | Bertrandite | None | NA | Metal |  | Be(OH)2 | Own assessment | 3.505E+05 | 88% | BeST (2012) [11] | 319 | 224 | 495 | 0.1 | 19 | 14.8 | 25.5 | 6.66E+06 |
|  | **Be TOTAL** |  |  |  |  |  |  |  | **4.000E+05** | **100%** |  | **1720** | **1020** | **3440** | **0.7** | **122** | **79.9** | **191** | **5.02E+07** |
| 5 | Borax | Sodium borates | None | NA | Oxide |  | Borax, anhydrous, powder, at plant (GLO) | Ecoinvent 2.2 [4] | 4.274E+09 | 81% | USGS (2008) [15] | 30.7 | 17.4 | 51.8 | 131 | 1.6 | 1 | 2.6 | 7.04E+09 |
|  | Boric acid | Colemanite (calcium borate) | None | NA | Acid |  | Boric acid, anhydrous, powder, at plant (RER) | Ecoinvent 2.2 [4] | 1.003E+09 | 19% | USGS (2008) [15] | 13.0 | 8.6 | 20.5 | 13.0 | 0.7 | 0.5 | 1.2 | 7.13E+08 |
|  | **B TOTAL** |  |  |  |  |  |  |  | **5.277E+09** | **100%** |  | **27.3** | **15.5** | **46.4** | **144** | **1.5** | **0.9** | **2.4** | **7.75E+09** |
| 12 | Mg | Seawater | None | NA | Metal |  | Magnesium, at plant/RER U | Ecoinvent 2.2 [4] | - | 6% | Roskill (2005) [88] | 265 | 194 | 363 | - | 73.7 | 20.4 | 225 | - |
|  | MgO | Seawater | None | NA | Oxide |  | Magnesium oxide, at plant/RER U | Ecoinvent 2.2 [4] | 2.146E+10 | 89% | Roskill (2005) [88] | 2.8 | 1.4 | 5.1 | 61.1 | 1.1 | 0.5 | 1.9 | 2.26E+10 |
|  | MgSO_4_ | Seawater | None | NA | Sulfate |  | Magnesium sulphate, at plant/RER U | Ecoinvent 2.2 [4] | - | 5% | Roskill (2005) [88] | 6.3 | 2.7 | 12.6 | - | 0.3 | 0.1 | 0.6 | - |
|  | **Mg TOTAL** |  |  |  |  |  |  |  | **2.146E+10** | **100%** |  | **18.8** | **14.1** | **24.4** | **61.1** | **5.4** | **2** | **14.4** | **2.26E+10** |
| 13 | Al | Bauxite | Ga | NA | Metal |  | Aluminum, primary, at plant/RER U | Ecoinvent 2.2 [4] | 3.038E+10 | 64% | Rombach (2013) [89] | 194 | 158 | 246 | 5897 | 12.2 | 10.3 | 14.5 | 3.72E+11 |
|  | Al (new scrap) | Primary scrap | None | NA | Metal |  | Aluminum, secondary, from new scrap, at plant/RER U | Ecoinvent 2.2 [4] | 9.204E+09 | 20% | Rombach (2013) [89] | 8.3 | 6.6 | 10.7 | 76.6 | 0.4 | 0.4 | 0.5 | 3.86E+09 |
|  | Al (old scrap) | Secondary scrap | None | NA | Metal |  | Aluminum, secondary, from old scrap, at plant/RER U | Ecoinvent 2.2 [4] | - | 16% | Rombach (2013) [89] | 23.8 | 18.6 | 31.1 | - | 1.4 | 1.2 | 1.6 | - |
|  | **Al TOTAL** |  |  |  |  |  |  |  | **3.959E+10** | **100%** |  | **131** | **97.4** | **172** | **5974** | **8.2** | **6.3** | **10.7** | **3.75E+11** |
| 20 | CaCO3 | Dolomite | None | NA | Carbonate |  | Quicklime, milled, loose, at plant/CH U | Ecoinvent 2.2 [4] | 3.070E+11 | 99.99% | USGS (2010) [26] | 5.8 | 4.6 | 7.1 | 1787 | 1.0 | 0.8 | 1.2 | 3.00E+11 |
|  | Ca | Dolomite | None | NA | Metal |  | Calcium metal, at plant | Own assessment | 3.200E+07 | 0.01% | InfoMine (2012) [27] | 223 | 124 | 518 | 7.1 | 16.3 | 10.2 | 31.1 | 5.21E+08 |
|  | **Ca TOTAL** |  |  |  |  |  |  |  | **3.070E+11** | **100%** |  | **5.8** | **4.7** | **7.2** | **1794** | **1.0** | **0.7** | **1.2** | **3.01E+11** |
| 21 | Sc_2_O_3_ | Various ores | Various | NA | Oxide |  | Scandium, at plant | Own assessment | 1.000E+04 | 100% | USGS (2013) [32] | 97200 | 53800 | 259000 | 1.0 | 5710 | 5290 | 6570 | 5.71E+07 |
|  | **Sc TOTAL** |  |  |  |  |  |  |  | **1.000E+04** | **100%** |  | **97200** | **53800** | **259000** | **1.0** | **5710** | **5290** | **6570** | **5.71E+07** |
| 22 | TiO_2_ (from Ilmenite) | Ilmenite | Ti, Zr, Hf, REOs, Th | $ 0.37 | Ilmenite, 54% TiO2, f.o.b. Australia | USGS (2011) [84] | Titanium dioxide, sulfate process, at plant/GLO U, reallocated | Ecoinvent 3 [90] | 3.373E+09 | 45% | Ceresana (2013) [35] | 80.7 | 67.6 | 95.2 | 272 | 7.2 | 6.6 | 7.8 | 2.42E+10 |
|  | TiO_2_ (from Rutile) | Rutile | Ti, Zr, Hf, REOs, Th | $ 0.52 | Rutile, 95% TiO2, f.o.b. Australia | USGS (2011) [84] | Titanium dioxide, chloride process, at plant/GLO U, reallocated | Ecoinvent 3 [90] | 3.373E+09 | 50% | Ceresana (2013) [35] | 88.0 | 74.8 | 103 | 297 | 5.2 | 4.6 | 5.9 | 1.75E+10 |
|  | Ti | Ilmenite/Rutile | Ti, Zr, Hf, REOs, Th | NA | Metal |  | Titanium I | Idemat 2001 [34] | - | 5% | USGS (2008) [15]; the 5% actually include fluxes, metal, and welding rod coatings | 703 | 503 | 956 | - | 45.1 | 32 | 61.3 | - |
|  | **Ti TOTAL** |  |  |  |  |  |  |  | **6.745E+09** | **100%** |  | **115** | **103** | **130** | **569** | **8.1** | **7.6** | **8.7** | **4.17E+10** |
| 23 | V | Vanadium-bearing Ores (Magnetite, Uranium-Ore, Phosphate, Bauxite) | Various | NA | Metal |  | Vanadium I | Idemat 2001 [34] | 5.636E+07 | 81.0% (assumed to be 100% as no LCI data is available on other V forms) | Liddell et al, 2011 [37] | 516 | 306 | 844 | 29.1 | 33.1 | 19.4 | 53 | 1.87E+09 |
|  | **V TOTAL** |  |  |  |  |  |  | **No data on V from steel slags and oil residues available.** | **5.636E+07** | **100%** |  | **516** | **306** | **844** | **29.1** | **33.1** | **19.4** | **53** | **1.87E+09** |
| 24 | FeCr | Chromite ore | None | NA | Alloy |  | Ferrochromium, high-carbon, 68% Cr, at plant/GLO U | Ecoinvent 2.2 [4] | 8.060E+09 | 75% | Classen et al (2009) [13]. For subsequent stainless steel production. Percentages for 2003. | 37.3 | 31.6 | 45.0 | 300 | 1.9 | 1.6 | 2.3 | 1.55E+10 |
|  | Cr | Chromite ore | None | NA | Metal |  | Chromium, at regional storage/RER U | Ecoinvent 2.2 [4] | - | 1% | Classen et al (2009) [13]. For subsequent stainless steel production. Percentages for 2003. | 577 | 460 | 721 | - | 26.7 | 21.8 | 32.1 | - |
|  | Na_2_Cr_2_O_7_ | Chromite ore | None | NA | Chemical |  | Sodium dichromate, at plant/RER U | Ecoinvent 2.2 [4] | - | 17% | Classen et al (2009) [13]. For subsequent stainless steel production. Percentages for 2003. | 54.6 | 43.2 | 72.0 | - | 4.8 | 4.4 | 5.3 | - |
|  | Chromite | Chromite ore | None | NA | Mineral |  | Chromite, ore concentrate, at beneficiation, GLO U | Ecoinvent 2.2 [4] | 1.052E+10 | 8% | Classen et al (2009) [13]. For subsequent stainless steel production. Percentages for 2003. | 0.5 | 0.4 | 0.7 | 5.6 | 0 | 0 | 0 | 2.75E+08 |
|  | **Cr TOTAL** |  |  |  |  |  |  |  | **1.858E+10** | **100%** |  | **40.2** | **34.2** | **47.2** | **306** | **2.4** | **2.2** | **2.7** | **1.57E+10** |
| 25 | FeMn | Manganese Ore | None | NA | Alloy |  | Ferromanganese, high-coal, 74.5% Mn, at regional storage | Ecoinvent 2.2 [4] | 5.864E+09 | 84% | Anger et al (2000); Classen et al (2009) [13,91] | 23.5 | 18.9 | 29.4 | 138 | 1.0 | 0.8 | 1.2 | 5.72E+09 |
|  | SiMn | Manganese Ore | None | NA | Alloy |  | Ferromanganese, high-coal, 74.5% Mn, at regional storage | Ferromanganese used as proxy | 8.631E+09 | 13% | Anger et al (2000); Classen et al (2009) [13,91] | 23.5 | 18.9 | 29.4 | 203 | 1.0 | 0.8 | 1.2 | 8.41E+09 |
|  | Mn | Manganese Ore | None | NA | Metal |  | Manganese, at regional storage/RER U | Ecoinvent 2.2 [4] | - | 1% | Anger et al (2000); Classen et al (2009) [13,91] | 58.6 | 42.6 | 83.7 | - | 2.6 | 1.8 | 3.6 | - |
|  | Mn conc | Manganese Ore | None | NA | Mineral |  | Manganese concentrate, at beneficiation GLO/U | Ecoinvent 2.2 [4] | - | 1% | Anger et al (2000); Classen et al (2009) [13,91] | 0.3 | 0.2 | 0.4 | - | 0.01 | 0.01 | 0.02 | - |
|  | Mn_2_O_3_ | Manganese Ore | None | NA | Oxide |  | Manganese oxide (MN2O3), at plant/CNU U | Ecoinvent 2.2 [4] | - | 1% | Anger et al (2000); Classen et al (2009) [13,91] | 37.9 | 26.4 | 51.4 | - | 2.4 | 1.9 | 3.1 | - |
|  | **Mn TOTAL** |  |  |  |  |  |  |  | **1.449E+10** | **100%** |  | **23.7** | **19.1** | **30.4** | **340** | **1.0** | **0.8** | **1.3** | **1.41E+10** |
| 26 | Fe | Iron ore (Fe 46.7%) | None | NA | Metal |  | Pig iron, at plant/GLO U | Ecoinvent 2.2 [4] | 9.973E+11 | 100% |  | 23.1 | 20.9 | 25.5 | 23033 | 1.5 | 1.4 | 1.7 | 1.51E+12 |
|  | **Fe TOTAL** |  |  |  |  |  |  |  | **9.973E+11** | **100%** | Only includes pig iron (i.e., not representative of the iron and steel sector) | **23.1** | **20.9** | **25.5** | **23033** | **1.5** | **1.4** | **1.7** | **1.51E+12** |
| 27 | Co | Various ores | None | NA | Metal |  | Cobalt, at plant, GLO U | Ecoinvent 2.2 [4] | 5.729E+07 | 100% |  | 128 | 93.2 | 179 | 7.4 | 8.3 | 6 | 11.5 | 4.75E+08 |
|  | **Co TOTAL** |  |  |  |  |  |  |  | **5.729E+07** | **100%** |  | **128** | **93.2** | **179** | **7.4** | **8.3** | **6** | **11.5** | **4.75E+08** |
| 28 | FeNi | Nickel, 1.98% in silicates, 1.04% in crude ore | None | NA | Alloy |  | Ferronickel, 25% Ni, at plant/GLO U | Ecoinvent 2.2 [4] | 1.392E+09 | 21% | USGS (2009) [92] | 156 | 135 | 183 | 217 | 9.2 | 8.4 | 10.2 | 1.28E+10 |
|  | Ni (from Cu) | Nickel sulfides | Cu | $ 22.40 | Metal | USGS (2011) [84] | Nickel, 99.5%, at plant/GLO U, reallocated | Ecoinvent 2.2 [4] | 4.652E+08 | 28% | USGS (2009) [92] | 187 | 166 | 216 | 87.1 | 10.9 | 9.7 | 12.3 | 5.05E+09 |
|  | Ni ( from PGM, ZA) | Bushveld Mine, PGM-dominated | PGMs, Co | $ 22.40 | Metal | USGS (2011) [84] | Nickel, primary, from platinum group metal production, ZA U, reallocated | Ecoinvent 2.2 [4] | 2.993E+07 | 2% | USGS (2009) [92] | 155 | 110 | 211 | 4.6 | 7.8 | 5.9 | 10.5 | 2.34E+08 |
|  | Ni (from PGM, RU) | Noril'sk Mine, Ni-Cu-dominated | PGMs, Co | $ 22.40 | Metal | USGS (2011) [84] | Nickel, primary, from platinum group metal production, RU U, reallocated | Ecoinvent 2.2 [4] | 2.402E+08 | 14% | USGS (2009) [92] | 150 | 112 | 196 | 36.1 | 8.3 | 6.5 | 10.9 | 1.99E+09 |
|  | Ni (secondary) | Scrap | Cu, Various other precious metals | $ 22.40 | Metal | USGS (2011) [84] | Nickel, secondary, from electronic and electric scrap recycling , at refinery/SE U | Ecoinvent 2.2 [4] | - | 35% | Informed estimate | 6.2 | 2.8 | 13.4 | - | 0.7 | 0.3 | 1.8 | - |
|  | **Ni TOTAL** |  |  |  |  |  |  |  | **7.353E+08** | **100%** |  | **111** | **96.4** | **129** | **345** | **6.5** | **5.9** | **7.3** | **2.01E+10** |
| 29 | Cu (from Mo) | Copper ore | Mo | $ 6.45 | Concentrate | 2006-2010 copper price (USGS (2013) [32]) minus treatment and refining charges (TC/RCs) (Reuters (2013) [93]). | Copper, primary, at refinery/GLO U, realloc (with Mo) | Ecoinvent 2.2 [4] | 1.665E+10 | 77% | Nassar et al (2012); RMG (2006) [38,43] | 59.3 | 49.7 | 70.8 | 987 | 3.1 | 2.6 | 3.7 | 5.12E+10 |
|  | Cu (from PGM, ZA) | Bushveld Mine, PGM-dominated | PGMs, Ni | $ 6.70 | Metal | USGS (2011) [84] | Copper, primary, from platinum metal production, ZA U, reallocated | Ecoinvent 2.2 [4] | - | 0% | Nassar et al (2012); RMG (2006) [38,43] | 46.2 | 33.0 | 63.7 | - | 2.3 | 1.7 | 3.1 | - |
|  | Cu (from PGM, RU) | Noril'sk Mine, Ni-Cu-dominated | PGMs, Ni | $ 6.70 | Metal | USGS (2011) [84] | Copper, primary, from platinum metal production, RU U, reallocated | Ecoinvent 2.2 [4] | 1.390E+08 | 0% | Nassar et al (2012); RMG (2006) [38,43] | 44.9 | 32.9 | 61.2 | 6.2 | 2.5 | 1.9 | 3.2 | 3.44E+08 |
|  | Cu (combined metal, SE) | Au-Ag ores | Zn, Ag, Zn, Pb | $ 6.70 | Metal | USGS (2011) [84] | Copper, from combined metal production, at refinery/SE U | Ecoinvent 2.2 [4] | 5.145E+08 | 3% | Nassar et al (2012); RMG (2006) [38,43] | 87.8 | 67.1 | 114 | 45.2 | 2.3 | 2 | 2.7 | 1.17E+09 |
|  | Cu (from Ni) | Nickel sulfides | Ni | $ 6.70 | Metal | USGS (2011) [84] | Copper, primary, couple production nickel, reallocated | Ecoinvent 2.2 [4] | 3.571E+08 | 4% | Nassar et al (2012); RMG (2006) [38,43] | 64.8 | 55.6 | 77.0 | 23.2 | 5.0 | 4.4 | 5.7 | 1.80E+09 |
|  | Cu (secondary, electr. scrap, SE) | Electronic scrap | Au, Ag, Ni, Pb, Pd | $ 6.70 | Metal | USGS (2011) [84] | Copper, secondary, from electronic and electric scrap recycling, at refinery/SE U, reallocated | Ecoinvent 2.2 [4] | - | 8% | Nassar et al (2012) [43]; Assuming equal shares of secondary production | 1.8 | 0.8 | 4.1 | - | 0.2 | 0.1 | 0.5 | - |
|  | Cu (secondary) | Scrap | None | NA | Metal |  | Copper, secondary, at refinery/RER U | Ecoinvent 2.2 [4] | - | 8% | Nassar et al (2012) [43]; Assuming equal shares of secondary production | 28.1 | 23.6 | 33.7 | - | 1.8 | 1.6 | 2.1 | - |
|  | **Cu TOTAL** |  |  |  |  |  |  |  | **1.766E+10** | **100%** |  | **53.7** | **42.1** | **68.6** | **1062** | **2.8** | **2.2** | **3.5** | **5.45E+10** |
| 30 | Zn | Lead/Zinc ores | Pb | $ 2.57 | Metal | USGS (2011) [84] | Zinc, primary, at regional storage/RER U reallocated | Ecoinvent 2.2 [4] | 1.089E+10 | 88% | Informed estimate | 54.4 | 37.2 | 82.1 | 592 | 3.2 | 2.5 | 4.4 | 3.52E+10 |
|  | Zn (from Au-Ag, SE) | Au-Ag ores | Au, Ag, Cu, Pb | $ 2.57 | Metal | USGS (2011) [84] | Zinc, from combined metal production, at refinery/SE U, realloc | Ecoinvent 2.2 [4] | 8.112E+08 | 7% | Informed estimate | 32.9 | 25.9 | 42.2 | 26.7 | 0.9 | 0.7 | 1 | 6.94E+08 |
|  | Zn | Scrap | None | NA | Metal |  | Zinc, secondary, GLO | Not modeled due to a lack of data |  | 6% | Informed estimate | - | - | - | - | - | - | - | - |
|  | **Zn TOTAL** |  |  |  |  |  |  |  | **1.170E+10** | **101%** |  | **52.9** | **37.1** | **80.3** | **619** | **3.1** | **2.4** | **4.1** | **3.59E+10** |
| 31 | Ga | Bauxite | Al(OH)3 | NA | Metal | USGS (2011) [84] | Gallium, semiconductor-grade, at plant/GLO U | Ecoinvent 2.2 [4] | 8.600E+04 | 100% |  | 3030 | 1520 | 5350 | 0.3 | 205 | 128 | 331 | 1.76E+07 |
|  | **Ga TOTAL** |  |  |  |  |  |  |  | **8.600E+04** | **100%** |  | **3030** | **1520** | **5350** | **0.3** | **205** | **128** | **331** | **1.76E+07** |
| 32 | Ge | Lead/Zinc ores | Zn, Cd, In | $ 1,172.00 | Metal | USGS (2011) [84] | Germanium, at regional storage/RER U reallocated | Ecoinvent 2.2 [4] | 5.900E+04 | 100% |  | 2890 | 1870 | 4570 | 0.2 | 170 | 121 | 252 | 1.01E+07 |
|  | **Ge TOTAL** |  |  |  |  |  |  |  | **5.900E+04** | **100%** |  | **2890** | **1870** | **4570** | **0.2** | **170** | **121** | **252** | **1.01E+07** |
| 33 | As_2_O_3_ | Copper ores | Cu | $ 0.46 | Oxide |  | Arsenic trioxide, allocated from blister copper -production/GLO U | Ecoinvent 2.2 [4] | 5.336E+07 | 86% | Nassar et al (2012) [43] | 3.1 | 2.6 | 3.8 | 0.2 | 0.2 | 0.1 | 0.2 | 8.78E+06 |
|  | As | Copper ores | Cu | $ 2.21 | Metal |  | Arsenic (from ProBas) | UBA (2010) [42] | - | 14% | Nassar et al (2012) [43] | 16.5 | 14.0 | 19.5 | - | 1.3 | 1.1 | 1.6 | - |
|  | **As TOTAL** |  |  |  |  |  |  |  | **5.336E+07** | **100%** |  | **5.0** | **4.4** | **5.7** | **0.2** | **0.3** | **0.3** | **0.4** | **8.78E+06** |
| 34 | Se | Cu anode slime | Cu, Te, Ag | $ 66.51 | Metal | USGS (2011) [84] | Selenium, at plant/RER U upstream included | Ecoinvent 2.2 [4] | 2.374E+06 | 100% |  | 65.5 | 49.2 | 88.4 | 0.2 | 3.6 | 2.7 | 4.8 | 8.548,757 |
|  | **Se TOTAL** |  |  |  |  |  |  |  | **2.374E+06** | **100%** |  | **65.5** | **49.2** | **88.4** | **0.2** | **3.6** | **2.7** | **4.8** | **8.548,757** |
| 38 | SrCO_3_ | Celestite (SrSO4) | None | NA | Carbonate |  | Strontium carbonate, at plant | Own assessment | - | 100% |  | 48.8 | 27.7 | 89.7 | - | 3.2 | 2 | 4.8 | - |
|  | SrSO_4_ (90% Sr) | Celestite (SrSO4) | None | NA | Sulfate |  | Celestite (90% SrSO4), at plant | Own assessment | 6.556E+08 | 0% |  | 14.5 | 6.2 | 33.9 | 9.5 | 0.9 | 0.4 | 1.6 | 5.73E+08 |
|  | **Sr TOTAL** |  |  |  |  |  |  |  | **-** | **100%** |  | **48.8** | **27.7** | **89.7** | **9.5** | **3.2** | **2.1** | **4.7** | **5.73E+08** |
| 39 | Y_2_O_3_ | Bastnaesite, China | REOs | $ 48.80 | Oxide | USGS (2011) [84] | Yttrium concentrate, at plant, CN | Own assessment | 1.394E+07 | 100% |  | 295 | 231 | 374 | 4.1 | 15.1 | 11.9 | 19.2 | 2.10E+08 |
|  | **Y TOTAL** |  |  |  |  |  |  |  | **1.394E+07** | **100%** |  | **295** | **231** | **374** | **4.1** | **15.1** | **11.9** | **19.2** | **2.10E+08** |
| 40 | ZrO_2_ | Zircon | Hf | Mass allocation | Oxide |  | Zirconium oxide, at plant/AU U | Ecoinvent 2.2 [4] | 1.310E+09 | 94% | Informed estimate | 62.2 | 47.4 | 82.4 | 81.5 | 3.4 | 2.7 | 4.2 | 4.39E+09 |
|  | Zr | Zircon | Hf | $ 40.40 | Sponge | USGS (2011) [84] | Zirconium, metal ingot | Own assessment | - | 5% | Informed estimate | 576 | 298 | 1140 | - | 21.4 | 11.7 | 37.8 | - |
|  | ZrSiO_4_ (50% Zr) | Zircon | Ilmenite, Rutile | $ 0.80 | ZrSiO4 | USGS (2011) [84] | Zircon, 50% zirconium, at plant/AU U | Ecoinvent 2.2 [4] | - | 1% | Informed estimate | 11.8 | 9.5 | 14.7 | - | 0.7 | 0.6 | 0.9 | - |
|  | **Zr TOTAL** |  |  |  |  |  |  |  | **-** | **100%** |  | **19.9** | **15.2** | **27.0** | **81.5** | **1.1** | **0.9** | **1.3** | **4.39E+09** |
| 41 | Nb | Pyrochlore | Ta, Sn slags | NA | NA |  | Niobium, pure (>99.75%) | Own assessment | - | 11% | BGS (2012) [94] | 639 | 453 | 902 | - | 46.6 | 34.4 | 64 | - |
|  | FeNb | Pyrochlore | Ta, Sn slags | NA | NA |  | Ferroniobium (FeNb), at plant, GLO | Own assessment | 4.000E+07 | 89% | BGS (2012) [94] | 114 | 82.3 | 153 | 4.6 | 8.3 | 6.2 | 11.1 | 3.33E+08 |
|  | **Nb TOTAL** |  |  |  |  |  |  |  | **4.000E+07** | **100%** |  | **172** | **132** | **224** | **4.6** | **12.5** | **10.1** | **15.4** | **3.33E+08** |
| 42 | Mo | Copper ores, Molybdenite ores | Cu | $ 49.03 | Metal | USGS (2013) [32] | Molybdenum, at regional storage/RER U, realloc | Ecoinvent 2.2 [4] | - | 6% | SMR (2009) [95] | 235 | 115 | 455 | - | 11.7 | 4.8 | 20 | - |
|  | MoS_2_ | Molybdenite ores | None | NA | Concentrate |  | Molybdenum concentrate, main product/GLO U | Ecoinvent 2.2 [4] | 1.528E+08 | 40% | Classen et al (2009) [13] | 53.2 | 42.8 | 67.7 | 8.1 | 2.6 | 2 | 3.3 | 3.95E+08 |
|  | MoS_2_ (from Cu) | Copper ores | Cu | $ 49.03 | Sulfide | USGS (2013) [32] | Molybdenum concentrate, couple production Cu/GLO U, realloc | Ecoinvent 2.2 [4] | 2.110E+08 | 55% | Classen et al (2009) [13] | 150 | 122 | 187 | 31.7 | 7.3 | 5.7 | 9.3 | 1.54E+09 |
|  | **Mo TOTAL** |  |  |  |  |  |  |  | **3.639E+08** | **100%** |  | **117** | **95.3** | **145** | **39.9** | **5.7** | **4.5** | **7.3** | **1.94E+09** |
| 44 | Ru (ZA) | Bushveld Mine, PGM-dominated | Cu, Ni, PGMs | $ 7,899.50 | Metal | USGS (2011) [84] | _Rhuthenium, primary, at refinery/ZA U, reallocated | Own assessment | 2.747E+04 | 86% | USGS (2010) [26] | 40700 | 29200 | 54800 | 1.1 | 2060 | 1510 | 2700 | 5.66E+07 |
|  | Ru (RU) | Noril'sk Mine, Ni-Cu-dominated | Cu, Ni, PGMs | $ 7,899.50 | Metal | USGS (2011) [84] | _Rhuthennium, primary, at refinery/RU U, reallocated | Own assessment | 4.536E+03 | 14% | USGS (2010) [26] | 43210 | 32100 | 57100 | 0.2 | 2390 | 1920 | 3080 | 1.09E+07 |
|  | **Ru TOTAL** |  |  |  |  |  |  |  | **3.201E+04** | **100%** |  | **41100** | **29700** | **54700** | **1.3** | **2110** | **1660** | **2690** | **6.74E+07** |
| 45 | Rh (ZA) | Bushveld Mine, PGM-dominated | Cu, Ni, PGMs | $ 122,800.98 | Metal | USGS (2011) [84] | _Rhodium, primary, at refinery/ZA U, reallocated | Ecoinvent 2.2 [4] | 2.028E+04 | 65% | USGS (2010) [26] | 866200 | 633000 | 1150000 | 17.6 | 43800 | 32700 | 59000 | 8.88E+08 |
|  | Rh (RU) | Noril'sk Mine, Ni-Cu-dominated | Cu, Ni, PGMs | $ 122,800.98 | Metal | USGS (2011) [84] | _Rhodium, primary, at refinery/RU U, reallocated | Ecoinvent 2.2 [4] | 3.664E+03 | 12% | USGS (2010) [26] | 919000 | 692000 | 1240000 | 3.4 | 50800 | 40200 | 64900 | 1.86E+08 |
|  | Rh (secondary) | Catalysts, etc. | Pt, Pd | $ 122,800.98 | Metal | USGS (2011) [84] | Rhodium, secondary, at refinery/RER U (Secondary), reallocated | Ecoinvent 2.2 [4] | - | 23% | USGS (2010) [26] | 33600 | 23300 | 46500 | - | 1780 | 1320 | 2400 | - |
|  | **Rh TOTAL** |  |  |  |  |  |  |  | **2.394E+04** | **100%** |  | **683000** | **504000** | **911000** | **20.9** | **35100** | **26700** | **45500** | **1.07E+09** |
| 46 | Pd (ZA) | Bushveld Mine, PGM-dominated | Cu, Ni, PGMs | $ 12,324.69 | Metal | USGS (2011) [84] | _Palladium, primary, at refinery/ZA U, reallocated | Ecoinvent 2.2 [4] | 6.147E+04 | 38% | USGS (2010) [26] | 87000 | 62600 | 116000 | 5.3 | 4400 | 3190 | 5930 | 2.70E+08 |
|  | Pd (RU) | Noril'sk Mine, Ni-Cu-dominated | Cu, Ni, PGMs | $ 12,324.69 | Metal | USGS (2011) [84] | _Palladium, primary, at refinery/RU U, reallocated | Ecoinvent 2.2 [4] | 1.077E+05 | 43% | USGS (2010) [26] | 92200 | 68200 | 118000 | 9.9 | 5110 | 4090 | 6450 | 5.50E+08 |
|  | Pd (secondary) | Catalysts, etc. | Pt, Rh | $ 12,324.69 | Metal | USGS (2011) [84] | _Palladium, secondary, at refinery/RER U, reallocated | Ecoinvent 2.2 [4] | - | 15% | USGS (2010) [26] | 3370 | 2360 | 4780 | - | 179 | 134 | 242 | - |
|  | Pd (secondary, E-scrap) | Electronic scrap | Au, Ag, Ni, Pb, Cu | $ 12,324.69 | Metal | USGS (2011) [84] | Palladium, secondary, at precious metal refinery/SE U | Ecoinvent 2.2 [4] | - | 5% | Informed estimate | 3560 | 1640 | 7410 |  | 404 | 143 | 895 | - |
|  | **Pd TOTAL** |  |  |  |  |  |  |  | **1.692E+05** | **100%** |  | **72700** | **55500** | **95500** | **15.3** | **3880** | **3090** | **4860** | **8.21E+08** |
| 47 | Ag (from Cu) | Copper ores | Cu | $ 481.36 | Silver | USGS (2011) [84] | Silver, from copper production, at refinery/GLO U re-allocated | Ecoinvent 2.2 [4] | 4.484E+06 | 17% | Thomson Reuters (2012) [96] | 4620 | 3610 | 5790 | 20.7 | 240 | 195 | 296 | 1.07E+09 |
|  | Ag (from Pb) | Lead ores | Pb | $ 481.36 | Metal | USGS (2011) [84] | Silver, from lead production, at refinery/GLO U reallocated | Ecoinvent 2.2 [4] | 7.687E+06 | 28% | Thomson Reuters (2012) [96] | 690 | 550 | 869 | 5.3 | 55.4 | 46 | 66.7 | 4.26E+08 |
|  | Ag (from Au-Ag) | Gold ores | Au | $ 481.36 | Metal | USGS (2011) [84] | Silver, from combined gold-silver production, at refinery/GLO U, realloc | Ecoinvent 2.2 [4] | 9.181E+06 | 34% | Thomson Reuters (2012) [96] | 6740 | 5430 | 8660 | 61.9 | 405 | 313 | 516 | 3.71E+09 |
|  | Ag (secondary, E-scrap) | Electronic scrap | Au, Ni, Pb, Pd, Cu | $ 481.36 | Metal | USGS (2011) [84] | Silver, secondary, at precious metal refinery/SE U, reallocated | Ecoinvent 2.2 [4] | - | 21% | Nassar et al (2012) [43] | 139 | 63.8 | 295 | - | 15.7 | 5.9 | 36 | - |
|  | **Ag TOTAL** |  |  |  |  |  |  |  | **2.135E+07** | **100%** |  | **3280** | **2740** | **3950** | **87.9** | **196** | **164** | **234** | **5.21E+06** |
| 48 | Cd | Lead/Zinc ores | Zn, In, Ge | $ 4.66 | Metal | USGS (2011) [84] | Cadmium, primary, at plant/GLO U reallocated | Ecoinvent 2.2 [4] | 1.139E+07 | 50% | Own assumption | 27.5 | 20.6 | 36.1 | 0.3 | 1.4 | 1.1 | 1.9 | 1.65E+07 |
|  | Cd (semicond. grade) | Lead/Zinc ores | Zn, In, Ge | $ 4.66 | Metal | USGS (2011) [84] | Cadmium, semiconductor-grade, at plant/US U reallocated | Ecoinvent 2.2 [4] | 1.139E+07 | 50% | Own assumption | 78.4 | 30.5 | 198 | 0.9 | 4.5 | 2.2 | 8.9 | 5.13E+07 |
|  | **Cd TOTAL** |  |  |  |  |  |  |  | **2.279E+07** | **100%** |  | **53.0** | **28.1** | **118** | **1.2** | **3.0** | **1.8** | **5.1** | **1.65E+07** |
| 49 | In | Lead/Zinc ores | Zn Cd, Ge | $ 692.60 | Metal | USGS (2011) [84] | Indium, at regional storage/RER U reallocated | Ecoinvent 2.2 [4] | 6.080E+05 | 100% |  | 1720 | 1170 | 2520 | 1.0 | 102 | 78.8 | 155 | 6.17E+07 |
|  | **In TOTAL** |  |  |  |  |  |  |  | **6.080E+05** | **100%** |  | **1720** | **1170** | **2520** | **1.0** | **102** | **78.8** | **155** | **6.17E+07** |
| 50 | Sn | Cassiterite (SnO2) | None | NA | Metal |  | Tin, at regional storage/RER U | Ecoinvent 2.2 [4] | 3.334E+08 | 95% | USGS (2008) [15] | 321 | 201 | 496 | 107 | 17.1 | 11 | 26.8 | 5.70E+09 |
|  | Sn | New scrap, Tinplate | None | NA | Metal |  | Tin, secondary | Own assessment | - | 6% | USGS (2008) [15] | - | - | - | - | - | - | - | - |
|  | **Sn TOTAL** |  |  |  |  |  |  |  | **3.334E+08** | **100%** |  | **321** | **201** | **496** | **107** | **17.1** | **11** | **26.8** | **5.70E+09** |
| 51 | Sb | Stibnite | None | NA | Metal |  | Antimony, at refinery/CN U | Ecoinvent 2.2 [4] | 1.822E+08 | 91% | USGS (2008); Classen et al (2009) [13,15] | 141 | 68.4 | 342 | 25.8 | 12.9 | 7.2 | 23.9 | 2.35E+09 |
|  | Sb | Lead ores | Pb | NA | Metal |  | Antimony, by-product from lead mining | Not included due to a lack of data | - | 9% | USGS (2008); Classen et al (2009) [13,15] | - | - | - | - | - | - | - | - |
|  | Sb | Secondary Sources | No | NA | Metal |  | Antimony, from secondary sources | Not included due to a lack of data | - | ? |  | - | - | - | - | - | - | - | - |
|  | **Sb TOTAL** |  |  |  |  |  |  |  | **1.822E+08** | **100%** |  | **141** | **68.4** | **342** | **25.8** | **12.9** | **7.2** | **23.9** | **2.35E+09** |
| 52 | Te | Cu anode slime | Cu, Se, Ag | $ 150.40 | Metal | USGS (2011) [84] | Tellurium, semiconductor-grade, at plant/GLO U reallocated | Ecoinvent 2.2 [4] | 1.405E+05 | 100% | Own assumption | 435 | 332 | 559 | 0.1 | 21.9 | 17.1 | 27.3 | 3.08E+06 |
|  | **Te TOTAL** |  |  |  |  |  |  |  | **1.405E+05** | **100%** |  | **435** | **332** | **559** | **0.1** | **21.9** | **17.1** | **27.3** | **3.08E+06** |
| 56 | BaSO_4_ | Barite | None | NA | Sulfate |  | Barite, at plant/RER U | Ecoinvent 2.2 [4] | 9.177E+09 | 100% | Kresse et al (2012) [63] | 4.0 | 2.6 | 5.9 | 36.5 | 0.2 | 0.1 | 0.3 | 1.73E+09 |
|  | **Ba TOTAL** |  |  |  |  |  |  |  | **9.177E+09** | **100%** |  | **4.0** | **2.6** | **5.9** | **36.5** | **0.2** | **0.1** | **0.3** | **1.73E+09** |
| 57 | La_2_O_3_ | Bastnaesite, China | REOs | $ 35.60 | Oxide | USGS (2011) [84] | Lanthanum oxide, at plant, CN | Ecoinvent 2.2 [4] | 2.581E+07 | 100% |  | 216 | 172 | 275 | 5.6 | 11.0 | 8.8 | 14.3 | 2.84E+08 |
|  | **La TOTAL** |  |  |  |  |  |  |  | **2.581E+07** | **100%** |  | **215** | **172** | **275** | **5.6** | **11.0** | **8.8** | **14.3** | **2.84E+08** |
| 58 | CeO_2_ | Bastnaesite, China | REOs | $ 41.67 | Oxide | USGS (2011) [84] | Cerium concentrate, 60% cerium oxide, at plant, CN | Ecoinvent 2.2 [4] | 3.418E+07 | 100% |  | 252 | 198 | 326 | 8.6 | 12.9 | 10.1 | 16.4 | 4.40E+08 |
|  | **Ce TOTAL** |  |  |  |  |  |  |  | **3.418E+07** | **100%** |  | **252** | **198** | **326** | **8.6** | **12.9** | **10.1** | **16.4** | **4.40E+08** |
| 59 | Pr_6_O_11_ | Bastnaesite, China | REOs | $ 62.08 | Oxide | USGS (2011) [84] | Praesodymium oxide, at plant, CN | Ecoinvent 2.2 [4]Ecoinvent 2.2 | 4.979E+06 | 100% |  | 376 | 301 | 475 | 1.9 | 19.2 | 14.9 | 24.7 | 9.55E+07 |
|  | **Pr TOTAL** |  |  |  |  |  |  |  | **4.979E+06** | **100%** |  | **376** | **301** | **475** | **1.9** | **19.2** | **14.9** | **24.7** | **9.55E+07** |
| 60 | Nd_2_O_3_ | Bastnaesite, China | REOs | $ 56.84 | Oxide | USGS (2011) [84] | Neodymium oxide, at plant, CN | Ecoinvent 2.2 [4] | 1.788E+07 | 100% |  | 344 | 267 | 437 | 6.1 | 17.6 | 13.8 | 22.9 | 3.14E+08 |
|  | **Nd TOTAL** |  |  |  |  |  |  |  | **1.788E+07** | **100%** |  | **344** | **267** | **437** | **6.1** | **17.6** | **13.8** | **22.9** | **3.14E+08** |
| 62 | Sm_2_O_3_ | Bastnaesite, China | REOs | $ 191.19 | Oxide | USGS (2011) [84] | Samarium concentrate, at plant, CN | Ecoinvent 2.2 [4] | 2.397E+06 | 100% |  | 1160 | 915 | 1490 | 2.8 | 59.1 | 46.1 | 77.2 | 1.42E+08 |
|  | **Sm TOTAL** |  |  |  |  |  |  |  | **2.397E+06** | **100%** |  | **1160** | **915** | **1490** | **2.8** | **59.1** | **46.1** | **77.2** | **1.42E+08** |
| 63 | Eu_2_O_3_ | Bastnaesite, China | REOs | $ 1,280.00 | Oxide | USGS (2011) [84] | Europium concentrate, at plant, CN | Ecoinvent 2.2 [4] | 2.990E+05 | 100% |  | 7750 | 6020 | 10100 | 2.3 | 396 | 314 | 505 | 1.18E+08 |
|  | **Eu TOTAL** |  |  |  |  |  |  |  | **2.990E+05** | **100%** |  | **7750** | **6020** | **10100** | **2.3** | **395** | **314** | **505** | **1.18E+08** |
| 64 | Gd2O_3_ | Bastnaesite, China | REOs | $ 151.00 | Oxide | USGS (2011) [84] | Gadolinium concentrate, at plant, CN | Ecoinvent 2.2 [4] | 2.436E+06 | 100% |  | 914 | 726 | 1170 | 2.2 | 46.6 | 37.2 | 60.7 | 1.14E+08 |
|  | **Gd TOTAL** |  |  |  |  |  |  |  | **2.436E+06** | **100%** |  | **914** | **726** | **1170** | **2.2** | **46.6** | **37.2** | **60.7** | **1.14E+08** |
| 65 | Tb_4_O_7_ | Bastnaesite, China | REOs | $ 960.00 | Oxide | USGS (2011) [84] | Terbium concentrate, at plant, CN | Own assessment | 3.205E+05 | 100% |  | 5820 | 4600 | 7510 | 1.9 | 297 | 237 | 385 | 9.52E+07 |
|  | **Tb TOTAL** |  |  |  |  |  |  |  | **3.205E+05** | **100%** |  | **5820** | **4600** | **7510** | **1.9** | **297** | **237** | **385** | **9.52E+07** |
| 66 | Dy_2_O_3_ | Bastnaesite, China | REOs | $ 191.92 | Oxide | USGS (2011) [84] | Dysprosium concentrate, at plant, CN | Own assessment | 1.879E+06 | 100% |  | 1170 | 904 | 1480 | 2.2 | 59.6 | 46.9 | 76.4 | 1.12E+08 |
|  | **Dy TOTAL** |  |  |  |  |  |  |  | **1.879E+06** | **100%** |  | **1170** | **904** | **1480** | **2.2** | **59.6** | **46.9** | **76.4** | **1.12E+08** |
| 67 | Ho_2_O_3_ | Bastnaesite, China | REOs | $ 730.73 | Oxide | USGS (2011) [84] | Holmium concentrate, at plant, CN | Own assessment | 3.943E+05 | 100% |  | 4400 | 3400 | 5510 | 1.7 | 226 | 178 | 292 | 8.89E+07 |
|  | **Ho TOTAL** |  |  |  |  |  |  |  | **3.943E+05** | **100%** |  | **4400** | **3400** | **5510** | **1.7** | **226** | **178** | **292** | **8.89E+07** |
| 68 | Er_2_O_3_ | Bastnaesite, China | REOs | $ 157.29 | Oxide | USGS (2011) [84] | Erbinium concentrate, at plant, CN | Own assessment | 1.060E+06 | 100% |  | 954 | 751 | 1260 | 1.0 | 48.7 | 37.8 | 62 | 5.16E+07 |
|  | **Er TOTAL** |  |  |  |  |  |  |  | **1.060E+06** | **100%** |  | **954** | **751** | **1260** | **1.0** | **48.7** | **37.8** | **62** | **5.16E+07** |
| 69 | Tm_2_O_3_ | Bastnaesite, China | REOs | $ 2,102.10 | Oxide | USGS (2011) [84] | Thulium concentrate, at plant, CN | Own assessment | 1.787E+05 | 100% |  | 12700 | 10100 | 16200 | 2.3 | 649 | 516 | 843 | 1.16E+08 |
|  | **Tm TOTAL** |  |  |  |  |  |  |  | **1.787E+05** | **100%** |  | **12700** | **10100** | **16200** | **2.3** | **649** | **516** | **843** | **1.16E+08** |
| 70 | Yb_2_O_3_ | Bastnaesite, China | REOs | $ 404.04 | Oxide | USGS (2011) [84] | Ytterbium concentrate, at plant, CN | Own assessment | 8.440E+05 | 100% |  | 2450 | 1930 | 3080 | 2.1 | 125 | 98.1 | 161 | 1.05E+08 |
|  | **Yb TOTAL** |  |  |  |  |  |  |  | **8.440E+05** | **100%** |  | **2450** | **1930** | **3080** | **2.1** | **125** | **98.1** | **161** | **1.05E+08** |
| 71 | Lu_2_O_3_ | Bastnaesite, China | REOs | $ 2,900.00 | Oxide | USGS (2011) [84] | Lutetium concentrate, at plant, CN | Own assessment | 1.644E+05 | 100% |  | 17600 | 13500 | 22700 | 2.9 | 896 | 704 | 1150 | 1.47E+08 |
|  | **Lu TOTAL** |  |  |  |  |  |  |  | **1.644E+05** | **100%** |  | **17600** | **13500** | **22700** | **2.9** | **896** | **704** | **1150** | **1.47E+08** |
| 72 | HfO_2_ | Zircon | Zr | Mass allocation | Oxide |  | Hafnium oxide, at plant/AU U | Own assessment | 2.620E+07 | 17% | Informed estimate | 62.2 | 47.2 | 80.3 | 1.6 | 3.4 | 2.7 | 4.3 | 8.79E+07 |
|  | Hf | Zircon | Zr | $ 299.80 | Sponge | USGS (2011) [84] | Hafnium, metal ingot | Own assessment | - | 83% | Informed estimate | 4220 | 2200 | 8520 | - | 157 | 85.5 | 276 | - |
|  | **Hf TOTAL** |  |  |  |  |  |  |  | **2.620E+07** | **100%** |  | **3510** | **1900** | **7050** | **1.6** | **131** | **69** | **252** | **8.79E+07** |
| 73 | Ta | Pegmatite/Tantalite | None | NA | Metal powder |  | Tantalum powder, capacitor grade, at regional storage/GLO U | Ecoinvent 2.2 [4] | 1.274E+06 | 100% |  | 4360 | 3460 | 5570 | 5.6 | 260 | 206 | 331 | 3.31E+08 |
|  | **Ta TOTAL** |  |  |  |  |  |  |  | **-** | **100%** |  | **4360** | **3460** | **5570** | **5.6** | **260** | **206** | **331** | **3.31E+08** |
| 74 | W (ProBas) | Tungsten ore | None | NA | Metal |  | Tungsten | UBA (2010) [42] | 3.114E+07 | 50% | Own assumption | 52.4 | 44.9 | 61.5 | 1.6 | 3.9 | 3.2 | 4.7 | 1.21E+08 |
|  | W (Idemat) | Tungsten ore | None | NA | Metal |  | Tungsten I | Idemat (2001) [34] | 3.114E+07 | 50% | Own assumption | 215 | 152 | 286 | 6.7 | 21.2 | 15.3 | 29.4 | 6.61E+08 |
|  | **W TOTAL** |  |  |  |  |  |  |  | **6.228E+07** | **100%** |  | **133** | **104** | **173** | **8.3** | **12.6** | **9.6** | **16.3** | **7.82E+08** |
| 75 | Re | Molybdenite ore | Mo | $ 1,930.00 | Metal powder (99.99% pure) | USGS (2013) [32] | Rhenium, at plant, co-product of Mo roasting | Own assessment | 5.490E+04 | 100% |  | 9040 | 4470 | 16300 | 0.5 | 450 | 213 | 836 | 2.47E+07 |
|  | **Re** |  |  |  |  |  |  |  | **5.490E+04** | **100%** |  | **9040** | **4470** | **16300** | **0.5** | **450** | **213** | **836** | **2.47E+07** |
| 76 | Os (ZA) | Bushveld Mine, PGM-dominated | Cu, Ni, PGMs | $ 11,397.44 | Metal | USGS (2011) [84] | _Osmium, primary, at refinery/ZA U, reallocated | Own assessment | 1.381E+03 | 37% | USGS (2010) [26] | 81700 | 56600 | 112000 | 0.1 | 4130 | 3080 | 5390 | 5.71E+06 |
|  | Os (RU) | Noril'sk Mine, Ni-Cu-dominated | Cu, Ni, PGMs | $ 11,397.44 | Metal | USGS (2011) [84] | _Osmium, primary, at refinery/RU U, reallocated | Own assessment | 2.342E+03 | 63% | USGS (2010) [26] | 86900 | 65900 | 117000 | 0.2 | 4810 | 3840 | 6100 | 1.13E+07 |
|  | **Os TOTAL** |  |  |  |  |  |  |  | **3.723E+03** | **100%** |  | **85000** | **64600** | **110000** | **0.3** | **4560** | **3700** | **5650** | **1.70E+07** |
| 77 | Ir (ZA) | Bushveld Mine, PGM-dominated | Cu, Ni, PGMs | $ 15,919.87 | Metal | USGS (2011) [84] | _Iridium, primary, at refinery/ZA U, reallocated | Own assessment | 6.856E+03 | 61% | USGS (2010) [26] | 165000 | 118000 | 220000 | 1.1 | 8340 | 6260 | 10800 | 5.72E+07 |
|  | Ir (RU) | Noril'sk Mine, Ni-Cu-dominated | Cu, Ni, PGMs | $ 15,919.87 | Metal | USGS (2011) [84] | _Iridium, primary, at refinery/RU U, reallocated | Own assessment | 4.476E+03 | 40% | USGS (2010) [26] | 175000 | 130000 | 224000 | 0.8 | 9670 | 7580 | 12600 | 4.33E+07 |
|  | **Ir TOTAL** |  |  |  |  |  |  |  | **1.133E+04** | **100%** |  | **169000** | **128000** | **219000** | **1.9** | **8860** | **7000** | **11200** | **1.00E+08** |
| 78 | Pt (ZA) | Bushveld Mine, PGM-dominated | Cu, Ni, PGMs | $ 43,627.17 | Metal | USGS (2011) [84] | _Platinum, primary, at refinery/ZA U, reallocated | Ecoinvent 2.2 [4] | 1.568E+05 | 63% | USGS (2010) [26] | 308000 | 219000 | 414000 | 48.3 | 15600 | 11700 | 21000 | 2.44E+09 |
|  | Pt (RU) | Noril'sk Mine, Ni-Cu-dominated | Cu, Ni, PGMs | $ 43,627.17 | Metal | USGS (2011) [84] | _Platinum, primary, at refinery/RU U, reallocated | Ecoinvent 2.2 [4] | 3.571E+04 | 14% | USGS (2010) [26] | 327000 | 245000 | 430000 | 11.7 | 18100 | 14300 | 23300 | 6.46E+08 |
|  | Pt (secondary) | Catalysts, etc. | Pd, Rh | $ 43,627.17 | Metal | USGS (2011) [84] | Platinum, secondary, at refinery/RER U reallocated (Secondary) | Ecoinvent 2.2 [4] | - | 23% | USGS (2010) [26] | 11900 | 8290 | 16900 | - | 634 | 474 | 853 | - |
|  | **Pt TOTAL** |  |  |  |  |  |  |  | **1.926E+05** | **100%** |  | **243000** | **178000** | **326000** | **59.9** | **12500** | **9650** | **16200** | **3.09E+09** |
| 79 | Au | Gold ores | None | NA | Metal |  | Gold, primary, from gold-ore, at refinery/GLO U | Ecoinvent 2.2 [4] | 1.481E+06 | 42% | Nassar et al (2012); Classen et al (2009) [13,43] | 245000 | 199000 | 315000 | 362 | 13600 | 11100 | 16800 | 2.01E+10 |
|  | Au (from Au-Ag) | Gold-Silver ore | Ag | $ 28,170.48 | Metal | USGS (2011) [84] | Gold, from combined gold-silver production, at refinery/GLO U | Ecoinvent 2.2 [4] | 7.948E+05 | 23% | Nassar et al (2012); Classen et al (2009) [13,43] | 447000 | 307000 | 629000 | 355 | 28200 | 19600 | 39500 | 2.24E+10 |
|  | Au (secondary) | Scrap | Ag, Pd | $ 28,170.48 | Metal |  | Gold, secondary, at precious metal refinary/SE U | Ecoinvent 2.2 [4] | - | 35% | Nassar et al (2012); Classen et al (2009) [13,43] | 8120 | 3690 | 17800 | - | 922 | 352 | 2260 | - |
|  | **Au TOTAL** |  |  |  |  |  |  |  | **2.276E+06** | **100%** |  | **208000** | **165000** | **263000** | **718** | **12500** | **10100** | **15400** | **4.25E+10** |
| 80 | Hg | Cinnibar | None | NA | Metal |  | Mercury, liquid, at plant/GLO U | Ecoinvent 2.2 [4] | 1.817E+06 | 70% | EC (2004) [77] | 179 | 110 | 280 | 0.3 | 12.1 | 7.7 | 18.6 | 2.21E+07 |
|  | Hg | Various ores | Au, Ag, Cu, Zn |  | Metal |  | Mercury, by-product at plant | Not included due to a lack of data | - | ? | EC (2004) [77] | - | - | - | - | - | - | - | - |
|  | Hg | Residual mercury | None |  | Metal |  | Mercury, recovered at plant | Not included due to a lack of data | - | 30% | EC (2004) [77] | - | - | - | - | - | - | - | - |
|  | **Hg TOTAL** |  |  |  |  |  |  | **Based on mercury from cinnabar only** | **1.817E+06** | **100%** |  | **179** | **110** | **280** | **0.3** | **12.1** | **7.7** | **18.6** | **2.21E+07** |
| 81 | Tl | Zinc ores | Zn | ProBas allocation used | Metal |  | Thallium | UBA (2010) [42] | 1.000E+04 | 100% | USGS (2010) [64] | 5160 | 4220 | 6180 | 0.1 | 376 | 189 | 681 | 3.76E+06 |
|  | **Tl TOTAL** |  |  |  |  |  |  |  |  | **100%** |  | **5160** | **4220** | **6180** | **0.1** | **376** | **291** | **469** | **3.76E+06** |
| 82 | Pb (from Pb-Zn) | Lead/Zinc ores | Zn, Au (parkes crust) | $ 2.26 | Metal | USGS (2011) [84] | Lead, primary, at plant/GLO U reallocated | Ecoinvent 2.2 [4] | 7.649E+09 | 40% | USGS (2010); RMG (2006) [26,38] | 27.9 | 22.6 | 35.5 | 213 | 2.4 | 2 | 2.8 | 1.80E+10 |
|  | Pb (from Au-Ag) | Au-Ag ores | Au, Ag, Cu, Zn | $ 2.26 | Metal | USGS (2011) [84] | Lead, from combined metal production, at refinery/SE U | Ecoinvent 2.2 [4] | 4.160E+08 | 6% | USGS (2010); RMG (2006) [26,38] | 28.9 | 22.8 | 37.8 | 12.0 | 0.8 | 0.7 | 0.9 | 3.13E+08 |
|  | Pb (secondary) | Scrap | None | $ 2.26 | Metal |  | Lead, secondary, at plant/RER U | Ecoinvent 2.2 [4] | - | 50% | USG (2010) [26] | 11.9 | 9.1 | 15.5 | 0.0 | 0.7 | 0.5 | 0.8 | - |
|  | Pb (secondary, E-scrap) | Electronic scrap | Au, Ag, Pd, Ni, Cu | $ 2.26 | Metal | USGS (2011) [84] | Lead, secondary, from electronic and electric scrap recycling, at plant/SE U, reallocated | Ecoinvent 2.2 [4] | - | 4% | Own assumption | 0.4 | 0.3 | 0.4 | - | 0 | 0 | 0.1 | - |
|  | **Pb TOTAL** |  |  |  |  |  |  |  | **8.065E+09** | **100%** |  | **18.9** | **15.8** | **23.5** | **225** | **1.3** | **1.1** | **1.5** | **1.83E+10** |
| 83 | Bi (Probas) | Lead-Zinc ores | Pb, Cu, Ag, W, Sn, Mo | $ 21.12 | Metal | USGS (2011) [84] | Bismuth - ProBas | UBA (2010) [42] | - | 0% | own assumption | 211 | 186 | 241 | - | 19.8 | 19.4 | 23.1 | - |
|  | Bi (Canada) | Lead-Zinc ores | Pb, Cu, Ag, W, Sn, Mo | $ 21.12 | Metal | USGS (2011) [84] | Bismuth | Andrae et al (2008) [83] | - | 0% | own assumption | 336 | 299 | 379 | - | 21.8 | 19.3 | 25.5 | - |
|  | Bi (World) | Lead-Zinc ores | Pb, Cu, Ag, W, Sn, Mo | $ 21.12 | Metal | USGS (2011) [84] | Bismuth - updated to 2008 World | Andrae et al (2008) [83] | 1.660E+07 | 100% | own assumption | 697 | 332 | 2320 | 11.6 | 58.9 | 53.7 | 66.5 | 9.78E+08 |
|  | **Bi TOTAL** |  |  |  |  |  |  | **Based on Bi (World)** | **1.660E+07** | **100%** |  | **697** | **332** | **2320** | **11.6** | **58.9** | **53.7** | **66.5** | **9.78E+08** |
| 90 | ThO_2_ | Monazite | REOs | $ 226.20 | Oxide | USGS (2011) [84] | Thorium oxide (ThO2) | Own assessment | 5.417E+05 | 100% | own assumption | 1260 | 960 | 1670 | 0.70 | 74.9 | 62.4 | 91.8 | 4.06E+07 |
|  | **Th TOTAL** |  |  |  |  |  |  |  | **5.417E+05** | **100%** |  | **1260** | **960** | **1670** | **0.70** | **74.9** | **62.4** | **91.8** | **4.06E+07** |
| 92 | UO_2_ | Uranium-containing ores | None | NA | Oxide |  | Uranium natural, in yellowcake, at mill plant/RNA U | Ecoinvent 2.2 [4] | 5.179E+07 | 100% | own assumption | 1270 | 826 | 2000 | 65.8 | 90.7 | 61.3 | 140 | 4.70E+09 |
|  | **U TOTAL** |  |  |  |  |  |  |  | **5.179E+07** | **100%** |  | **1270** | **826** | **2000** | **65.8** | **90.7** | **61.3** | **140** | **4.70E+09** |

Z = Atomic number. NA = Not applicable ZA = South Africa. RU = Russia. Metal TOTAL = The weight-averaged global warming potential (GWP) or cumulative energy demand (CED) associated with providing 1 kg of each element at the factory gate. This takes into account the chemical forms of an element going into use and/or different production routes (see weighting references and discussion for each element in earlier sections of this report). The weight-averaged environmental impacts are reported in Figure 2 and 3 of the main manuscript. Global impacts are derived by multiplying the per kg environmental impacts with 2008 global annual production figures. Overall results for global impacts are given in Figure 5, Figure 6, and Table 1 of the main manuscript.

1. **References**

1. Weidema BP, Wesnæs MS (1996) Data quality management for life cycle inventories--an example of using data quality indicators. J Clean Prod 4: 167–174. Available: http://www.sciencedirect.com/science/article/B6VFX-3VWNFWR-88/2/bf4935bdacfacd37407450a7973bd3d2. Accessed 9 November 2010.

2. Frischknecht R, Jungbluth N (2007) Ecoinvent - Overview and Methodology Data v2.0. Dübendorf, CH: Swiss Centre for Life Cycle Inventories.

3. Häussinger P, Glatthaar R, Rhode W, Kick H, Benkmann C, et al. (2000) Noble Gases. Ullmann’s Encyclopedia of Industrial Chemistry. Wiley-VCH Verlag GmbH & Co. KGaA. Available: http://onlinelibrary.wiley.com/doi/10.1002/14356007.a17_485/abstract. Accessed 11 October 2013.

4. Ecoinvent (2010) Ecoinvent Life Cycle Inventory database v2.2. Swiss Centre for Life Cycle Inventories. Available: http://www.ecoinvent.ch/. Accessed 7 November 2010.

5. Sutter J (2007) Life Cycle Inventories of Highly Pure Chemicals. Dübendorf, CH: Ecoinvent Centre, ETh Zurich.

6. Goonan T (2012) Lithium use in batteries. U.S. Geological Survey (USGS). Available: http://pubs.usgs.gov/circ/1371/pdf/circ1371_508.pdf. Accessed 23 April 2014.

7. Gruber PW, Medina PA, Keoleian GA, Kesler SE, Everson MP, et al. (2011) Global Lithium Availability. J Ind Ecol 15: 760–775. Available: http://onlinelibrary.wiley.com/doi/10.1111/j.1530-9290.2011.00359.x/abstract. Accessed 9 May 2013.

8. USGS (2011) 2011 Minerals Yearbook. Reston, VA: U.S. Geological Survey.

9. Cunningham LD (2004) Beryllium Recycling in the United States in 2000. In: Sibley SF, editor. Flow Studies for Recycling Metal Commodities in the United States. Reston, VA: U.S. Department of the Interior, U.S. Geological Survey (USGS). Available: http://pubs.usgs.gov/circ/c1196p/c1196p.pdf. Accessed 28 August 2013.

10. Petzow G, Aldinger F, Jönsson S, Welge P, van Kampen V, et al. (2000) Beryllium and Beryllium Compounds. Ullmann’s Encyclopedia of Industrial Chemistry. Wiley-VCH Verlag GmbH & Co. KGaA. Available: http://onlinelibrary.wiley.com/doi/10.1002/14356007.a04_011.pub2/abstract. Accessed 15 December 2012.

11. BeST (2013) Beryllium Facts and Figures. Beryllium Sci Technol Assoc BeST. Available: http://beryllium.eu/about-beryllium-and-beryllium-alloys/facts-and-figures/beryllium-extraction/. Accessed 28 August 2013.

12. Mc Neil D (2013) Beryllium. Roskill Mineral Services. Available: http://beryllium.eu/?wpfb_dl=30. Accessed 28 August 2013.

13. Classen M, Althaus H-J, Blaser S, Scharnhorst W, Tuchschmidt M, et al. (2009) Life Cycle Inventories of Metals, Data v2.0. Dübendorf, CH: Ecoinvent Centre, ETh Zurich.

14. Materion (2013) Personal communication. Materion Corporation.

15. USGS (2008) 2008 Minerals Yearbook. Reston, VA: U.S. Geological Survey.

16. Smith RA (2000) Boric Oxide, Boric Acid, and Borates. Ullmann’s Encyclopedia of Industrial Chemistry. Wiley-VCH Verlag GmbH & Co. KGaA. Available: http://onlinelibrary.wiley.com/doi/10.1002/14356007.a04_263/abstract. Accessed 10 October 2013.

17. Althaus H-J, Hischier R, Osses M, Primas A, Hellweg S, et al. (2007) Life Cycle Inventories of Chemicals Data v2.0. Dübendorf, CH: Ecoinvent Centre, ETh Zurich.

18. Graedel TE, Harper EM, Nassar NT, Reck BK (2013) On the materials basis of modern society. Proc Natl Acad Sci: 201312752. Available: http://www.pnas.org/content/early/2013/11/27/1312752110. Accessed 16 December 2013.

19. BGS (2004) Commodity Profile Magnesium. Nottingham, UK: British Geological Survey. Available: https://www.bgs.ac.uk/mineralsuk/statistics/mineralProfiles.html. Accessed 23 April 2014.

20. Kenny M, Oates T (2007) Lime and Limestone. Ullmann’s Encyclopedia of Industrial Chemistry. Wiley-VCH Verlag GmbH & Co. KGaA. Available: http://onlinelibrary.wiley.com/doi/10.1002/14356007.a15_317.pub2/abstract. Accessed 9 September 2013.

21. Kellenberger D, Althaus HJ, Kuenniger T, Lehmann M (2007) Life Cycle Inventory of Building Products. Duebendorf, Switzerland.

22. Granta Design (2012) Granta: CES Selector materials selection software. Available: http://www.grantadesign.com/products/ces/. Accessed 13 August 2012.

23. Hluchan SE, Pomerantz K (2006) Calcium and Calcium Alloys. Ullmann’s Encyclopedia of Industrial Chemistry. Wiley-VCH Verlag GmbH & Co. KGaA. Available: http://onlinelibrary.wiley.com/doi/10.1002/14356007.a04_515.pub2/abstract. Accessed 9 September 2013.

24. Vrana LM (2000) Calcium and Calcium Alloys. Kirk-Othmer Encyclopedia of Chemical Technology. John Wiley & Sons, Inc. Available: http://onlinelibrary.wiley.com/doi/10.1002/0471238961.0301120308090202.a01.pub3/abstract. Accessed 4 September 2013.

25. World Bank (2012) World Data Bank. World Dev Indic WDI Glob Dev Finance GDF. Available: http://databank.worldbank.org/ddp/home.do?Step=12&id=4&CNO=2. Accessed 31 October 2012.

26. USGS (2010) 2010 Minerals Yearbook. Reston, Virginia: U.S. Department of the Interior, U.S. Geological Survey.

27. InfoMine (2011) Calcium Metal: Production, Market and Forecast in Russia and in the World. Moscow: InfoMine Research Group. Available: http://eng.infomine.ru/files/catalog/380/file_380_eng.pdf. Accessed 21 October 2013.

28. USGS (2009) Mineral Commodity Summaries 2009. Reston, VA: U.S. Geological Survey. Available: http://minerals.er.usgs.gov/minerals/pubs/commodity/. Accessed 31 August 2012.

29. McGill I (2000) Rare Earth Elements. Ullmann’s Encyclopedia of Industrial Chemistry. Wiley-VCH Verlag GmbH & Co. KGaA. Available: http://onlinelibrary.wiley.com/doi/10.1002/14356007.a22_607/abstract. Accessed 8 November 2012.

30. USGS (2012) 2012 Minerals Yearbook. Reston, VA: U.S. Geological Survey.

31. Wang W, Pranolo Y, Cheng CY (2011) Metallurgical processes for scandium recovery from various resources: A review. Hydrometallurgy 108: 100–108. Available: http://www.sciencedirect.com/science/article/pii/S0304386X11000648. Accessed 6 September 2013.

32. USGS (2013) Mineral Commodity Summaries 2013. Reston, VA: U.S. Geological Survey. Available: http://minerals.er.usgs.gov/minerals/pubs/commodity/. Accessed 31 August 2012.

33. Ashby MF, Miller A, Rutter F, Seymour C, Wegst UGK (2012) CES EduPack for Eco Design — A White Paper. Cambridge, UK: Granta Design Ltd.

34. TU Delft (2001) IdEMAT Life Cycle Inventory Database. Available: http://www.idemat.nl/. Accessed 27 August 2012.

35. Ceresana (2013) Marktstudie Titandioxid (UC-4705). Konstanz, Germany: Ceresana Market Intelligence Consulting. Available: http://www.ceresana.com/de/marktstudien/chemikalien/titandioxid/marktstudie-titandioxid.html. Accessed 23 April 2014.

36. Woolery M (2000) Vanadium Compounds. Kirk-Othmer Encyclopedia of Chemical Technology. John Wiley & Sons, Inc. Available: http://onlinelibrary.wiley.com/doi/10.1002/0471238961.2201140123151512.a01.pub2/abstract. Accessed 18 September 2012.

37. Liddell N, Spring S, Welch C, Lucas A (2011) Vanadium Sector Review. Ocean Equities Ltd. Available: http://www.niplats.com.au/media/articles/Investor---Research/20110719-Ocean-Equities-Vanadium-Sector-Review---July-2011-261/Vanadium-Thematic-Ocean-Equities-Research-July-2011.pdf. Accessed 18 September 2012.

38. RMG (2006) Raw Materials Data Software - The Mining Database. Stockholm, Sweden: Raw Materials Group.

39. Greber JF (2000) Gallium and Gallium Compounds. Ullmann’s Encyclopedia of Industrial Chemistry. Wiley-VCH Verlag GmbH & Co. KGaA. Available: http://onlinelibrary.wiley.com/doi/10.1002/14356007.a12_163/abstract. Accessed 10 November 2012.

40. Harper E, Kavlak G, Burmeister L, Eckelman M, Erbis S, et al. (2014) Criticality of the geological zinc, tin, and lead family. Rev.

41. Grund SC, Hanusch K, Wolf HU (2000) Arsenic and Arsenic Compounds. Ullmann’s Encyclopedia of Industrial Chemistry. Wiley-VCH Verlag GmbH & Co. KGaA. Available: http://onlinelibrary.wiley.com/doi/10.1002/14356007.a03_113.pub2/abstract. Accessed 14 October 2013.

42. UBA (2010) ProBas - Lebenszyklusdatenbank [Life Cycle Inventory Database]. Dessau (Germany); Freiburg (Germany): Umweltbundesamt (German Federal Environmental Agency) and Öko-Institut. Available: http://www.probas.umweltbundesamt.de/php/index.php. Accessed 22 November 2010.

43. Nassar NT, Barr R, Browning M, Diao Z, Friedlander E, et al. (2012) Criticality of the Geological Copper Family. Environ Sci Technol 46: 1071–1078. Available: http://dx.doi.org/10.1021/es203535w.

44. MacMillan JP, Park JW, Gerstenberg R, Wagner H, Köhler K, et al. (2000) Strontium and Strontium Compounds. Ullmann’s Encyclopedia of Industrial Chemistry. Wiley-VCH Verlag GmbH & Co. KGaA. Available: http://onlinelibrary.wiley.com/doi/10.1002/14356007.a25_321/abstract. Accessed 9 September 2012.

45. Hibbins SG (2000) Strontium and Strontium Compounds. Kirk-Othmer Encyclopedia of Chemical Technology. John Wiley & Sons, Inc. Available: http://onlinelibrary.wiley.com/doi/10.1002/0471238961.1920181508090202.a01.pub2/abstract. Accessed 9 September 2012.

46. Coope B (2006) Spain’s celestite celebration. Industrial Minerals (IM). Available: http://www.indmin.com/pdfs/697/67081/200607042.pdf. Accessed 3 September 2013.

47. Jungbluth N (2007) Erdöl. In: Sachbilanzen von Energiesystemen: Grundlagen für den ökologischen Vergleich von Energiesystemen und den Einbezug von Energiesystemen in Ökobilanzen für die Schweiz. Duebendorf, Switzerland: Ecoinvent Centre.

48. Nielsen RH, Wilfing G (2000) Zirconium and Zirconium Compounds. Ullmann’s Encyclopedia of Industrial Chemistry. Wiley-VCH Verlag GmbH & Co. KGaA. Available: http://onlinelibrary.wiley.com/doi/10.1002/14356007.a28_543.pub2/abstract. Accessed 27 August 2012.

49. Gambogi J (2011) Zirconium and Hafnium. 2010 Minerals Yearbook. U.S. Geological Service (USGS).

50. Nielsen RH, Wilfing G (2000) Hafnium and Hafnium Compounds. Ullmann’s Encyclopedia of Industrial Chemistry. Wiley-VCH Verlag GmbH & Co. KGaA. Available: http://onlinelibrary.wiley.com/doi/10.1002/14356007.a12_559.pub2/abstract. Accessed 27 August 2012.

51. Lundberg M (2012) Environmental analysis of zirconium alloy production. Available: http://uu.diva-portal.org/smash/record.jsf?pid=diva2:475527. Accessed 27 August 2012.

52. Cardarelli F (2008) Materials Handbook: A Concise Desktop Reference. Springer. 1365 p.

53. Miller GL (1954) Arc melting Kroll zirconium sponge. Vacuum 4: 168–175. Available: http://www.sciencedirect.com/science/article/pii/S0042207X54800066. Accessed 22 August 2012.

54. Munnoch S (2012) Hafnium. Minor Metals Trade Association (MMTA). Available: http://www.mmta.co.uk/uploaded_files/Hafnium%20MJ.pdf. Accessed 27 August 2012.

55. USGS (2011) Zirconium and Hafnium. 2011 Mineral Commodity Summaries. U.S. Geological Survey. Available: http://minerals.usgs.gov/minerals/pubs/commodity/zirconium/mcs-2011-zirco.pdf. Accessed 27 August 2012.

56. IAMGOLD (2009) Niobec Tour Presentation. IAMGOLD Corporation. Available: http://www.iamgold.com/Theme/IAmGold/files/presentations/Niobec%20Tour%202009%20-%20%20Niobec%20Presentation%20FINAL.pdf. Accessed 17 September 2012.

57. Albrecht S, Cymorek C, Eckert J (2000) Niobium and Niobium Compounds. Ullmann’s Encyclopedia of Industrial Chemistry. Wiley-VCH Verlag GmbH & Co. KGaA. Available: http://onlinelibrary.wiley.com/doi/10.1002/14356007.a17_251.pub2/abstract. Accessed 16 September 2012.

58. Nuss P, Harper EM, Nassar NT, Reck BK, Graedel TE (2014) Criticality of Iron and Its Principal Alloying Elements. Environ Sci Technol 48: 4171–4177. Available: http://dx.doi.org/10.1021/es405044w. Accessed 10 April 2014.

59. IPPC (2001) Reference Document on Best Available Techniques in the Non Ferrous Metals Industries. Integrated Pollution Prevention and Control (IPPC), European Commission. Available: http://circa.europa.eu/Public/irc/env/ippc_brefs/library?l=/bref_ferrous_metals_1/ferrous_metals_enpdf/_EN_1.0_&a=d. Accessed 15 September 2012.

60. Eckelman M, Ciacci L, Kavlak G, Reck B, Graedel T (2011) Estimating Carbon Credits for Aerospace Alloys. New Haven, CT: Center for Industrial Ecology, Yale University.

61. BIO Intelligence Service (2010) Environmental Impacts of some Raw Materials through LCA Methods. Paris.

62. Graf GG (2000) Tin, Tin Alloys, and Tin Compounds. Ullmann’s Encyclopedia of Industrial Chemistry. Wiley-VCH Verlag GmbH & Co. KGaA. Available: http://onlinelibrary.wiley.com/doi/10.1002/14356007.a27_049/abstract. Accessed 10 November 2012.

63. Kresse R, Baudis U, Jäger P, Riechers HH, Wagner H, et al. (2000) Barium and Barium Compounds. Ullmann’s Encyclopedia of Industrial Chemistry. Wiley-VCH Verlag GmbH & Co. KGaA. Available: http://onlinelibrary.wiley.com/doi/10.1002/14356007.a03_325.pub2/abstract. Accessed 9 September 2012.

64. USGS (2010) Mineral Commodity Summaries 2010. Reston, VA: U.S. Geological Survey. Available: http://minerals.er.usgs.gov/minerals/pubs/commodity/. Accessed 31 August 2012.

65. Chinese Society of Rare Earths (2010) Chinese Rare Earth Yearbook 2010 (in Chinese). Beijing, China.

66. Du X, Graedel TE (2011) Global In-Use Stocks of the Rare Earth Elements: A First Estimate. Environ Sci Technol 45: 4096–4101. Available: http://dx.doi.org/10.1021/es102836s. Accessed 22 September 2013.

67. Peiró LT, Méndez GV (2013) Material and Energy Requirement for Rare Earth Production. JOM 65: 1327–1340. Available: http://link.springer.com/article/10.1007/s11837-013-0719-8. Accessed 4 May 2014.

68. Gupta CK, Krishnamurthy N (2004) Extractive Metallurgy of Rare Earths. CRC Press. 537 p.

69. Dahmus J, Gutowski T (2004) An environmental analysis of machining Anaheim, California, USA. Available: http://stuff.mit.edu/afs/athena.mit.edu/course/2/2.810/www/ASME2004-62600.pdf. Accessed 8 April 2013.

70. Millensifer TA (2000) Rhenium and Rhenium Compounds. Kirk-Othmer Encyclopedia of Chemical Technology. John Wiley & Sons, Inc. Available: http://onlinelibrary.wiley.com/doi/10.1002/0471238961.1808051420180509.a01.pub3/abstract. Accessed 23 July 2013.

71. Vermaak CF (1995) Platinum-group metals: a global perspective. Mintek. 312 p.

72. CPM (2012) The CPM Platinum Group Metals Yearbook 2012. 26th ed. New York: Euromoney Books. Available: http://www.cpmgroup.com/shop/product/precious-metals/cpm-platinum-group-metals-yearbook-2012. Accessed 24 September 2013.

73. Johnson Matthey (2013) Platinum 2013. Royston, United Kingdom: Johnson Matthey.

74. Naldrett AJ (2011) Fundamentals of Magmatic Sulfide Deposits. In: Li C, Ripley EM, editors. Magmatic Ni-Cu and PGE deposits: geology, geochemistry, and genesis. Reviews in economic geology. Littleton, CO: Society of Economic Geologists. pp. 1–50.

75. USBM (1985) Mineral Facts and Problems: 1985 Edition. Washington D.C.: U.S. Department of the Interior, U.S. Bureau of Mines.

76. DeVito SC, Brooks WE (2000) Mercury. Kirk-Othmer Encyclopedia of Chemical Technology. John Wiley & Sons, Inc. Available: http://onlinelibrary.wiley.com/doi/10.1002/0471238961.1305180304052209.a01.pub2/abstract. Accessed 8 September 2012.

77. EC (2004) Mercury Flows in Europe and the World: The Impacts of Decommissioned Chlor-Alkali Plants. Brussels: European Commission. Available: http://ec.europa.eu/environment/chemicals/mercury/pdf/report.pdf. Accessed 8 September 2012.

78. Micke H, Wolf HU (2000) Thallium and Thallium Compounds. Ullmann’s Encyclopedia of Industrial Chemistry. Wiley-VCH Verlag GmbH & Co. KGaA. Available: http://onlinelibrary.wiley.com/doi/10.1002/14356007.a26_607/abstract. Accessed 8 September 2012.

79. Giegrich J, Liebich A, Lauwigi C, Reinhardt J (2012) Indikatoren / Kennzahlen für den Rohstoffverbrauch im Rahmen der Nachhaltigkeits-diskussion. Dessau-Roßlau, Germany: UMWELTBUNDESAMT. Available: http://www.uba.de/uba-info-medien/4237.html. Accessed 5 November 2012.

80. USGS (1999) Metal Prices in the United States Through 1998. Reston, VA: U.S. Geological Survey. Available: http://minerals.usgs.gov/minerals/pubs/metal_prices/metal_prices1998.pdf. Accessed 5 November 2012.

81. Sutherland CA, Milner EF, Kerby RC, Teindl H, Melin A, et al. (2000) Lead. Ullmann’s Encyclopedia of Industrial Chemistry. Wiley-VCH Verlag GmbH & Co. KGaA. Available: http://onlinelibrary.wiley.com/doi/10.1002/14356007.a15_193.pub2/abstract. Accessed 31 May 2013.

82. Naumov A (2007) World market of bismuth: A review. Russ J Non-Ferr Met 48: 10–16. Available: http://www.springerlink.com/content/n300673l6p7n271n/abstract/. Accessed 7 September 2012.

83. Andrae ASG, Itsubo N, Yamaguchi H, Inaba A (2008) Life Cycle Assessment of Japanese High-Temperature Conductive Adhesives. Environ Sci Technol 42: 3084–3089. Available: http://dx.doi.org/10.1021/es0709829.

84. USGS (2011) Mineral Commodity Summaries 2011. Reston, VA: U.S. Geological Survey. Available: http://minerals.er.usgs.gov/minerals/pubs/commodity/. Accessed 31 August 2012.

85. Stoll W (2000) Thorium and Thorium Compounds. Ullmann’s Encyclopedia of Industrial Chemistry. Wiley-VCH Verlag GmbH & Co. KGaA. Available: http://onlinelibrary.wiley.com/doi/10.1002/14356007.a27_001/abstract. Accessed 27 August 2012.

86. Cuney M (2012) Uranium and Thorium: The Extreme Diversity of the Resources of the World’s Energy Minerals. In: Sinding-Larsen R, Wellmer F-W, editors. Non-Renewable Resource Issues. International Year of Planet Earth. Springer Netherlands. pp. 91–129. Available: http://link.springer.com/chapter/10.1007/978-90-481-8679-2_6. Accessed 21 March 2013.

87. Schmidt G (2013) Description and critical environmental evaluation of the REE refining plant LAMP near Kuantan/Malaysia. Darmstadt, Germany: Oeko Institute e.V. Available: http://www.oeko.de/oekodoc/1628/2013-001-en.pdf. Accessed 18 March 2013.

88. Roskill (2005) The Economics of Magnesium Compounds and Chemicals. London: Roskill Information Services.

89. Rombach G (2013) Raw material supply by aluminium recycling – Efficiency evaluation and long-term availability. Acta Mater 61: 1012–1020. Available: http://www.sciencedirect.com/science/article/pii/S1359645412006386. Accessed 7 June 2013.

90. Ecoinvent (2013) Ecoinvent Life Cycle Inventory database v3.0. Swiss Centre for Life Cycle Inventories. Available: http://www.ecoinvent.ch/. Accessed 26 January 2014.

91. Anger G, Halstenberg J, Hochgeschwender K, Scherhag C, Korallus U, et al. (2000) Chromium Compounds. Ullmann’s Encyclopedia of Industrial Chemistry. Wiley-VCH Verlag GmbH & Co. KGaA. Available: http://onlinelibrary.wiley.com/doi/10.1002/14356007.a07_067/abstract. Accessed 23 October 2012.

92. USGS (2009) 2009 Minerals Yearbook. Reston, Virginia: U.S. Department of the Interior, U.S. Geological Survey.

93. Reuters (2013) Copper treatment charge benchmark established at $70/T -Aurubis. Reuters. Available: http://www.reuters.com/article/2013/03/21/copper-market-aurubis-idUSL6N0CDCNQ20130321. Accessed 23 September 2013.

94. BGS (2011) Niobium-Tantalum Minerals Profile. Nottingham, UK: British Geological Service. Available: http://www.bgs.ac.uk/mineralsuk/statistics/mineralProfiles.html. Accessed 16 April 2013.

95. SMR (2009) Molybdenum End Use Analysis 2008. Reutte, Austria: SMR GmbH, Steel & Metals Market Research.

96. Thomson Reuters (2012) World Silver Survey 2012 - A Summary. Washington D.C.: The Silver Institute. Available: https://www.silverinstitute.org/site/wp-content/uploads/2012/07/wss12sum.pdf. Accessed 22 October 2013.

1. <http://www.reuters.com/article/2013/03/21/copper-market-aurubis-idUSL6N0CDCNQ20130321> (accessed September 2013). [↑](#footnote-ref-1)
2. This inventory represents the primary production of copper (roasting and smelting) with the refining stage being omitted. The copper content of blister copper is 98%. [↑](#footnote-ref-2)
3. “Niob- und Tantalkonzentrate” [↑](#footnote-ref-3)
4. Consisting of calcium carbonates, oxides and hydroxides. [↑](#footnote-ref-4)
5. Current work of the Yale criticality project addresses this issue by collecting historical global production data for all six PGMs by country. This data will be used together with updated emission factor for the PGM refining process in a forthcoming publication discussing the environmental implications of PGMs as part of a wider criticality assessment. [↑](#footnote-ref-5)
6. The unit process “Electricity, medium voltage, production UCTE, at grid UCTE/U” is used. [↑](#footnote-ref-6)
